# Investigating differential effects of socio-emotional and mindfulness-based online interventions on mental health, resilience and social capacities during the COVID-19 pandemic: The study protocol

**DOI:** 10.1371/journal.pone.0256323

**Published:** 2021-11-04

**Authors:** Malvika Godara, Sarita Silveira, Hannah Matthäus, Christine Heim, Manuel Voelkle, Martin Hecht, Elisabeth B. Binder, Tania Singer

**Affiliations:** 1 Social Neuroscience Lab, Max Planck Society, Berlin, Germany; 2 Charité – Universitätsmedizin, Corporate Member of the Free University of Berlin and Humboldt University of Berlin, Institute for Medical Psychology, Berlin, Germany; 3 Institute of Psychology, Humboldt University of Berlin, Berlin, Germany; 4 Hector Research Institute of Education Sciences and Psychology, University of Tübingen, Tübingen, Germany; 5 Department for Translational Research in Psychiatry, Max Planck Institute of Psychiatry, Munich, Germany; Public Library of Science, UNITED STATES

## Abstract

**Background:**

The SARS-CoV-2 pandemic has led to a mental health crisis on a global scale. Epidemiological studies have reported a drastic increase in mental health problems, such as depression and anxiety, increased loneliness and feelings of disconnectedness from others, while resilience levels have been negatively affected, indicating an urgent need for intervention. The current study is embedded within the larger CovSocial project which sought to evaluate longitudinal changes in vulnerability, resilience and social cohesion during the pandemic. The current second phase will investigate the efficacy of brief online mental training interventions in reducing mental health problems, and enhancing psychological resilience and social capacities. It further provides a unique opportunity for the prediction of intervention effects by individual biopsychosocial characteristics and preceding longitudinal change patterns during the pandemic in 2020/21.

**Methods:**

We will examine the differential effects of a socio-emotional (including ‘Affect Dyad’) and a mindfulness-based (including ‘Breathing Meditation’) intervention, delivered through a web- and cellphone application. Participants will undergo 10 weeks of intervention, and will be compared to a retest control group. The effectiveness of the interventions will be evaluated in a community sample (*N* = 300), which is recruited from the original longitudinal CovSocial sample. The pre- to post-intervention changes, potential underlying mechanisms, and prediction thereof, will be assessed on a wide range of outcomes: levels of stress, loneliness, depression and anxiety, resilience, prosocial behavior, empathy, compassion, and the impact on neuroendocrine, immunological and epigenetic markers. The multi-method nature of the study will incorporate self-report questionnaires, behavioral tasks, ecological momentary assessment (EMA) approaches, and biological, hormonal and epigenetic markers assessed in saliva.

**Discussion:**

Results will reveal the differential effectiveness of two brief online interventions in improving mental health outcomes, as well as enhancing social capacities and resilience. The present study will serve as a first step for future application of scalable, low-cost interventions at a broader level to reduce stress and loneliness, improve mental health and build resilience and social capacities in the face of global stressors.

**Trial registration:**

This trial has been registered on May 17, 2020 with the ClinicalTrials.gov NCT04889508 registration number (clinicaltrials.gov/ct2/show/NCT04889508).

## Introduction

The SARS-CoV-2 pandemic has been one of the most challenging global health crises to have impacted the 21^st^ century. Although COVID-19 is a disease with primarily physical implications, the pandemic and the related government-imposed lockdowns have had a profound impact on mental health and psychosocial well-being across the globe. The global populace has been faced with the fear of contracting the virus, the lack of an effective vaccine program, and the potential adverse socio-economic consequences related to unemployment and global decline of economies, and fear of lack of access to essential commodities. These factors have led to a mental health crisis of unprecedented nature, witnessed not only on an individual level but also on the societal level.

A wealth of studies has shown increased burden of mental health problems during the pandemic amongst the general public (please see reviews [1–3]. Studies have reported greater symptoms of depression and anxiety, greater stress, and lower psychological well-being in general public across the globe, compared to pre-pandemic levels [4–8]. Increases in social distress and maladaptive coping styles to manage stress have emerged, with rise in patterns of alcohol and drug abuse and domestic violence cases [9, 10]. These negative effects on mental health have been found to be even more pronounced in essential or system-relevant workers, with poor sleep quality and distress being reported additionally [11–13]. Furthermore, children and adolescents have been particularly vulnerable during this period due to exposure to chronic stress, domestic violence and isolation from peers, which has led to increased levels of loneliness in the youth population [14]. This would further enhance the risk of developing mental health disorders in the long-run. Preliminary evidence indicates that women [15–17], youth populations [18], individuals who report poor sleep quality [12, 16], individuals reporting poor family functioning [15], and individuals employed full-time [19] may be at an increased risk for mental health problems. Furthermore, a range of psychological risk factors have been found to predict worse mental health outcomes during the pandemic, such as worry [19, 20], psychological inflexibility [21, 22], ineffective or maladaptive use of emotion regulation and coping strategies [23–25], lower levels of social support [26], and lack of cognitive control over emotions [27]. Therefore, the pandemic-related lockdowns, although necessary to curb the growth of COVID-19 infection rates, seem to have led to the exacerbation of existing mental health problems.

A parallel line of research has focused on exploring the protective factors that could play a role in insulating individuals from mental health problems during the pandemic. Studies investigating factors of psychological resilience, i.e., the ability to adapt positively and bounce back from adversity [28], during the pandemic found a negative relationship between resilience and mental health problems such as depression, anxiety and somatization [29–31]. Specifically, empirical studies have shown that greater social support, higher levels of mindfulness, cognitive control over emotions, and greater psychological flexibility predict the relationship between illness perceptions, resilience and mental health problems [15, 21, 27, 32]. This indicates that individuals who were able to obtain more social support, and who were able to flexibly adapt their coping strategies in the face of distress, showed the most positive mental health outcomes during the pandemic. Furthermore, empirical studies found social cohesion, measured in terms of sense of belonging, to be associated with mental well-being during lockdowns [33, 34]. Similarly, a recent study found that fear of compassion predicted worse mental health and lower social safeness and closeness, suggesting a protective role of compassion in predicting mental well-being during the pandemic [35]. Similarly, increased prosocial behavior also served as a protective factor mediating the relationship between perception of COVID-19 and mental health symptoms [32]. On the other hand, studies also showed decline in empathic concern and prosocial behavior in adolescents as a result of the lockdown [36]. However, coping strategies aimed at increasing social cohesion, such as engagement with the neighborhood and community, led to reductions in reports of loneliness and induced greater overall well-being [37]. This indicates that protective factors, namely resilience and social cohesion, played an important role in guiding mental health vulnerability and concerns during the pandemic. Since psychological resilience and social cohesion can be improved through evidence-based interventions, programs aimed at enhancing these protective factors in a preventive manner in at-risk individuals would likely lead to improved mental well-being on the global scale. Therefore, in the current study, we aim to examine the efficacy of socio-emotional and mindfulness-based online interventions in improving resilience and social capacities while reducing mental health problems.

In the last decades, mindfulness- and meditation-based psychological intervention programs, inspired by contemplative traditions from the East, have increasingly gained attention. The first most prominent mindfulness programs are the 8-week Mindfulness-Based Stress Reduction (MBSR) program [38] and the Mindfulness-Based Cognitive Therapy program (MBCT; [39] Another prominent class of secular mental training programs are the compassion-based interventions, such as the 8-week Mindful Self-Compassion program (MSC; [40]) and the Compassion-Focused Therapy (CFT; [41]. Increasing evidence for the efficacy of these interventions has shown substantial improvement in mental health [42–45], reduction in stress [42, 46–48], increased immune [49, 50] and emotion-regulation capacities [51, 52], as well as augmented social capacities such as (self-)compassion and prosocial motivation [53–55].

Mindfulness-based programs, such as MBSR, consist of practices that largely focus on bringing non-judgmental awareness to the present moment. These practices require the individual to intentionally focus towards internal (breath, body) and external experiences (sounds, tastes) occurring in the present moment [56]. For example, breathing meditation is a classical form of attention-based mindfulness practice that involves bringing the attentional focus to one’s breath to stabilize the mind and bring awareness in the present moment. Another well-known attention-based meditation involves meditating on sound, where the object of one’s attention is the variety of sounds occurring in the environment. Mindfulness-based interventions have been shown to be helpful in treatment of several mental disorders [57, 58]. A range of interventions have been developed which have shown efficacy in reducing stress and chronic pain [59], alleviating depressive symptoms [39], preventing addiction-related relapses [60], and improving healthy eating behavior [61]. Further, they have also shown to have a positive impact on psychological resilience [62], and enhancing factors of social cohesion such as interpersonal functioning [63]. The adaptation of these mindfulness-based interventions to online platforms have also shown significant, small to moderate effects in alleviating depressive and anxious symptoms, stress reduction, and enhancement of mental well-being [44]. Meanwhile, the compassion-based programs, such as MSC and CFT, have been geared towards enhancing socio-affective competencies, such as self- and other-compassion, loving kindness, prosocial motivation, altruism and empathy, in order to further improve personal and social resilience [64]. These compassion-based programs [40, 41, 65, 66] are a confluence of mindfulness-based exercises and compassion meditation practices, which often forms an integrative structured multi-component program rooted in explicitly cultivating qualities such as acceptance, care, gratitude and compassion. For example, the MSC program involves exercises aimed at developing compassionate inner voice, dealing with difficult emotions and interpersonal relationships, and living in accordance with core values. Compassion-based programs often involve the combination of more formal meditation practices (such as loving kindness meditation), and informal, interpersonal exercises (such as interpersonal sharing of self-criticizing and loving words).

Recently, a large-scale mental training study, the ReSource project [67] extended these 8-week mindfulness- and compassion-based programs to the duration of 9 months, while adding new types of mental practices as well. This longitudinal study included different types of training modules which allowed for the direct comparison of three different training modules which focused on: 1) attention-based mindfulness practices (Presence), 2) compassion-based socio-emotional practices (Affect), and 3) meta-cognitive socio-cognitive practices (Perspective; for a review, see [68]. The Presence module trained attention-based mindfulness, the Affect module focused on socio-emotional skills such as compassion, gratitude, care and coping with difficult emotions through acceptance, and the Perspective module focused on training meta-cognitive and socio-cognitive skills such as perspective-taking on aspects of self and others. The different modules lasted for 3 months each, and consisted of a combination of classic meditation practices performed alone and partner-based interpersonal exercises. For example, the compassion-based Affect Module incorporated a specific daily exercise called the ‘Affect Dyad’, along with regular practice of Loving-Kindness Meditation. These specific daily interpersonal exercises, performed online with another partner, were introduced in the ReSource project in the two social modules, Affect and Perspective. These 10-minute daily “Contemplative Dyads” focused on explicitly fostering social connectedness and social capacities, such as empathic listening and perspective taking on others [69]. For example, the ‘Affect Dyad’ is a daily 10-minute, partner-based socio-emotional exercise, which fosters empathic and non-judgmental listening, acceptance of difficult emotions and cultivation of gratitude, while at the same time increasing social connectedness [69].

The results of the ReSource project (for a review see [68]) revealed that it matters what you practice. While the attention-based mindfulness module, Presence, was most efficient in increasing attention, body awareness and present-moment focus [70, 71] the compassion-based Affect module specifically targeted increases in positive other-related thoughts [70], acceptance, self- and other-related compassion [71–73], and altruism and prosocial behaviors [74]. Similarly, training-related changes in structural brain plasticity revealed differential patterns. While upon undergoing attention-based mindfulness practices (Presence module) grey matter volume increased in attention-relevant brain areas, after socio-emotional training (Affect module) structural plasticity was observed in limbic-and paralimbic networks associated to empathy and compassion [75]. Interestingly, and in contrast to the attention-based mindfulness module, the training modules including the social intersubjective dyad as daily core practices were most efficient in reducing social stress on the hormonal level after a social stressor [76]. Furthermore, the Affect module was found to positively influence the use of adaptive emotion regulation strategies [72]. This indicates that such dyadic socio-emotional interventions may be particularly effective in decreasing social stress and loneliness, while improving social connectedness and socio-affective skills such as compassion and prosocial motivation. This in turn should boost psychological resilience and social cohesion, which are seen as key protective factors for mental well-being.

So far, the rather novel daily dyadic partner-based practice format, introduced in the ReSource project, has only been investigated in the context of participants also practicing other more classical mindfulness meditations together with the dyad practice. Therefore, the singular effects of these dyadic practices are still unknown (with exception of [69]). Furthermore, these intense training programs, where dyadic exercises are combined with other classical mindfulness meditations, last several months. They require considerable time and manpower to execute, with the participants being required to devote practice time in 3-day retreats in addition to several hours working with instructors over the weeks. Therefore, the present study will also serve as a pilot test for whether these more intensive and complex contemplative and mindfulness-based programs can be condensed into low-threshold, shorter, brief interventions that can be scaled to a larger population at low costs in online app-based formats. Furthermore, although there is considerable evidence to support the efficacy of socio-emotional and mindfulness-based interventions on mental health outcomes in regular in-person formats as typically performed in non-pandemic context, there is a dearth of empirical studies focused on investigating their impact as low-threshold online interventions required during a health crisis of global scale such as the COVID-19 crisis. Therefore, in the current study we want to compare the efficacy of short-term online socio-emotional versus mindfulness-based online interventions in enhancing mental health, resilience and social capacities. Specifically, in the present study, we will use some of the attention-based mindfulness practices from the Presence module of the ReSource project as part of our mindfulness-based intervention (e.g., breathing, sound and open presence meditations), and compare these to the efficiency of the daily ‘Affect Dyad’ practice which will form the daily core exercise of the socio-emotional intervention.

Given the impact of such interventions on reduction of mental health problems in other contexts, it can be expected that both types of interventions will lead to positive outcomes on mental health, social cohesion and resilience variables even during a pandemic context. However, given the restrictions on physical contact often imposed by the current pandemic, we intend to test the effects of such interventions in an online format. Such online mental trainings would also allow easier scalability of such mindfulness-based approaches outside of a pandemic context. And indeed, a recent review indicated that digital interventions may be particularly suited to mitigating psychosocial consequences in the current pandemic with requirements for social distancing and limited social contact in place [77]. However, most mindfulness- and compassion-based programs require regular in-person training sessions with teachers, at least to some degree. Although there is some preliminary evidence for the effectiveness of mindfulness-based interventions using online platforms [44], there is a paucity of work examining the efficacy of online dyadic socio-emotional interventions. Such brief dyadic interventions, conducted purely online, might be particularly effective in the current pandemic context and also for future large-scale mental health prevention programs conducted on broader scales. As such, the present study would also importantly serve as a pilot for the future application of brief online dyadic socio-emotional interventions that are effectively applied in online-only environments, requiring no in-person contact with trainers.

### The present study

In the present study, we will test the effectiveness of the daily online ‘Affect Dyad’ practice as the core exercise in the socio-emotional mental training intervention. In the Affect Dyad (AD), participants perform a 12-minute partner-based exercise which involves contemplating over the experience of one difficult situation and one situation which incurred gratitude in the past 24 hours. Both partners take turns speaking about the bodily experience in these two situations while the other partner listens in a non-judgmental and empathic manner. While the participants elaborate on each situation for 2.5 minutes, they are asked to focus on the recall of the bodily sensations and experience of the emotions generated during the situation with the aim to train interoceptive body awareness. The goals of the exercise are to enhance social connectedness, empathic non-judgmental listening, interoceptive body awareness, acceptance of difficult emotions or stress, and the cultivation of care and gratitude.

A second intervention used in the present study will be the daily practice of attention-focused mindfulness-based mental training, which will incorporate attention-based meditations, such as the breathing meditation, meditation on sounds and open presence meditation, as the core exercises. Breathing meditation (BM), for example, is a classical type of attention-based meditation which involves 12-minute individual exercise that requires participants to focus their attention on the sensations of breathing [56]. Participants have to sustain their attention on their breath for long stretches of time, returning their attention to their breath when their mind wanders. The crucial aspect is the training of attention and interoceptive body awareness. Participants will also engage in other similar practices which will focus the attention on external experiences, such as mindfulness on sounds where the object of attention is the sounds in the environment. Finally, the participants will also be practicing open presence meditation with the attention being open to focus on all sensory internal and external stimulations being present [78, 79]. In open presence meditation, instead of concentrating on something, attention is open and remains aware of everything that is happening, with the goal of remaining non-judgmental toward oneself.

The interventions will be administered for a period of 10 weeks in the present study in a parallel group design. Three groups of participants (total *n* = 300) will be created: the first group will undergo the socio-emotional mental training intervention (*n*_*1*_ = 100), the second group will undergo mindfulness-based mental training intervention (*n*_*2*_ = 100), and a final retest control group (waitlist control group; *n*_*3*_ = 100) that will receive no intervention at first and will only be tested on our primary outcomes of interest at pre- and post-test. Then in a second portion of the study, participants from the retest control group (waitlist control) will undergo the socio-emotional mental training intervention in order to ensure the maximum benefit from the study for all participating individuals. Both the other two experimental intervention groups will also be given the possibility to continue their daily assigned practices (respective socio-emotional and mindfulness-based training exercises) after the first post-test for the duration of the 10-weeks during which the waitlist control group undergoes the socio-emotional intervention. A third assessment (post-test 2) will then take place at the end of this second intervention period for the waitlist control group. However, this second post-test of the study will also be offered to the other two groups who underwent the interventions in the first portion of the study, given the number of participants that chose to continue their practice, and availability of time and resources. Please see Fig 3 for the depiction of the study design.

In order to test the effectiveness of these interventions, several self-report and behavioral indicators of mental health, resilience, and social capacities that have been linked to enhanced risk for, and protection from, mental health problems during the pandemic will be examined. The indicators will be examined pre- and post-intervention, and several mechanistic variables of change will be examined during the period of intervention delivery using daily and weekly assessment employing Ecological Momentary Assessment (EMA) approaches. Additionally, the current study will also assess stress hormones (e.g., cortisol), genetic markers and epigenetic markers of stress, vulnerability, and resilience.

#### Stress

We will examine changes in stress before and after the intervention. Mindfulness-based trainings, such as the MBSR program, can significantly reduce stress in healthy individuals [47, 80, 81]. Similarly, socio-emotional trainings have been shown to reduce perceived stress as well as impact hormonal stress markers such as cortisol. In the ReSource project [67], the effects of mindfulness-based and socio-emotional and socio-cognitive trainings (Presence, Affect, Perspective modules) on social stress reduction measured in cortisol levels during the experience of an acute stressor (the Trier Social Stress Task; TSST) were compared, and only the socio-emotional and socio-cognitive training modules which included the daily 10-minutes partner-based dyad exercises showed reliable effects of stress reduction on the hormonal level [68, 76]. Possible underlying mechanisms of all these observed changes are purported to be a social stress buffering effect of learning through the daily dyads with other people to listen and be listened in a non-judgemental manner and to befriend yourself with your own and others vulnerability by practicing acceptance and self-compassion. Further, changes in use of emotion regulation strategies such as acceptance have been proposed [72, 82]. Other possible mechanisms like an increase in interoceptive awareness [83–86], frequency and quality of social interaction [87], common humanity [88, 89], psychological flexibility [90], control over emotions [91], mindfulness [92] and social support [93] as well as a decrease in rumination [94], worry [95] and fear of compassion [96] will be assessed in weekly questionnaires. Other mechanisms that are expected to have an impact on stress reduction through socio-emotional mental training are increased social connectedness and personal disclosure through daily interpersonal practice with another person [69], a decrease in loneliness [97, 98] and changes in current thought patterns [99, 100] and affective states [101]. Those possible mediators will be assessed daily before and after the socio-emotional training intervention to analyze changes and their relation to the expected decrease in stress. In the present study we expect that individuals who undergo the two training interventions will show a decrease in subjectively perceived stress. However, we expect differential changes between the intervention groups in cortisol social stress reactivity after a TSST, and diurnal cortisol stress measured by the cortisol awakening response (CAR) during two consecutive week days in the life of the participants. More specifically, and based on previous research [76], we expect that the socio-emotional Dyad-based intervention as compared to the mindfulness-based intervention will be most efficient in decreasing stress on the hormonal cortisol levels, both for the CAR and acute stress reactivity after a social stressor.

#### Loneliness and social connectedness

Changes in loneliness and social connectedness will be assessed pre-post intervention and during the intervention through an EMA approach. Several studies have found an effect of mindfulness training on loneliness reduction [102–104]. In the context of performing daily ‘Affect Dyads’ in the ReSource project Kok and Singer [69] observed that participants after a given 10-minutes partner exercise indicated that they felt closer to the partner than before. Interestingly, they also showed that subjective connectedness even increased weekly before each Dyad and even if coupled with a new partner still unfamiliar to yourself. Similarly, over the 13 weeks of training of a module, the degree of personal disclosure, that is what participants were willing to share with the other partner, increased steadily. Thus, a sense of shared humanity and connectedness emerged throughout this socio-emotional training practices. Therefore, we will also assess the social connectedness to the respective dyad partner before and after the daily dyad as well as the degree of personal disclosure that was shown during the dyad. We also expect changes in thought patterns [105], affective states [106] and daily loneliness [107] before and after the dyad. Furthermore, we will ask participants on a weekly basis about other variables related to social connectedness, such as the frequency and quality of their social interactions [108], the sense of belonging [109], common humanity [110], fear of compassion [111] and the social support received in stressful situations [112]. We expect that the socio-emotional training will be more efficient than the mindfulness-based intervention in reducing loneliness and increasing social connectedness. We will assess changes in the above postulated mechanistic variables suggested to mediate the reduction in loneliness in the socio-emotional training.

#### Mental health: Depressive and anxious symptoms

Another area of focus for this phase of the study is to assess intervention-related effects on major mental health outcomes. Therefore, we will examine pre-post changes in depressive and anxious symptoms in our sample using Beck Depression Inventory-II (BDI-II; [113]) and State-Trait Anxiety Inventory (STAI; [114]). Previous studies using MBSR and MBCT have shown significant reductions in these symptoms [115, 116]. Similarly, studies employing socioemotional interventions have shown reductions in symptoms of depression and anxiety [43, 117]. Although the mechanisms through which these changes occur are not fully clear, there is preliminary evidence suggesting the key role of changes in emotion regulation strategies used [72, 118, 119]. Therefore, in the present study, we expect an intervention-related decline in depressive and anxious symptoms, compared to retest control group, owing to a change in use of emotion regulation strategies such as increase in acceptance and decrease in rumination [72, 73, 120]. Further, we will also examine the mechanistic role of other factors that are purported to be involved in the onset and maintenance of depressive and anxious symptoms, namely cognitive control over emotions [121, 122], psychological flexibility [123, 124] social support [125], mindfulness [73, 120], and worry [126]. Furthermore, daily fluctuations in affective states and thought patterns will be evaluated as potential mediators of intervention-related changes in depression and anxiety. Daily fluctuations in affective states will be assessed using the ‘Affect Grid’ [127] which measures both the current valence and the affective arousal. Further, we will use ‘Cube of Thoughts’ [100] to assess the intervention-related changes in valence (positive or negative), temporality (past- or future-oriented) and content (self- or other-related) of thoughts. Previous research has linked fluctuations in affect and thought patterns to be associated with depressive and anxious symptoms [128–131]. We will examine changes in mediators using the daily and weekly EMA approaches, employing a variety of validated questionnaires (please see Measures section). Lastly, we will also examine changes in key mechanisms of negative attention bias and negative interpretation bias that underlie the onset and maintenance of depressive and anxious symptoms [132–134]. Both attention bias and interpretation bias will be assessed using the Mouse-Contingent Scrambled Sentences Task [135].

In the current study, we expect both mindfulness-based and socio-emotional interventions to produce significant changes in depression and anxiety symptoms. However, we expect these changes to function through distinct mechanisms, as the type of practice should likely dictate changes in specific mechanistic domains. We expect differential changes in these mediators depending upon the intervention group, which is expected to influence the magnitude of change in depressive and anxious symptoms in the two intervention groups. For example, while both mindfulness-based and compassion-based programs have been shown to reduce the use of rumination as emotion regulation strategy [117], only compassion-based interventions lead to an increase in use of acceptance as emotion regulation strategy [72]. Therefore, we expect differential effects of the different types of interventions on changes in depression and anxiety symptoms.

#### Psychological resilience

We will also examine pre-post changes in psychological resilience in the 3 groups using the Connor-Davidson Resilience Scale (CD-RISC; [136]) and the Brief Resilience Scale (BRS; [137]). In the current study, we conceptualize psychological resilience both as the ability to adapt to stressful situations and also as the ability to bounce back after a stressor [28, 137, 138]. Previous studies have shown that resilience improves upon undergoing mindfulness-based interventions [62]. A key mechanism underlying this change is purported to be psychological flexibility [123, 139]. Further, socio-emotional interventions have also shown success in enhancing stress adaptation, in terms of stress reactivity, in the face of social stressors [76]. However, the effects on the ability to bounce back after a stressor, in terms of the degree of recovery from stress, remain unclear along with the mechanism underlying these changes. Positive coping styles, use of adaptive emotion regulation strategies, and psychological flexibility are purported to be some of the core mechanisms underlying resilience [140]. Further, other mechanisms proposed to be underlying resilience processes will also be investigated, namely cognitive control over emotions [141], self-compassion [142, 143], mindfulness [144], and increase in use of adaptive and decrease in use of maladaptive emotion regulation strategies [145]. Therefore, in the current study we will examine whether the change in coping strategies, such as enhanced social support, greater use of adaptive emotion regulation techniques such as acceptance, decreased use of maladaptive emotion regulations strategies such as rumination and worry, greater mindfulness and self-compassion, greater cognitive control over emotions, and improved psychological flexibility will predict pre-post changes in resilience in our intervention groups, compared to the retest control group. Furthermore, daily fluctuations in affective states and thought patterns will also be evaluated as potential mediators of intervention-related changes in resilience [146]. We will examine changes in mediators using the daily and weekly EMA approaches, employing a variety of validated questionnaires (please see Measures section). In the current study, we expect both mindfulness-based and socio-emotional interventions to produce to significant changes in resilience. However, we will investigate whether different interventions distinctly impact the two components of resilience, namely the ability to adapt to stressful situations and the ability to bounce back. Further, we again expect these changes in resilience to function through distinct mechanisms, as the type of practice will likely dictate changes in specific mechanistic domains. We expect differential changes in use of coping and emotion regulation strategies and changes in psychological flexibility depending upon the intervention group, which is expected to influence the magnitude of change in resilience in the two intervention groups. For example, while both mindfulness-based and compassion-based programs, such as MBCT and CFT, have been shown to enhance mindfulness, the effect sizes are larger for mindfulness-based intervention [117]. However, care and compassion-based socio-emotional intervention will perhaps lead to enhanced use of coping strategies such as acceptance and obtaining social support, which will likely impact distinct components of resilience. Therefore, we expect differential effects of the different types of interventions on changes in resilience.

#### Empathy and compassion

We will also examine pre-post changes in empathy and compassion in the 3 intervention groups. It has long been argued that engaging in mindfulness practice can increase empathic functioning [147–150]. However, previous findings on the effects of mindfulness interventions are somewhat inconsistent. More specifically, they suggest differential effects of different types of practices on affective and cognitive aspects of empathic functioning, and highlight shortcomings in the use of self-report measures of empathy [151]. In the ReSource project, a specific task was developed, the EmpaToM, to test differential effects of different mental training modules on different aspects of socio-emotional and socio-cognitive functioning such as empathy, compassion and Theory of Mind [152]. And indeed, while the socio-affective training module (Affect) was most efficient in improving compassion, the socio-cognitive module (Perspective) was unrivaled in improving Theory of Mind, also called cognitive empathy or cognitive perspective taking [71]. Thus, we will also employ the EmpaToM to assess differential effects of the two different intervention groups on social emotions and cognition. More specifically, based on previous evidence, a training related increase in compassion can be expected specifically for the socio-emotional ‘Affect Dyad’ intervention, whereas none of the interventions should induce any changes in Theory of Mind [71]. This expected increase in compassion does not only apply to compassion for others, but also to self-compassion [153]. With regard to empathy, the predictions are more complex, since previous research has clearly shown differential behavioral, subjective and neuronal patterns and mechanisms underlying empathy and compassion [154–156]. While compassion is based on care and affiliative motivational systems, it is mostly associated with positive affect and warmth, and promotes resilience, empathy, being broadly defined as affect sharing [157]. However, this can also induce negative affect, and even turn into intense feelings of anxiety and dysphoric mood, specifically when a healthy distinction between the self and others is not given [156]. Accordingly, the concept of “empathic distress” is a self-focused component of empathic functioning that indicates a strong and aversive reaction to the suffering of somebody else. Particularly, excessive altruism can lead to empathic distress fatigue, a state of burnout that relates to the regular exposure to others’ suffering [158]. It has previously been found that empathic distress decreases in individuals who undergo MBSR and compassion trainings [89, 159]. It is expected that the compassion-based socio-emotional mental training is thus decreasing empathic distress aspects while increasing positive affect and (self)compassion. At the same time, the socio-emotional dyadic training is aiming at increasing empathic listening and with that the healthy component of empathy. Furthermore, while underlying mechanisms remain largely unknown, differential changes in affect, thought patterns and emotion regulation strategies are also suggested to mediate differential intervention effects on empathy and compassion [160]. Thus, we would expect more positive other-related thoughts and more positive low-arousal affect after socio-emotional training. Besides, augmented interoceptive awareness, i.e., specifically, an increase in using the body for self-regulation, has been associated with mindfulness-based mental trainings such as the Presence module of the ReSource project that require individuals to focus on bodily sensations [83]. Previous research has repeatedly shown a positive association between interoceptive body awareness, alexithymia and empathy, which are mediated through function of the anterior insular cortex [161–163]. Using different interoceptive body awareness and regulation measures in the ReSource project, Bornemann et al. [83–85] could show that different types of mental training can indeed increase interoceptive body awareness over the training of several months, and that this increase in interoceptive body awareness and regulation goes along with a decrease in alexithymia and better emotion understanding (see also [164]). Interestingly, these training-related increases were observed both after mindfulness-based as well as socio-emotional trainings, therefore suggesting that an increase in interoceptive awareness will be a meaningful mediation mechanism in both intervention groups. We will further examine changes in mediators using the daily and weekly EMA approaches, employing a variety of validated questionnaires (please see Measures section).

#### Prosocial behavior

We will also investigate differential impacts of the socio-emotional and mindfulness-based intervention on altruistic prosocial behavior. Based on previous findings [74, 165, 166], we expect that altruistic prosocial behavior will uniquely increase as a result of the care and compassion-based socio-emotional mental training. We will employ the Zurich Prosocial Game (ZPG) [166] and monetary distribution tasks that are suitable to measure altruistic behavior (i.e., Social Value Orientation, Social Discounting questionnaire) to test differential effects of mindfulness-based and socio-emotional mental trainings. Since altruistic prosocial behavior is based on a specific set of motivations that are directed towards caring for others [74, 167, 168], we will investigate whether intervention-related increases in compassion mediate these effects. We further expect that the decrease in empathic distress (see above) and a concomitant increase in positive affect and other-related thought patterns will mediate training-related increases in altruistic prosocial behavior (similar to compassion). Since empathy and compassion can be considered essential for successful and beneficial social interactions [157, 169], and compassion training may be a useful tool in promoting social relationships [170], we will test a mediating role of frequency and quality of social interactions in a training-related effect on prosocial behavior. We will examine changes in mediators using the daily and weekly EMA approaches, employing a variety of validated questionnaires (please see Measures section).

#### Epigenetic markers

We will also examine the impact of intervention-related changes in epigenetic markers associated with mental health, resilience and social capacities from pre- to post-intervention. Changes in psychopathology and resilience as a consequence of stress exposure can also be reflected in terms of changes in epigenome, such that transcription factor activation embedded by gene regulation and expression changes over time [171]. Epigenetic changes affect the gene transcription, not by changing the genome sequence, but by changing the accessibility of transcription regulators (e.g., DNA methylation, histone modifications; [172]. In conditions of chronic stress, glucocorticoids acting as transcription factors play a prominent role in inducing permanent epigenetic changes, which is evident through enrichment of the DNA methylation site of the glucocorticoid response in stress-related epigenetic changes [173]. These environment-dependent epigenetic changes are further influenced by genetic factors, such that genes, environment and epigenetics come together to produce changes [174]. Several studies have shown changes in DNA methylation following interventions for various disorders such as depression and post-traumatic stress disorder [175–177]. Therefore, intervention-related changes in polyepigenetic risk markers will be evaluated pre- to post-intervention in the present study.

#### Inflammation

In addition to neuroendocrine and epigenetic markers of stress during the SARS-CoV-2 pandemic, we will assess a marker of systemic inflammation, i.e., salivary levels of C-reactive protein (CRP), an acute phase protein that signals activation of pro-inflammatory pathways. During acute stress, the brain responds with a robust inflammatory response, both centrally and in the periphery, likely to ensure survival during threat of injury [178, 179]. Psychosocial laboratory stress alone has been shown to induce a potent increase in the secretion of pro-inflammatory cytokines, such as IL-6 and TNF-a, via autonomic nervous system activation and induction of NFkB signaling in immune cells [180, 181]. Glucocorticoids released during stress can exert inhibitory effects on innate immune responses. During chronic stress, however, the glucocorticoid receptor (GR) may become resistant to the regulatory effects of glucocorticoids, likely in part due to ever-expression of FKBP51 protein [182], further promoting pro-inflammatory activation [183, 184]. There are numerous reports documenting increased low-grade systemic inflammation in individuals living under conditions of chronic stress [179] and, especially, loneliness or social isolation [22]. In addition, systemic inflammation is one of the best-replicated findings in children and adults exposed to early adversity [185]. It has been suggested that peripheral cytokines may cross the blood-brain barrier, promoting neuroinflammation and further potentiating stress responses, with ultimate impact on structure and function of neural circuits implicated in behaviors of depression and anxiety [183]. Accordingly, increased levels of systemic inflammation are a cardinal feature of patients with major depression and certain anxiety disorders [186]. Anti-inflammatory medication is considered a viable treatment for affective symptoms in immune-based depression [187]. In addition, psychosocial interventions and meditation may lower systemic inflammation as a final common pathway [188]. Here, we test 1) whether chronic stress related to the SARS-Cov-2 pandemic, as assessed over the course of the pandemic and associated lockdowns, is associated with increased levels of systemic inflammation, i.e., salivary CRP concentrations, 2*)* whether this increase is accentuated in individuals with early life adversity, and 3) whether the dyadic intervention ameliorates systemic inflammation with impact on mental and physical well-being. We will explore associations of inflammation with neuroendocrine and epigenetic indices of stress, symptoms and treatment response, as well as GxE interactions.

**Fig 1:**
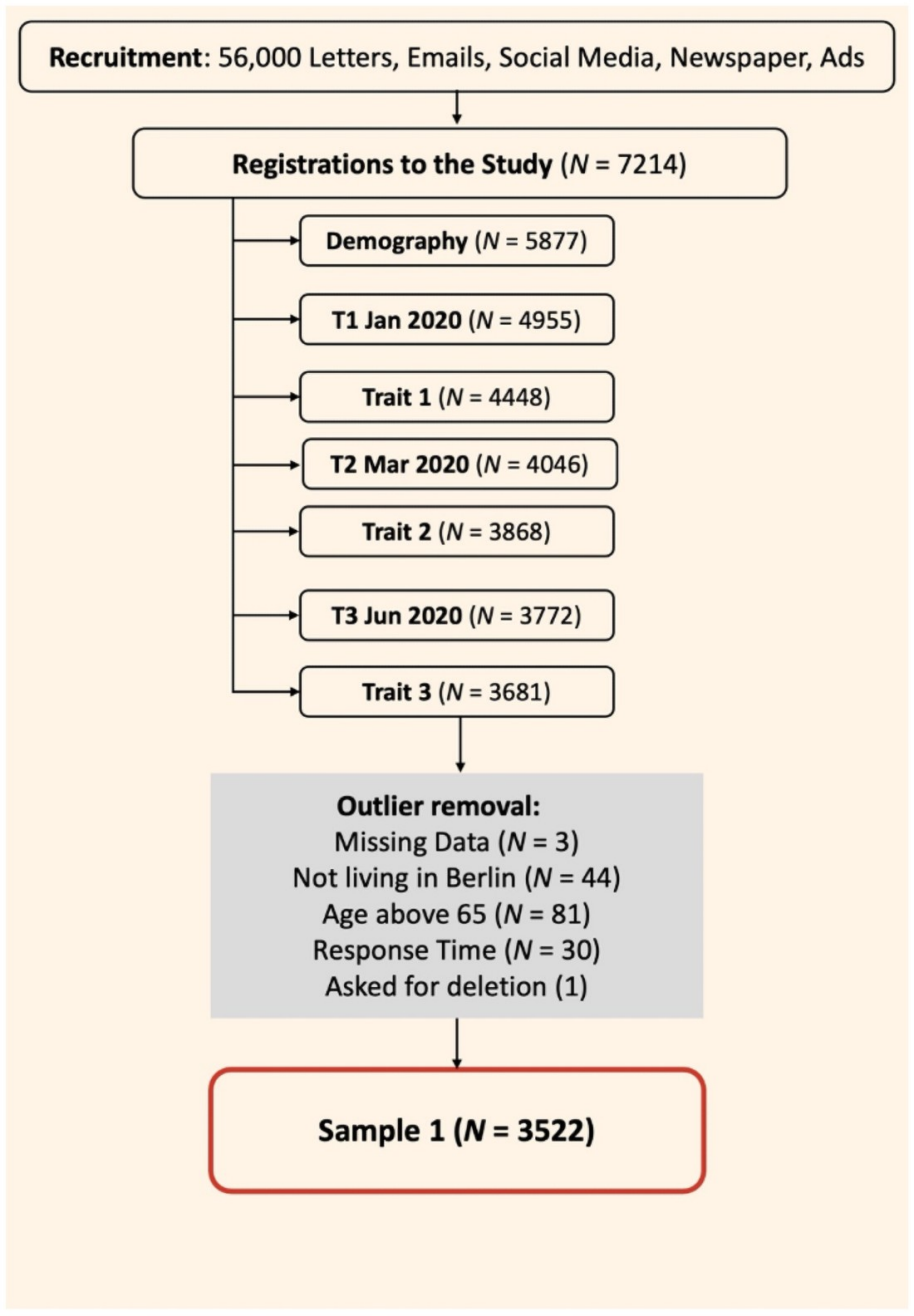
A depiction of the recruited sample, dropouts at every stage of the first phase of the study, and the process of reaching the final sample. Sample 1 indicates the final sample of participants that completed the first three assessment timepoints (T1-T3).

**Fig 2:**
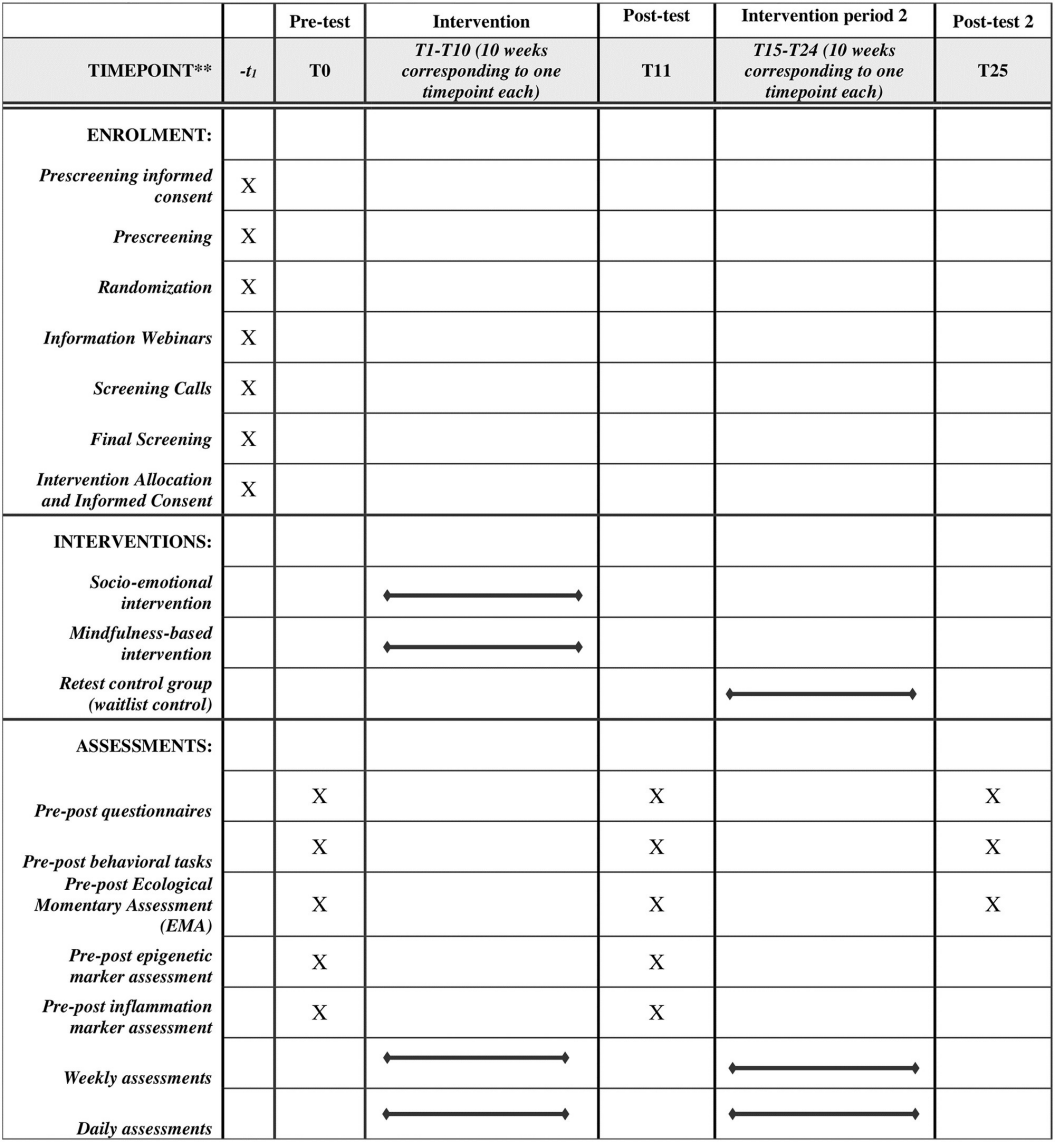
An overview of the schedule of enrollment, interventions, and assessments.

The current study is the second phase of the larger CovSocial project, which previously in its first phase assessed a sample of Berliners on changes in various aspects of mental health, resilience and social cohesion over the course of the pandemic in Berlin, Germany from January 2020 to March 2021 (please see our preregistration for the first phase on Open Science Framework osf.io/jvb98). This allows us to incorporate extensive information about individual longitudinal change patterns in vulnerability, resilience and social cohesion over the period of more than a year during the SARS-CoV-2 pandemic. A broad range of state indicators of those constructs was assessed for 7 timepoints over a period lasting more than a year, from January 2020 to March 2021. In the first phase of the project, participants also provided self-report assessments of personality and behavioral traits (trait) associated with higher vulnerability to stress and mental health problems, resilience and social cohesion. The current study will be conducted in a sub-sample of the participants assessed in phase 1. Please see Fig 1 for a depiction of the sample recruitment for phase 1, detailing dropouts from registration to the completion of the first three timepoints of the first phase of the study. Also, please see Fig 2 for an overview of the schedule of enrolment, interventions, and assessments. Therefore, the longitudinal assessment of mental health, resilience and social cohesion factors from phase 1 of the project, along with the trait measures assessed in phase 1, will also be used to predict both pre-test status and intervention-related changes from pre- to post-test in our variables of interest in phase 2 in an exploratory manner.

Furthermore, during the first phase in December 2020, we assessed genome-wide markers to calculate polygenic risk scores in a sub-sample of participants of phase1, and we will evaluate the predictive impact of these markers on pre-test status and intervention-related changes in our variables of interest during the current second phase (using phase 1 or phase 2 genotype data).

**Fig 3:**
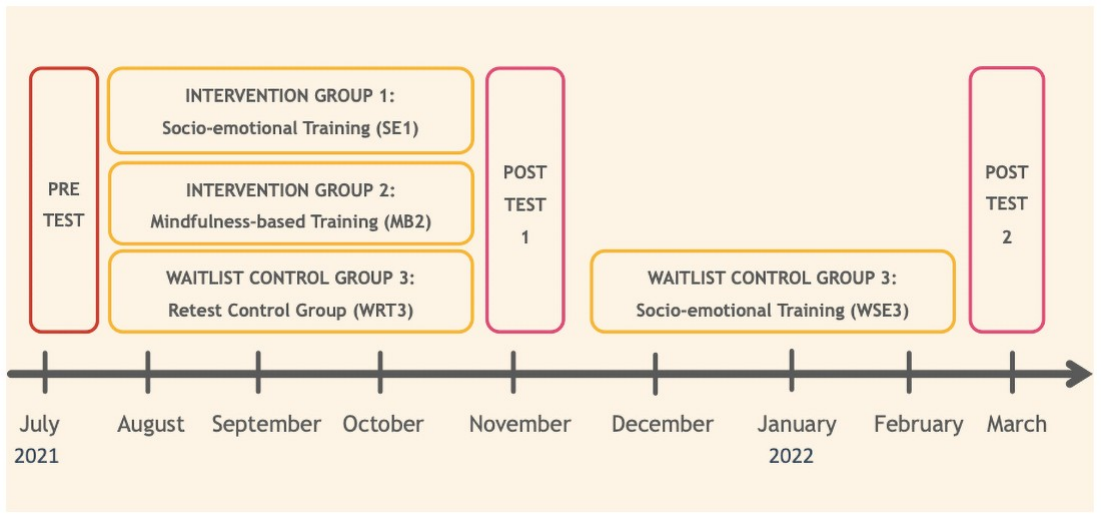
An overview of the study design of phase 2 of the CovSocial project.

Lastly, we will also explore the directional and temporal interplay between the changes in the different mediator variables mentioned in the sections above. Finally, we will also explore the effects of important socio-demographic factors (age, gender, income, education in years, marital status, number of children etc.) and other context variables including COVID-19 related questions (e.g., access to green spaces, job status, covid-related health status, living situation, financial and job risk factors) on pre-test status and the intervention-related changes. For a detailed overview of the hypotheses of the current study, please see our preregistration on the Open Science Framework (osf.io/32c5q). For an overview of the phase 2 of the CovSocial project, please see Fig 3.

## Materials and methods

### Sample and sampling procedure

Participants for the current study will be selected from a community sample of participants from Berlin, Germany that were recruited for the first phase of the CovSocial project (Details of the study site can be obtained from the ClinicalTrials.gov by accessing the study record at https://clinicaltrials.gov/ct2/show/NCT04889508). Participants for the first phase of the project were recruited from the population of the city of Berlin, Germany. The main inclusion criteria involved being between 18 and 65 years of age, being able to understand the German language, and to be registered as a resident of the city of Berlin at the time of the assessment. We used a variety of methods for recruitment including sampling 56,000 individuals from the Berlin registration office, flyers and posters, and advertising on social media, in addition to the chain-referral method. The recruitment led to the registration of 7214 individuals to take part in the study. Out of this, only 5877 started the questionnaires, and at every block of questionnaires we saw further dropout in sample. A total of 3681 individuals completed the questionnaires for the first three timepoints of the first phase of the study. Lastly, participants were excluded for not meeting the inclusion criteria and for data quality reasons, leading to a final sample of 3522 individuals for the first three timepoints of the first phase of the project. Please see S1 Appendix for the number of participants at each further timepoint T4-T7, and all participants who also completed the genome-wide marker assessment.

**Fig 4:**
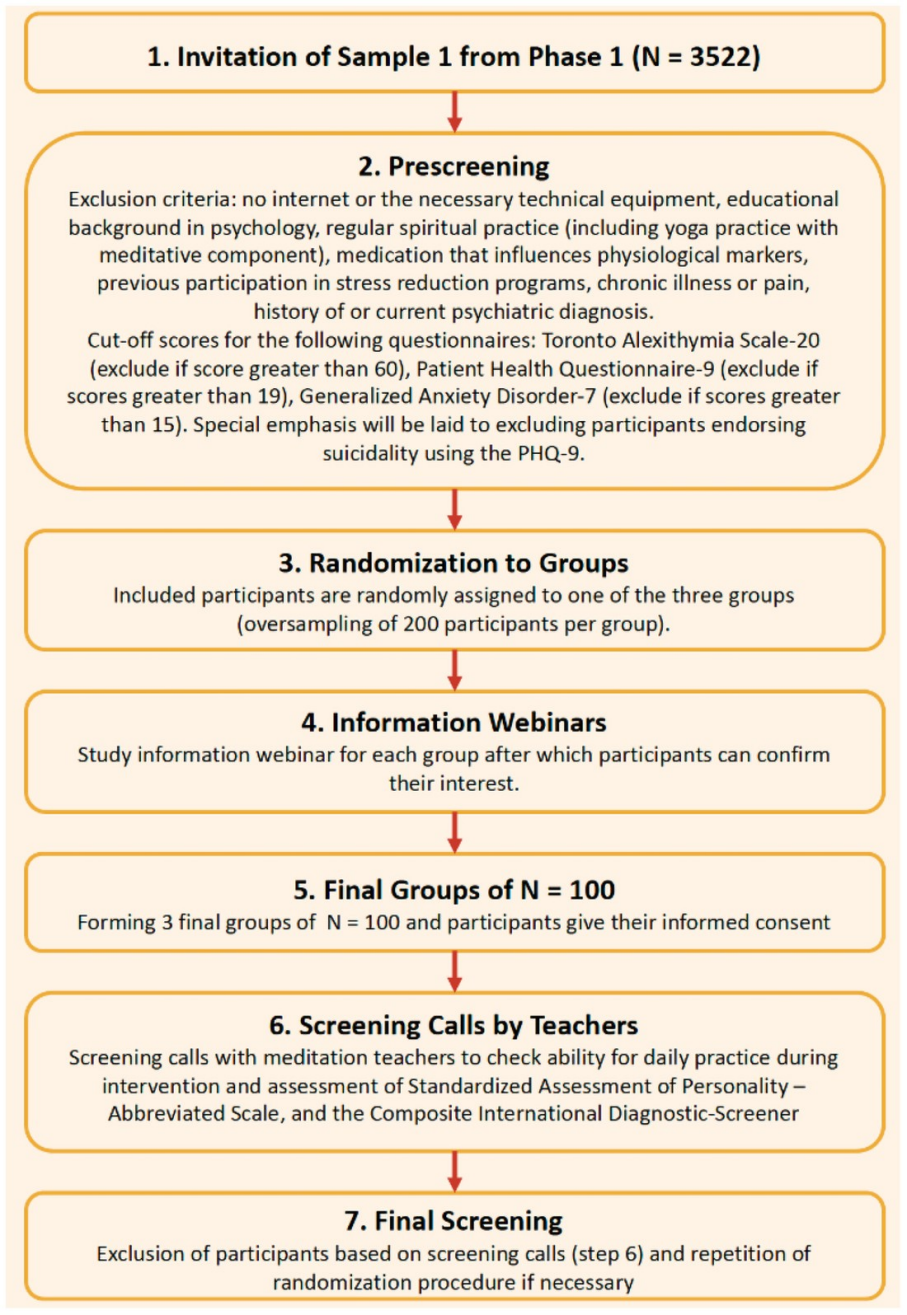
The sampling and randomization procedure.

Participants for the second phase will now be recruited from this initial sample, and assigned to one of the three groups, socio-emotional intervention group, mindfulness-based intervention group, and retest control group (waitlist control), in the following sequential steps (please see Fig 4).

1. First, all participants with completed data sets at the first three timepoints of retrospective longitudinal assessments in phase 1 (T1-T3; *n* = 3522) will be invited to complete a pre-screening to assess whether they qualify to take part in the current study based on the inclusion and exclusion criteria. They will be asked to give informed consent for this pre-screening assessment prior to completing the pre-screening questionnaire.
2. Based on the pre-screening, participants will be selected to take part in the study based on the following inclusion and exclusion criteria.
  - Participants will have to meet the following inclusion criteria: between 18- and 65-years age, resident of Berlin, and proficiency in German language.
  - Participants will be excluded if they do not have access to internet or the necessary technical equipment, if they have an educational background in psychology, if they have regular spiritual practice (including yoga practice with meditative component), if they take medication that influences physiological markers or if they take psychiatric medication, if they have participated in stress reduction programs previously, if they suffer from chronic illness or pain, or if they have a history of or current psychiatric diagnosis. Further, participants will be excluded based on the cut-off scores for the following questionnaires: Toronto Alexithymia Scale-20 (TAS-20; [189, 190]); exclude if score greater than 60), Patient Health Questionnaire-9 (PHQ-9; [190]); exclude if scores greater than 19), and Generalized Anxiety Disorder-7 (GAD-7; [191]); exclude if scores greater than 15). Participants endorsing suicidality on the PHQ-9 will also be excluded.
3. In a third step, individuals who meet all the inclusion and exclusion criteria will be randomly assigned to one of three groups. Initially, the three groups will be oversampled, to ideally form *n* = 200 per group.
4. In the next step all these individuals will be invited to take part in online informational webinars about the study. These online events will serve the purpose of providing interested individuals with information about mindfulness meditation and concepts underlying the socio-emotional mental training. Further, information about exact dates and study procedures will be given. After this event, individuals will have to indicate their interest in participating in the study.
5. In case, the number of interested individuals is higher than the targeted *n* = 100 per group, then the extra number of interested individuals will be excluded based upon study-relevant considerations to make sure that we have three comparable final groups with *n* = 100 each. The participants will then complete the written informed consent indicating their consent to participate in the study.
6. Next, individuals who provide informed consent to take part in the study will undergo screening phone calls with licensed mindfulness-based meditation instructors. In these individual calls, participants will undergo mental health screening using the Standardized Assessment of Severity of Personality Disorder-Abbreviated Scale [192] and the Composite International Diagnostic-Screener [193]. These screening calls would further allow us to exclude individuals who are evaluated as meeting criteria for clinical diagnoses or psychological instability as identified by the meditation instructors, using the screening instruments. This is an important step as the current study is a first step towards exploring the effects of low-intensity online socio-emotional interventions in a non-clinical population. In future studies, this line of research may be extended to clinical populations.
7. astly, if based on the above-mentioned individual screening calls with meditation teachers some individuals have to still be excluded from the study, then the above sampling procedure will be repeated in order to fill any participant gaps in each group.

An estimated 300 individuals, 100 per intervention group, will be recruited. A priori effect size and power calculations have been performed based on the results from the previous mental training study, the ReSource project [67], which developed and validated the interventions being applied in the current study. Power analyses were conducted using G*Power [194] for analysis of variance with repeated measurements and interactions between group and intra-group variables. The analyses elements were as follows: an alpha level of .05, with a power (1-ß) of .80, 3 groups and 2 measurement occasions, a correlation of variables with repeated measurements of .39 which is the lower limit of retest reliability for the Cortisol Awakening Response (CAR; [195]), and an assumed small effect size of *f* = .10. This results in a total sample size of *n* = 297. For the additional third measurement time (post-test 2 following socio-emotional intervention given to waitlist control group) power analysis was conducted in a similar manner as above.

### Study design and setting

We will use a multi-factorial mixed (between- and within-subjects) design. Type of intervention will serve as the between-subjects variable. Type of intervention has three levels: 1) socio-emotional mental training intervention, 2) mindfulness-based mental training intervention, and 3) retest control group (waitlist control). Within-subjects effects, from pre- to post-intervention, will be assessed on dependent variables of interest. Participants in intervention groups 1 and 2 will be assessed on dependent variables at 2 timepoints, pre and post the intervention period (pre-test & post-test). The waitlist control group will be assessed on dependent variables at 3 timepoints, i.e., as retest control group at pre and post the first intervention period (pre-test and post-test) and post a second socio-emotional intervention period, which only applies to this group (post-test 2). Participants will be randomly assigned to one of the three groups in a parallel group design using computer generated numbers in a block randomization technique with 1:1:1 allocation. No blinding will be used since both participants and staff need to be aware which condition the participant belongs to in order to be able to attain and provide the appropriate training and practice prior to starting the actual interventions. The study will be conducted at the Social Neuroscience Lab, Max Planck Society, Berlin, Germany. The study will take place online, with the exception of lab visits for administration of behavioral tasks (2 for groups 1 and 2, 3 for group 3).

#### Ethics approval and consent to participate

The study will be conducted in accordance with the Declaration of Helsinki, and all participants will provide written informed consent prior to their participation in the study. Participation in the study is fully voluntary, and withdrawal from the study is possible at any time. Participants will be reimbursed for the time spent on the testing portion of the study at the rate of 10 euro per hour. The study protocol has been approved by the ethics commission of the Charité –Universitätsmedizin Berlin (EA4/081/21). Modifications of the protocol are reported to the committee. Please see S1–S4 Files for study protocol and the amendment submitted to the ethics commission. The trial is registered at clinicaltrials.gov (NCT04889508). The study is a controlled clinical trial with a retest control condition and will follow the Standard Protocol Items: Recommendations for Interventional Trials (SPIRIT) Statement for reporting trial protocols (see S5 File).

### Measures

**Fig 5:**
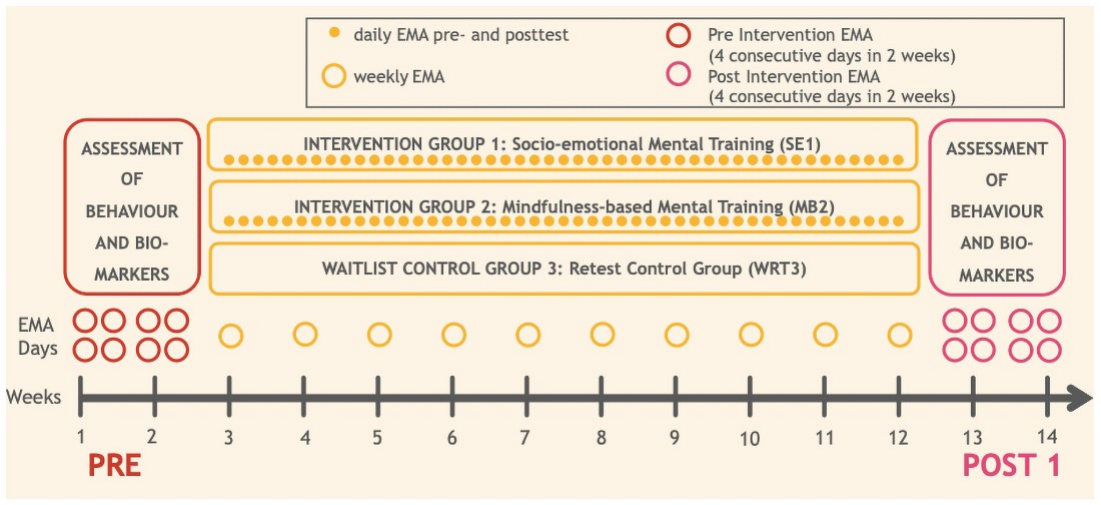
An overview of which measures will be assessed when during the study. Red circles indicate the pre-intervention testing phase, wherein participants will complete the first round of pre-post questionnaires, pre-post behavioral tasks, pre-post EMA and pre-post epigenetic marker assessment. The pink circle indicates the post-intervention testing phase, wherein participants will complete the second round of these measures. The 10 yellow circles indicate the weekly assessments during the intervention. The small solid yellow circles indicate the daily assessments pre and post daily intervention exercise.

Please see Fig 5 for an overview of which measures will be administered when during the study protocol.

#### Pre-post questionnaires

We will assess change from baseline to 10 weeks after the intervention using the following validated questionnaires which will be administered at pre-test, post-test and post-test 2: Perceived Stress Scale-10 (PSS-10; [196]), UCLA Loneliness Scale [197]), Beck Depression Inventory-II (BDI-II; [113]), Connor-Davidson Resilience Scale (CD-RISC; [136]), State-Trait Anxiety Inventory (STAI; [114]), Cognitive Emotion Regulation Questionnaire (CERQ; [198]), Difficulties in Emotion Regulation Scale (DERS; [199]), Brief Resilience Scale (BRS; [137]), Prosocialness Scale for Adults (PSA; [200]) Multidimensional Assessment of Interoceptive Awareness (MAIA; [201]), Fear of Compassion Scale (FoC; [202]), Sussex-Oxford Compassion Scales (SOCS; [203]), State Self-Compassion Scale–Long Form (S-SCS; [204]), Interpersonal Reactivity Index (IRI; [205]) and Toronto Alexithymia Scale (TAS-20; [189]). The following COVID-19 related self-generated questions will also be assessed: belonging to COVID-19 biological risk group, belonging to COVID-19 professional risk group, positive test result for COVID-19, presence of COVID-19 symptoms, hospitalization due to COVID-19 and the duration of hospitalization, the history of or current presence of long-covid symptoms, status of COVID-19 vaccination (received none, first or both doses), perception of benefits of COVID-19 vaccination (in case of one or two doses received), willingness to receive COVID-19 vaccination (in case of no vaccine dose received), and impact of COVID-19 related restrictions and limitations. Participants will also answer questions regarding their smoking behavior, alcohol consumption behavior (using AUDIT-C; [206]), current pregnancy (if any), use of hormonal medication, presence of endocrine disorders, oral contraceptive use, and Body Mass Index (BMI). The questionnaires will be completed using a mobile and web app.

#### Pre-post behavioral tasks

The behavioral tasks will be administered in person in lab. We will assess change from baseline to 10 weeks after the intervention using the following behavioral tasks:

- EmpaToM [152]: This task allows to simultaneously measure empathy, compassion and theory of mind (ToM). It consists of 48 emotionally neutral or negative short video sequences. Valence (empathy) and feelings of compassion (compassion) will be rated by participants, followed by multiple choice questions that demand a ToM-inference or factual reasoning.
- Zurich Prosocial Game [166]: This game serves as implicit assessment of prosocial behavior in the context of reciprocity, helping cost and distress cues. Measure of interest is the number of keys that a participant invests in removing obstacles from another player’s maze path when no reciprocity is given, independent from reciprocity (percentage of times the other player removed obstacles of the participant’s maze) and helping cost (percentage of times when helping costs keys left for one’s own maze). This altruistic prosocial behavior is assessed in two conditions, i.e., with and without distress cues.
- Social Value Orientation [207]: Participants are asked to allocate money between themselves and another person. Thereby, participants choose between three distribution options (prosocial = optimizing other’s gain; individualistic = optimizing one’s own gain; competitive = maximizing the difference in gains). The task will be conducted in 9 rounds. Number of prosocial choices will be the measure of interest.
- Social Discounting [208]: For each of 7 imagined acquaintances of different social distances to them, participants will distribute money to either themselves or another person and themselves in 9 different settings. Distribution choices can be either selfish or generous. The crossover point between last selfish and first altruistic choice represents the amount they are willing to forgo for another person. The degree of discounting (k) under the assumption of a hyperbolic function between social distance and money distributed to others will serve as variable of interest.
- Scrambled Sentences Task (SST; [135]): Scrambled sentences task assesses emotional biases in the individual’s tendency to interpret ambiguous information. We will employ the mouse-contingent version of the scrambled sentences task [135]. In this task, participants unscramble sentences using five of the six displayed words to form grammatically correct and meaningful statements (e.g., looks the future bright very dismal). By reporting the unscrambled sentence that first comes to mind, every sentence is resolved in either a positive (e.g., the future looks very bright) or negative (e.g., the future looks very dismal) manner. Twenty unscrambled sentences designed to tap into depression-relevant themes and 20 designed to tap into anxiety-related themes will be presented. An index of negative interpretation bias will be obtained from the ratio of negatively unscrambled sentences to the total correctly completed emotional sentences. The index of positive interpretation bias will be obtained from the ratio of positively unscrambled sentences to the total correctly unscrambled emotional sentences. Additionally, the mouse-contingent version of the task will also allow us to obtain indices of positive and negative attention bias through tracking of duration of time spent on negative versus positive words. Employing the mouse-contingent version of SST will allow us to assess attention biases for emotional stimuli in a reliable manner, and without additional burden for participants in the form an additional dot probe task to measure attention biases.

#### Pre-post ecological momentary assessment (EMA)

We will assess change from baseline to 10 weeks after the intervention in CAR using saliva samples obtained over a period of 2 days in a week at pre-test and post-test (please see Table 1). On two consecutive days (Thursday and Friday) of a given week, each day participants will provide saliva samples four times a day at the following timepoints: immediately after awakening, +30 minutes after awakening, +60 minutes after awakening, and once in the evening. Furthermore, on 8 days over a period of 2 weeks, 4 days (Thursday to Sunday) each in a week (including 2 days of the saliva samples), participants will receive 5 push notifications randomly jittered over the day on their mobile App to respond to EMA questions about stress assessment and stressor appraisal questions [209], coping or regulation strategy used if a stressor was present (using CERQ and Brief-COPE [210], affective state, valence and temporal orientation of thoughts, loneliness, perceived emotional control and interoceptive awareness. Additionally, for the cortisol samples, participants will be asked directly after the first sample after awakening about sleep quality, time of saliva assessment, hormonal cycle, current illness, work shift and work day/weekend in order to control for these variables. The pre-post EMA will take place at the participants’ location.

**Table 1:** An overview of all the different ecological momentary assessment (EMA) measures in the study, and the time and method of assessment.

Measured Variable	EMA Timepoint in the study	Assessment time	Assessment method	EMA Type
Diurnal Cortisol Profile	Pre- and Post-test	2 consecutive weekdays	immediately after awakening, +30 minutes after awakening, +60 minutes after awakening, in the evening	Event- and time sampling
Quality of sleep, stress assessment and stressor appraisal questions, CERQ, Brief-COPE, Affect Grid (affective state), emotional control, interoceptive awareness, Cube of Thoughts (valence, temporal and social orientation of thoughts), feelings of loneliness	Pre- and Post-test	Over 8 days in a period of 2 weeks, 4 days each in a week (Thursday-Sunday)	5 timepoints randomly jittered across the day	Time sampling
Time of Saliva Assessment, Hormonal Cycle, Current Illness, Jetlag, Shift Work and Work day/Weekend	Pre- and Post-test	On 2 days of cortisol sampling	After the first cortisol sample when awakening	Event Sampling
CERQ, Perceived Stress Scale– 4-item version (PSS-4), FoC Scale (only following subscales will be assessed: Fear of Expressing Compassion for Others and Fear of Expressing Kindness and Compassion Towards Oneself), S-SCS (only following subscales will be assessed: Self-Kindness and Common Humanity), Mindfulness scale (CAMS-R or FFMQ), MAIA (only following subscales will be assessed: Self-Regulation and Body Listening), IRI (only subscale Personal Distress will be assessed), Penn State Worry Questionnaire-3, Cognitive Control and Flexibility Questionnaire (CCFQ), Acceptance and Action Questionnaire (AAQ-II), Brief COPE, Inclusion of Other in the Self Scale (IOS Scale; (216), stress assessment and stressor appraisal questions (209), and three self-generated questions on frequency and quality of social interaction and empathic listening. (“How often did you have social contact last week?”, “How pleasant were these social contacts on average?”, “How well were you able to listen to the other person in this interaction?”)	Weekly assessment	Once a week, over the period of 10 weeks of intervention	Once a day	Time sampling
Affect Grid, Cube of Thoughts, feeling of connectedness to partner, level of interoception (awareness of bodily sensations), loneliness, feeling of engagement with the exercise (only post), and personal disclosure (only post Affect Dyad)	Pre and Post daily intervention exercise	Daily (feeling of engagement with the exercise), and every other day (all other variables) over the period of 10 weeks of intervention	Twice daily (6 times per week)	Event sampling

#### Pre-post epigenetic marker assessment

We will assess change from baseline to 10 weeks after the intervention in the polyepigenetic scores. Participants will provide an additional saliva sample (Oragene-DNA, DNA Genotek) during the same time as when they complete the pre-post EMA saliva samples mentioned above. DNA will be extracted from these saliva samples and DNA methylation will be measured using Illumina EPIC DNA methylation arrays and genome-wide SNP genotyping using Illumina Genome Screening arrays will be performed in all individuals not genotyped in Phase 1.

#### Pre-post inflammation marker assessment

We will assess change from baseline to 10 weeks after the intervention in CRP levels. Participants will provide an additional saliva sample during the same time as pre-test and post-test EMA assessment and epigenetic marker assessment mentioned above. CRP levels in the saliva will be extracted as markers of inflammation.

#### Weekly assessments during intervention

We will assess weekly changes in mechanistic variables of interest over the period of 10 weeks of intervention using the mobile or web app designed for the study. Participants will once a week complete CERQ, Perceived Stress Scale– 4-item version (PSS-4), FoC Scale (only following subscales will be assessed: Fear of Expressing Compassion for Others and Fear of Expressing Kindness and Compassion Towards Oneself), S-SCS (only following subscales will be assessed: Self-Kindness and Common Humanity), Mindfulness scale (CAMS-R [211] or FFMQ [212]), MAIA (only following subscales will be assessed: Self-Regulation and Body Listening), IRI (only subscale Personal Distress will be assessed), Penn State Worry Questionnaire-3 [213], Cognitive Control and Flexibility Questionnaire (CCFQ; [214]), Acceptance and Action Questionnaire (AAQ-II; [215]), Brief COPE, Inclusion of Other in the Self Scale (IOS Scale; [216]), stress assessment and stressor appraisal questions [209], and three self-generated questions on frequency and quality of social interaction and empathic listening.

#### Daily assessment pre and post daily intervention exercise

We will assess daily changes in mechanistic variables of interest over the period of 10 weeks of intervention using the mobile or web app designed for the study. Participants will be asked to evaluate their feeling of engagement with the exercise on a daily basis after the training session. Questions pre and post daily exercise sessions that will be presented alternately every other day include affective state (Affect Grid), valence, temporality and social orientation of thoughts (Cube of Thoughts), feeling of connectedness to partner (IOS), level of interoception (awareness of bodily sensations), and perceived loneliness. Level of personal detail shared (personal disclosure) will only be assessed after the Affect Dyad exercise in the socio-emotional intervention.

#### Phase 1 measures

Data on various measures of trait and state vulnerability, resilience, and social cohesion will also be obtained from the recently completed first phase of the CovSocial project (osf.io/jvb98). The data will be used to test hypotheses that predict impact of phase 1 trait and longitudinal assessments on intervention-related change in phase 2.

### Interventions

#### Socio-emotional mental training (group 1)

The behavioral intervention will consist of 10 weeks of daily Affect Dyad practice with a partner as the core exercise. In the Affect Dyad (AD), participants perform a 12-minute partner-based exercise which involves contemplating over the experience of one difficult situation and one situation which incurred gratitude in the past 24 hours. Both partners take turns speaking about the two situations while the other partner listens in a non-judgmental manner. While the participants elaborate on the situations, they are asked to mostly focus on the bodily experience of the emotions generated during the situation. Daily practice of the dyad begins with a moment of silence wherein participants are required to center themselves and let go of the events and feelings experienced during the day thus far. After this, the first partner speaks about a difficult or stressful situation they experienced during the past 24 hours, and the emotions arising in that situation were felt in their body. While the first partner speaks, the other partner remains silent and is instructed to listen in an empathic non-judgmental manner. After speaking for 2.5 minutes about the difficult situation, the first partner then speaks about a situation that made them feel grateful or gratitude experienced in the past 24 hours, and how the emotions arising during this situation were felt in their bodily sensations. The partner once again remains silent and listens with an empathic and non-judgmental stance. Once the first partner finishes speaking about the grateful situation for 2.5 minutes, the entire procedure is then repeated with the second partner as speaker and the first partner as listener. The exercise ends with a moment of silence once again. Participants perform this daily exercise 6 times a week. Prior to each week of daily dyad practice, the participants are randomly paired to one other participant in the Affect Dyad group. The goal of the exercise is to enhance coping with difficult emotions, increase social connectedness, (self) acceptance, empathic and non-judgmental listening and gratitude.

#### Mindfulness-based mental training (group 2)

The behavioral intervention will consist of 10 weeks of daily individual attention-based mindfulness meditation practice. One of the core practices will be Breathing Meditation (BM), a 12-minute individual exercise that requires participants to focus their attention on the sensations of breathing. Participants have to sustain their attention to breath for long stretches of time, and have to return their attention to their breath when their mind wanders. Participants will also engage in other practices, such as attention-based mindfulness on sounds (object of attention is sounds in the environment) and open presence meditation (object of attention is sensations present in the inner and outer environment). Daily practice of the meditation takes place through pre-recorded exercise audios recorded by meditation trainers who guide the participants through the entire 12-minute exercise. The exercise begins with participants being asked to sit in a comfortable position that makes them relaxed yet keeps them awake and aware. They are asked to feel the contact to all the objects that their body rests on, and are asked to cultivate an attitude of dignity and receptivity towards themselves and their body. The exercise encourages them to focus their attention on their breath, and to bring back their attention to the breath when their mind has become distracted or has wandered. The key focus of these practices is on training present-moment attention and interoceptive body awareness.

#### Retest control group (waitlist control group 3)

The retest control group, which is also a waitlist control group, will only be tested prior to and after the 10-week period at pre-test and post-test wherein other groups undergo the interventions. In a second step, the waitlist control group will then also undergo a 10-week period of socio-emotional intervention, with the exact same protocol as the socio-emotional intervention experimental group 1 described above.

### Instructor training

The interventions will be conducted using an online mobile and web app. However, participants will also be assigned to one of four MBSR-trained teachers. These instructors will introduce participants to the interventions, coach them on the basics of the interventions, and will conduct “weekly check-ins” with participants. All MBSR instructors have received a comprehensive in-house training, which includes multiple workshops by leading experts to ensure high qualification in their ability to manage participant adherence to the interventions.

### Procedure

The study will begin with the invitation of Berliners who took part in the first phase of the CovSocial project. The invitation for the current study will include brief information about the current second phase of the project, and the link to complete the online pre-screening questionnaire. Once interested individuals complete the prescreening, the results from the questionnaire will be analyzed to select the appropriate individuals who meet the inclusion and exclusion criteria (listed in the sample section above). The selected individuals will be randomly assigned to form three tentative groups with *n* = 200 each, socio-emotional intervention group, mindfulness-based intervention group, and retest control group (waitlist control). These selected individuals will then be invited to an online information webinar for further detailed information about the study, including introduction to the specific intervention they have been randomly assigned. Upon the webinars, individuals who express interest in taking part in the study will receive an email to formally sign up for the study by providing formal informed consent, in order to form final three *n* = 100 groups. The participants will then undergo short 30-minute screening calls with MBSR instructors, who will ultimately also track the progress of participants throughout the course of the intervention with weekly check-in meetings. After the screening calls, a final assessment of the three groups will take place, and further participants will be recruited in case of dropouts to make sure that we have *n* = 100 per group. This will complete the recruitment procedure which will take place entirely online.

After the recruitment, participants will complete a 2-week pre-intervention EMA, wherein they will provide saliva samples at specific timepoints and answer questions about their mental and physical health during those corresponding timepoints. This period of data collection will take place at the convenience of participants (at their home, workplace, etc.) to be able to capture the ecologically valid CAR and stress response. For this portion, participants will be sent the appropriate saliva collection kits by mail. Next, all participants will then be invited to the lab to complete the pre-intervention assessment of the behavioral tasks, and they will also complete the pre-intervention assessment of questionnaires using the web and mobile App. This pre-intervention assessment period will last approximately 4 weeks, after which the intervention period will begin.

The intervention period will last 10 weeks, and no blinding will be employed During each week participants in the two intervention groups will perform the intervention-specific exercises 6 times a week using the mobile or web app designed for the study. Every day prior to and after completing the intervention exercise, participants will complete the intervention-related daily EMA using app. Once a week, participants will undergo 2-hour online coaching sessions with an intervention instructor. The purpose of these so-called “weekly check-ins” would be to ensure that participants are able to share insights gained during the week, resolve issues faced during the week, and to keep up the motivation and commitment of participants. Once a week, at a random timepoint, participants will also complete the weekly assessment measures tracking changes in various potential mechanistic variables of interest using the app. During this first intervention period, participants in the retest control group will not complete any daily EMA or weekly assessments. After the completion of the first 10-week intervention period, all participants will again be invited to the lab to complete the same battery of behavioral tasks at post-intervention, and will also complete the same battery of questionnaires through the web and mobile App. Participants will once again undergo a 2-week post-intervention EMA period, during which they will provide saliva samples at specific timepoints, along with answering questions about mental and physical health at those timepoints.

Upon the completion of the post-test, all participants in the retest control group (waitlist control) will then undergo the 10-week socio-emotional intervention period. Same as above, during each week participants in the waitlist control group will perform the intervention-specific exercise 6 times a week. Every day prior to and after completing the intervention exercise, they will complete the intervention-related daily EMA, and once a week, they will undergo 2-hour coaching sessions with an intervention instructor. They will also complete the weekly assessment measures, once a week at a random timepoint, to track changes in various potential mechanistic variables of interest. The participants will then undergo post-test 2, wherein they will complete the behavioral tasks, pre-post questionnaires, and pre-post EMA. Lastly, post-test 2 will also be offered to participants in the other 2 groups depending upon the availability of resources.

Pre-post questionnaires will be assessed using an online survey which will be implemented in a research WebApp (app.covsocial.de). This WebApp has been developed for the purpose of the CovSocial project. The WebApp and linked Webpage (www.covsocial.de) are developed by the web agency CosmoCode GmbH. Weekly assessments during intervention and daily assessment pre and post daily intervention exercise will also take place through the app.

### Data management and monitoring

Data collection and management will adhere to German law. Participant data will be stored on encrypted institute servers in a pseudonymized manner. This will allow only the dedicated study personnel to access the full details of participants (i.e., personal identifying and study data). Since participants have the right to withdraw from the study at any time, provisions are in place to delete all individual data from servers if requested by a participant. Data will be presented in eventual publications, and will be fully anonymous. This anonymized dataset will be accessible to all investigators/authors of the protocol. Study documents will be kept at the Social Neuroscience Lab, Max Planck Society for the duration of the study and consecutive data analysis. Biological data will be stored in biobank. Since the current trial does not involve blinding, the establishment of a data monitoring committee is not necessary. The occurrence of adverse events will lead to the immediate exclusion of the participant from the study. These serious adverse events will be reported to the ethics commission of the Charité –Universitätsmedizin Berlin.

### Trial Status

This is the first version of the protocol. The first pre-screened participant was invited to be informed about the study on May 27^th^, 2021. The recruitment phase or the data collection phase of the trial will be completed by the end of March 2022 including post-test and post-test 2 measurements.

### Statistical analysis plan

Statistical Models will be conceptualized and set up within the structural equation modeling and the mixed-effects modeling frameworks and estimated with corresponding packages from the statistical analysis software R. Each hypothesis will be tested for statistical significance using α = .05.

#### Effects of mental training interventions on pre-post changes in variables of interest

In order to evaluate intervention-related effects on variables of interest, a mixed-effects modeling approach will be applied. Effects of time of assessment (pre-test/post-test for primary outcome variables and pre-training session/post-training session for secondary outcome variables respectively) and intervention group (socio-emotional mental training/mindfulness-based mental training/waitlist control group), as well as the interaction between time and group will be defined as fixed effects. Dummy coding with the waitlist control group as reference will be used for the intervention group variable. Additionally, we will control for participant-related random effects.

**Fig 6:**
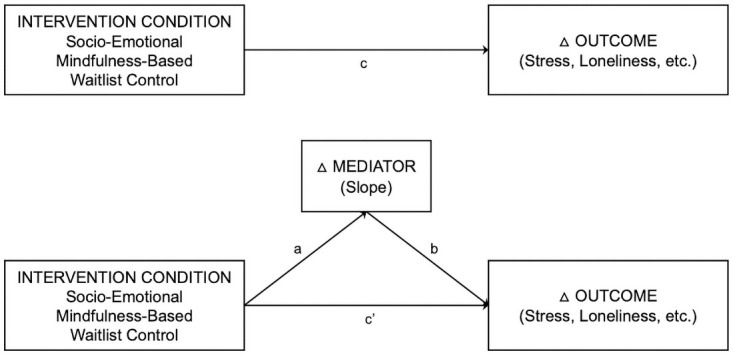
Graphical representation of the hypothesized mental training effects. △ = change.

In addition, latent change scores for each primary outcome variable respectively will be computed with two timepoints (pre-test/post-test). The change in outcomes will be modeled as a latent variable and this latent change will be predicted by the treatment condition (path c in Fig 6). A correlation between the latent change score and initial levels on the primary outcome variable of interest is evaluated.

#### Effects of mental training interventions on changes in mediator variables over time during intervention period

We will employ latent growth curve modelling to firstly test changes in daily (pre- and post-daily exercise sessions respectively) and weekly mediator variables over time (unconditional models) and secondly to test the effects of mental training interventions on changes in mediator variables over time (conditional models). Depending on the model fit, latent slope factors will successively be modeled with a linear, quadratic, or freely estimated (only first two factor loadings of growth are constrained) growth over time during the intervention period. Latent intercepts will be modeled with factor loadings at every timepoint constrained to 1. In conditional models, slopes are regressed on group variables (dummy coded for socio-emotional mental training/mindfulness-based mental training) (path a in Fig 6). We will also explore the directional and temporal interplay between the changes in the different mediator variables using different SEM approaches to model daily, weekly and pre-post mediators.

#### Associations between changes in outcome variables and changes in mediator variables

In order to evaluate an association between changes in outcome variables and changes in mediator variables over time, latent change scores of primary outcomes and latent slopes of mediator variables will be tested for their correlation. To account for partial mediation, direct paths of latent slopes of mediator variables to latent change scores of primary outcome variables will be estimated (path b in Fig 6).

#### Mediation analysis

The direct effect of the treatment condition on the outcomes of interest (path c’) will be estimated in the SEM framework. The amount of mediation will be indicated by comparison of the direct effect when controlled for the mediator variable (path c’) and the direct effect without controlling for the mediator (path c).

#### Prediction of pretest and intervention-related changes in variables of interest by trait-level individual characteristics and changes in state-levels of vulnerability, resilience and social cohesion

Further, we are interested in exploring whether certain individuals respond better or worse to the interventions based upon specific trait characteristics as well as trajectories of state-level psychological vulnerability, resilience and social cohesion during the course of the pandemic in phase1 (T1-T7). For this, data from phase 1 of the CovSocial project will be used. Phase 1 data has been previously analyzed using a latent factor model approach for trait vulnerability, resilience and social cohesion, and latent change score models for changes in state vulnerability, resilience and social cohesion. Standardized latent factor scores obtained from the trait and state factor models constructed and published in phase 1 (Silveira et al., in preparation) will be used in our current mixed effects modeling approach. Please see preregistration of phase 1 for further details on what indicators constitute the trait and state vulnerability, resilience and social cohesion factors (osf.io/jvb98).

1. We will examine whether trait predispositions predict mental state at pretest and moderate pre-post intervention changes in the current phase 2 of the project (‘trait effect’). Latent change factors will be employed as outlined above. Observed primary outcome variables at pretest as well as latent changes of those outcome variables will be regressed on the latent trait factors.
2. Next, we will evaluate whether state changes in stress and vulnerability, resilience and social cohesion during the pandemic (T1-T7) can predict state variables of interest at pretest and intervention-related changes in phase 2. Four different models of latent change will be computed using phase 1 data:

- “Acute first lockdown effect”: The change in latent factors from pre-lockdown to the first lockdown will be determined using latent change score models (T1-T2 in phase 1)
- “Recovery effect”: The change in latent factors from the first lockdown to re-opening in Berlin will be determined using latent change score models (T2-T3)
- “Second lockdown fatigue effect”: The trajectory of change in latent factors during the second lockdown in Berlin will be indicated by the latent slope in latent growth curve models (T4-T7)
- “Cumulative pandemic effect”: We will compute an unweighted sum of standardized latent change score “acute first lockdown effect” and the standardized latent slope “second lockdown fatigue effect”

Observed primary outcome variables at pretest as well as latent change scores of those outcome variables will be regressed on these latent change scores (acute first lockdown effect, recovery effect), latent slopes (second lockdown fatigue effect), and unweighted sum of the change trajectories (cumulative pandemic effect).

#### Background, context and socio-demographic variables

Where necessary, we will account for socio-demographic variables (such as age, gender, marital status, number of children, education in years, and household income) and other context variables including COVID-19 related questions (e.g., access to green spaces, job status, covid-related health status, living situation, financial and job risk factors etc.) assessed in the context of the extensive assessment in phase 1. Predictive value of relevant context- and demographic variables on differences in variables of interest at pretest and their training-related change will be explored similarly to the prediction by latent trait factors assessed in phase 1. Besides, initial levels on outcome variables of interest at pretest will be controlled for by including autoregressive paths in respective models.

## Discussion

The current study aims to examine the impact of online socio-emotional and mindfulness-based interventions on stress, loneliness, social connectedness, mental health, psychological resilience, and social capacities and cohesion in the context of the current SARS-CoV-2 pandemic. Several empirical studies have provided strong evidence for poor mental well-being outcomes owing to the very high levels of stress and isolation generated as a result of the pandemic and the related lockdowns. The massive social changes induced due to measures such as physical distancing and closure of arenas of social participation, have not only impacted individual levels of stress and vulnerability, but also ushered a change in crucial societal foundation of social cohesion. Therefore, the current study will assess whether brief online interventions aimed at enhancing mental well-being, psychological resilience and social cohesion can reduce individual stress, loneliness and vulnerability and enhance individual psychological resilience, and also improve social capacities and cohesion amongst the various elements of society.

The current study adapts socio-emotional and mindfulness-based interventions applied in the ReSource project (67). The project applied intensive, 3-month long, in-person practice of mindfulness-based and socio-affective interventions that led to enhancement of mental well-being, social capacities such as compassion, empathy and prosocial behaviors, and reductions in a range of vulnerability factors such as stress or negative affect (68). In the current study, we plan to test whether such interventions can be beneficial and have measurable effects when delivered in a less intense, purely app-based online format, with only 12 minutes of practice per day over only a few weeks. This could allow for the development of more flexible forms of such interventions to be able to apply these on a larger scale to heterogeneous population samples. As such, we will validate whether brief, daily 12-minute online interventions that last 10 weeks can lead to prevention of maladaptive outcomes such as depression, anxiety, loneliness and stress, and lead to enhanced resilience, mental well-being as well as increased social competencies and cohesion. This will allow us to ascertain whether large-scale application of such interventions, that require minimal time and effort from participants, is feasible or not in terms of application and outcomes. The findings from the present study will also allow us to test whether such brief interventions can be applied to more clinical settings to reduce incidence of development of psychological disorders, such as major depression and generalized anxiety, in the face of more global stressors. Also, two associated questions of interest that will be answered are a) which type of mental training intervention is specifically effective in bringing about changes in which outcomes, and b) whether specific risk groups benefit more from one specific type of mindfulness-based or socio-emotional intervention. Moreover, the study will also clarify whether the biological embedding of stressful experiences, in the form of genotypic and polygenic risk markers of vulnerability, resilience and social cohesion, can be changed or reversed upon undergoing mindfulness-based and socio-emotional interventions. Lastly, the present study will also serve to advance the field of digitalized and personalized mental health interventions, which is increasingly being seen as an alternative to more traditional forms of in-person psychotherapy and interventions.

## Supporting information

S1 File The original study protocol in German submitted to the ethics commission. (DOCX)

S2 File The English translation of the original study protocol submitted to the ethics commission. (DOCX)

S3 File The amendment to the study protocol in German submitted to the ethics commission. (DOCX)

S4 File The English translation of the amendment to the study protocol submitted to the ethics commission. (DOCX)

S5 File The SPIRIT checklist. (DOC)

S1 Appendix (DOCX)

## Abbreviations

AAQ-II: Acceptance and Action Questionnaire-II
AD: Affect Dyad
AUDIT: Alcohol Use Disorder Identification Test
BDI-II: Beck Depression Inventory-II
BM: Berthing Meditation
BRS: Brief Resilience Scale
CAR: Cortisol Awakening Response
CCFQ: Cognitive Control and Flexibility Questionnaire
CD-RISC: Connor Davidson Resilience Scale
CERQ: Cognitive Emotion Regulation Questionnaire
CFT: Compassion-Focused Therapy
CRP: C-Reactive Protein
DERS: Difficulties in Emotion Regulation Questionnaire
EMA: Ecological Momentary Assessment
FFMQ: Five Facet Mindfulness Questionnaire
FoC: Fear of Compassion Scale
GAD: Generalized Anxiety Disorder Scale
GR: Glucocorticoid Receptor
IOS: Inclusion of Other in Self scale
IRI: Interpersonal Reactivity Index
MAIA: Multidimensional Assessment of Interoceptive Awareness
MBCT: Mindfulness-Based Cognitive Therapy
MBSR: Mindfulness-Based Stress Reduction
MSC: Mindful Self-Compassion
PHQ: Patient Health Questionnaire
PSS: Perceived Stress Scale
PSWQ: Penn State Worry Questionnaire
SOCS: Sussex-Oxford Compassion Scale
S-SCS: State Self Compassion Scale
SST: Scrambled Sentences Task
STAI: State-Trait Anxiety Inventory
TAS: Toronto Alexithymia Scale
ToM: Theory of Mind
TSST: Trier Social Stress Test
ZPG: Zurich Prosocial Game

## References

[1] Hossain MM, Tasnim S, Sultana A, Faizah F, Mazumder H, Zou L, et al. Epidemiology of mental health problems in COVID-19: A review [Internet]. Vol. 9, F1000Research. F1000 Research Ltd; 2020 [cited 2021 Jun 1]. Available from: /pmc/articles/PMC7549174/ doi: 10.12688/f1000research.24457.1 33093946 PMC7549174

[2] Tsamakis K, Tsiptsios D, Ouranidis A, Mueller C, Schizas D, Terniotis C, et al. COVID-19 and its consequences on mental health (Review). Experimental and Therapeutic Medicine [Internet]. 2021 Jan 22 [cited 2021 Jun 1];21(3):1–1. Available from: http://www.spandidos-publications.com/10.3892/etm.2021.9675/abstract 33603852 10.3892/etm.2021.9675 PMC7851613

[3] Vindegaard N, Benros ME. COVID-19 pandemic and mental health consequences: Systematic review of the current evidence. Vol. 89, Brain, Behavior, and Immunity. Academic Press Inc.; 2020. p. 531–42. doi: 10.1016/j.bbi.2020.05.048 32485289 PMC7260522

[4] Li S, Wang Y, Xue J, Zhao N, Zhu T. The impact of covid-19 epidemic declaration on psychological consequences: A study on active weibo users. International Journal of Environmental Research and Public Health [Internet]. 2020 Mar 2 [cited 2021 Jun 1];17(6):2032. Available from: www.mdpi.com/journal/ijerph 10.3390/ijerph17062032 PMC7143846 32204411

[5] Moccia L, Janiri D, Pepe M, Dattoli L, Molinaro M, de Martin V, et al. Affective temperament, attachment style, and the psychological impact of the COVID-19 outbreak: an early report on the Italian general population. Brain, Behavior, and Immunity. 2020 Jul 1;87:75–9. 10.1016/j.bbi.2020.04.048 PMC7169930 32325098

[6] Özdin S, Bayrak Özdin Ş. Levels and predictors of anxiety, depression and health anxiety during COVID-19 pandemic in Turkish society: The importance of gender. International Journal of Social Psychiatry [Internet]. 2020 Aug 1 [cited 2021 Jun 1];66(5):504–11. Available from: 10.1177/0020764020927051 32380879 PMC7405629

[7] Roy D, Tripathy S, Kar SK, Sharma N, Verma SK, Kaushal V. Study of knowledge, attitude, anxiety & perceived mental healthcare need in Indian population during COVID-19 pandemic. Asian Journal of Psychiatry. 2020 Jun 1;51:102083. doi: 10.1016/j.ajp.2020.102083 32283510 PMC7139237

[8] Sønderskov KM, Dinesen PT, Santini ZI, Østergaard SD. The depressive state of Denmark during the COVID-19 pandemic. Acta Neuropsychiatrica. 2020 Aug 22;32(4). doi: 10.1017/neu.2020.15 32319879 PMC7176490

[9] Gautam M, Thakrar A, Akinyemi E, Mahr G. Current and Future Challenges in the Delivery of Mental Healthcare during COVID-19. SN Comprehensive Clinical Medicine [Internet]. 2020 Jul 11 [cited 2021 Jun 1];2(7):865–70. Available from: 10.1007/s42399-020-00348-3 32838140 PMC7287405

[10] Moreno C, Wykes T, Galderisi S, Nordentoft M, Crossley N, Jones N, et al. How mental health care should change as a consequence of the COVID-19 pandemic. Vol. 7, The Lancet Psychiatry. Elsevier Ltd; 2020. p. 813–24. doi: 10.1016/S2215-0366(20)30307-2 32682460 PMC7365642

[11] Cai W, Lian B, Song X, Hou T, Deng G, Li H. A cross-sectional study on mental health among health care workers during the outbreak of Corona Virus Disease 2019. Asian Journal of Psychiatry. 2020 Jun 1;51:102111. doi: 10.1016/j.ajp.2020.102111 32361388 PMC7194661

[12] Huang Y, Zhao N. Chinese mental health burden during the COVID-19 pandemic. Vol. 51, Asian Journal of Psychiatry. Elsevier B.V.; 2020. p. 102052. 10.1016/j.ajp.2020.102052 PMC7195325 32361387

[13] Xu J, Xu Q hui, Wang C ming, Wang J. Psychological status of surgical staff during the COVID-19 outbreak. Vol. 288, Psychiatry Research. Elsevier Ireland Ltd; 2020. p. 112955. doi: 10.1016/j.psychres.2020.112955 32302815 PMC7151272

[14] Loades ME, Chatburn E, Higson-Sweeney N, Reynolds S, Shafran R, Brigden A, et al. Rapid Systematic Review: The Impact of Social Isolation and Loneliness on the Mental Health of Children and Adolescents in the Context of COVID-19. Vol. 59, Journal of the American Academy of Child and Adolescent Psychiatry. Elsevier Inc.; 2020. p. 1218–1239.e3. doi: 10.1016/j.jaac.2020.05.009 32504808 PMC7267797

[15] Gloster AT, Lamnisos D, Lubenko J, Presti G, Squatrito V, Constantinou M, et al. Impact of COVID-19 pandemic on mental health: An international study. PLoS ONE [Internet]. 2020 Dec 1 [cited 2021 Jun 1];15(12 December):e0244809. Available from: 10.1371/journal.pone.0244809 33382859 PMC7774914

[16] Liu N, Zhang F, Wei C, Jia Y, Shang Z, Sun L, et al. Prevalence and predictors of PTSS during COVID-19 outbreak in China hardest-hit areas: Gender differences matter. Psychiatry Research. 2020 May 1;287:112921. doi: 10.1016/j.psychres.2020.112921 32240896 PMC7102622

[17] Wang C, Pan R, Wan X, Tan Y, Xu L, Ho CS, et al. Immediate psychological responses and associated factors during the initial stage of the 2019 coronavirus disease (COVID-19) epidemic among the general population in China. International Journal of Environmental Research and Public Health [Internet]. 2020 Mar 1 [cited 2021 Jun 1];17(5):1729. Available from: www.mdpi.com/journal/ijerph 32155789 10.3390/ijerph17051729 PMC7084952

[18] Huang Y, Zhao N. Mental health burden for the public affected by the COVID-19 outbreak in China: Who will be the high-risk group? [Internet]. Vol. 26, Psychology, Health and Medicine. Routledge; 2021 [cited 2021 Jun 1]. p. 23–34. Available from: 10.1080/13548506.2020.1754438 32286091

[19] Prati G. Mental health and its psychosocial predictors during national quarantine in Italy against the coronavirus disease 2019 (COVID-19). Anxiety, Stress and Coping [Internet]. 2021 [cited 2021 Jun 1];34(2):145–56. Available from: 10.1080/10615806.2020.1861253 33350343

[20] Bendau A, Plag J, Kunas S, Wyka S, Ströhle A, Petzold MB. Longitudinal changes in anxiety and psychological distress, and associated risk and protective factors during the first three months of the COVID‐19 pandemic in Germany. Brain and Behavior. 2021 Feb 23;11(2). 10.1002/brb3.1964 PMC7744907 33230969

[21] Pakenham KI, Landi G, Boccolini G, Furlani A, Grandi S, Tossani E. The moderating roles of psychological flexibility and inflexibility on the mental health impacts of COVID-19 pandemic and lockdown in Italy. Journal of Contextual Behavioral Science. 2020 Jul 1;17:109–18. doi: 10.1016/j.jcbs.2020.07.003 32834969 PMC7370913

[22] Smith BM, Twohy AJ, Smith GS. Psychological inflexibility and intolerance of uncertainty moderate the relationship between social isolation and mental health outcomes during COVID-19. Journal of Contextual Behavioral Science. 2020 Oct 1;18:162–74. doi: 10.1016/j.jcbs.2020.09.005 32953435 PMC7489247

[23] Dimanova P, Borbás R, Schnider CB, Fehlbaum L, Raschle N. Direct and indirect effects of dorsolateral prefrontal cortex and emotion regulation strategy use on mental health during Covid-19. [cited 2021 Jun 1]; https://psyarxiv.com/bmgt9/

[24] Luu TT. Worker resilience during the COVID-19 crisis: The role of core beliefs challenge, emotion regulation, and family strain. Personality and Individual Differences. 2021 Sep 1;179:110784. 10.1016/j.paid.2021.110784 PMC9756412 36540083

[25] Panayiotou G, Panteli M, Leonidou C. Coping with the invisible enemy: The role of emotion regulation and awareness in quality of life during the COVID-19 pandemic. Journal of Contextual Behavioral Science. 2021 Jan 1;19:17–27.

[26] Groarke JM, Berry E, Graham-Wisener L, McKenna-Plumley PE, McGlinchey E, Armour C. Loneliness in the UK during the COVID-19 pandemic: Cross-sectional results from the COVID-19 Psychological Wellbeing Study. PLoS ONE [Internet]. 2020 Sep 1 [cited 2021 Jun 1];15(9 September):e0239698. Available from: 10.1371/journal.pone.0239698 32970764 PMC7513993

[27] Sibel Demirtas A. Predictive roles of state hope and cognitive control/flexibility in state anxiety during COVID-19 outbreak in Turkey. Turkey Dusunen Adam The Journal of Psychiatry and Neurological Sciences. 2021;34:89–96.

[28] Luthar SS, Cicchetti D, Becker B. Research on resilience: Response to commentaries. Child Development [Internet]. 2000 May 1 [cited 2021 Jun 1];71(3):573–5. Available from: https://srcd.onlinelibrary.wiley.com/doi/full/10.1111/1467-8624.00168

[29] Blanc J, Briggs AQ, Seixas AA, Reid M, Jean-Louis G, Pandi-Perumal SR. Addressing psychological resilience during the coronavirus disease 2019 pandemic: a rapid review [Internet]. Vol. 34, Current opinion in psychiatry. NLM (Medline); 2021 [cited 2021 Jun 1]. p. 29–35. Available from: https://journals.lww.com/co-psychiatry/Fulltext/2021/01000/Addressing_psychological_resilience_during_the.5.aspx 33230041 10.1097/YCO.0000000000000665 PMC7751836

[30] Killgore WDS, Taylor EC, Cloonan SA, Dailey NS. Psychological resilience during the COVID-19 lockdown. Vol. 291, Psychiatry Research. Elsevier Ireland Ltd; 2020. p. 113216. doi: 10.1016/j.psychres.2020.113216 32544705 PMC7280133

[31] Veer IM, Riepenhausen A, Zerban M, Wackerhagen C, Puhlmann LMC, Engen H, et al. Psycho-social factors associated with mental resilience in the Corona lockdown. Translational Psychiatry [Internet]. 2021 Jun 1 [cited 2021 Jun 1];11(1):15. Available from: 10.1038/s41398-020-01150-4 33479211 PMC7817958

[32] Chong YY, Chien WT, Cheng HY, Lamnisos D, Ļubenko J, Presti G, et al. Patterns of psychological responses among the public during the early phase of covid-19: A cross-regional analysis. International Journal of Environmental Research and Public Health [Internet]. 2021 Apr 2 [cited 2021 Jun 1];18(8):4143. Available from: 10.3390/ijerph18084143 33919888 PMC8070933

[33] Borkowska M, Laurence J. Coming together or coming apart? Changes in social cohesion during the Covid-19 pandemic in England. European Societies [Internet]. 2021 [cited 2021 Jun 1];23(S1):S618–36. Available from: 10.1080/14616696.2020.1833067

[34] Best LA, Law MA, Roach S, Wilbiks JMP. The Psychological Impact of COVID-19 in Canada: Effects of Social Isolation During the Initial Response. Canadian Psychology [Internet]. 2020 [cited 2021 Jun 1]; Available from: https://psycnet.apa.org/journals/cap/62/1/143 34219905

[35] Matos M, McEwan K, Kanovský M, Halamová J, Steindl SR, Ferreira N, et al. Fears of compassion magnify the harmful effects of threat of COVID‐19 on mental health and social safeness across 21 countries. Clinical Psychology & Psychotherapy. 2021 May 15. doi: 10.1002/cpp.2601 33880832 PMC8251194

[36] van de Groep S, Zanolie K, Green KH, Sweijen SW, Crone EA. A daily diary study on adolescents’ mood, empathy, and prosocial behavior during the COVID-19 pandemic. PLoS ONE [Internet]. 2020 Oct 1 [cited 2021 Jun 1];15(10 October):e0240349. Available from: 10.1371/journal.pone.0240349 33027308 PMC7540854

[37] Corvo E, de Caro W. COVID-19 and spontaneous singing to decrease loneliness, improve cohesion, and mental well-being: An Italian experience. Psychological Trauma: Theory, Research, Practice, and Policy. 2020 Aug;12(S1). 10.1037/tra0000838 32584108

[38] Kabat-Zinn J. Mindfulness-Based Interventions in Context: Past, Present, and Future. Clinical Psychology: Science & Practice. 2003;10(2):144–56.

[39] Teasdale JD, Segal Z v., Williams JMG, Ridgewaya VA, Soulsby JM, Lau MA. Prevention of relapse/recurrence in major depression by mindfulness-based cognitive therapy. Journal of Consulting and Clinical Psychology [Internet]. 2000 [cited 2021 Jun 1];68(4):615–23. Available from: /fulltext/2000-05084-010.html doi: 10.1037//0022-006x.68.4.615 10965637

[40] Germer CK, Neff KD. Self-compassion in clinical practice. Journal of Clinical Psychology [Internet]. 2013 Aug 1 [cited 2021 Jun 1];69(8):856–67. Available from: https://onlinelibrary.wiley.com/doi/full/10.1002/jclp.22021 23775511 10.1002/jclp.22021

[41] Gilbert P, Procter S. Compassionate mind training for people with high shame and self-criticism: Overview and pilot study of a group therapy approach [Internet]. Vol. 13, Clinical Psychology and Psychotherapy. John Wiley & Sons, Ltd; 2006 [cited 2021 Jun 1]. p. 353–79. Available from: www.interscience.wiley.com

[42] Gu J, Strauss C, Bond R, Cavanagh K. How do mindfulness-based cognitive therapy and mindfulness-based stress reduction improve mental health and wellbeing? A systematic review and meta-analysis of mediation studies. Vol. 37, Clinical Psychology Review. Elsevier Inc.; 2015. p. 1–12. doi: 10.1016/j.cpr.2015.01.006 25689576

[43] Leaviss J, Uttley L. Psychotherapeutic benefits of compassion-focused therapy: An early systematic review [Internet]. Vol. 45, Psychological Medicine. Cambridge University Press; 2015 [cited 2021 Jun 1]. p. 927–45. Available from: 10.1017/S0033291714002141 PMC4413786 25215860

[44] Spijkerman MPJ, Pots WTM, Bohlmeijer ET. Effectiveness of online mindfulness-based interventions in improving mental health: A review and meta-analysis of randomised controlled trials. Vol. 45, Clinical Psychology Review. Elsevier Inc.; 2016. p. 102–14. doi: 10.1016/j.cpr.2016.03.009 27111302

[45] Wilson AC, Mackintosh K, Power K, Chan SWY. Effectiveness of Self-Compassion Related Therapies: a Systematic Review and Meta-analysis [Internet]. Vol. 10, Mindfulness. Springer New York LLC; 2019 [cited 2021 Jun 1]. p. 979–95. Available from: 10.1007/s12671-018-1037-6

[46] Brito G, Seculares E, Compasión L. Secular Compassion Training: An Empirical Review. Journal of Transpersonal Research. 2014;6(2):61–71.

[47] Chiesa A, Serretti A. Mindfulness-based stress reduction for stress management in healthy people: A review and meta-analysis. Journal of Alternative and Complementary Medicine [Internet]. 2009 May 1 [cited 2021 Jun 1];15(5):593–600. Available from: www.liebertpub.com 19432513 10.1089/acm.2008.0495

[48] Neff KD, Germer CK. A Pilot Study and Randomized Controlled Trial of the Mindful Self-Compassion Program. Journal of Clinical Psychology. 2013 Jan;69(1). doi: 10.1002/jclp.21923 23070875

[49] Black DS, Slavich GM. Mindfulness meditation and the immune system: a systematic review of randomized controlled trials. Annals of the New York Academy of Sciences [Internet]. 2016 Jun 1 [cited 2021 Jun 1];1373(1):13–24. Available from: /pmc/articles/PMC4940234/ doi: 10.1111/nyas.12998 26799456 PMC4940234

[50] Pace TWW, Negi LT, Adame DD, Cole SP, Sivilli TI, Brown TD, et al. Effect of compassion meditation on neuroendocrine, innate immune and behavioral responses to psychosocial stress. Psychoneuroendocrinology. 2009 Jan 1;34(1):87–98. doi: 10.1016/j.psyneuen.2008.08.011 18835662 PMC2695992

[51] Finlay-Jones A, Kane R, Rees C. Self-Compassion Online: A Pilot Study of an Internet-Based Self-Compassion Cultivation Program for Psychology Trainees. Journal of Clinical Psychology. 2017 Jul;73(7). doi: 10.1002/jclp.22375 27787877

[52] Guendelman S, Medeiros S, Rampes H. Mindfulness and emotion regulation: Insights from neurobiological, psychological, and clinical studies [Internet]. Vol. 8, Frontiers in Psychology. Frontiers Research Foundation; 2017 [cited 2021 Jun 1]. p. 220. Available from: www.frontiersin.org 10.3389/fpsyg.2017.00220 PMC5337506 28321194

[53] Donald JN, Sahdra BK, van Zanden B, Duineveld JJ, Atkins PWB, Marshall SL, et al. Does your mindfulness benefit others? A systematic review and meta-analysis of the link between mindfulness and prosocial behaviour. British Journal of Psychology [Internet]. 2019 Feb 1 [cited 2021 Jun 1];110(1):101–25. Available from: www.wileyonlinelibrary.com 30094812 10.1111/bjop.12338

[54] Luberto CM, Shinday N, Song R, Philpotts LL, Park ER, Fricchione GL, et al. A Systematic Review and Meta-analysis of the Effects of Meditation on Empathy, Compassion, and Prosocial Behaviors [Internet]. Vol. 9, Mindfulness. Springer New York LLC; 2018 [cited 2021 Jun 1]. p. 708–24. Available from: https://link.springer.com/article/10.1007/s12671-017-0841-8 10.1007/s12671-017-0841-8 PMC6081743 30100929

[55] Neff KD, Dahm KA. Self-Compassion: What It Is, What It Does, and How It Relates to Mindfulness. In: Handbook of Mindfulness and Self-Regulation. New York, NY: Springer New York; 2015.

[56] Baer RA. Mindfulness training as a clinical intervention: A conceptual and empirical review. Clinical Psychology: Science and Practice [Internet]. 2003 Summer 1 [cited 2021 Jun 1];10(2):125–43. Available from: https://onlinelibrary.wiley.com/doi/full/10.1093/clipsy.bpg015

[57] Creswell JD. Mindfulness Interventions. Annual Review of Psychology. 2017 Jan 3;68(1). doi: 10.1146/annurev-psych-042716-051139 27687118

[58] Dimidjian S, Segal Z v. Prospects for a clinical science of mindfulness-based intervention. American Psychologist [Internet]. 2015 Oct 1 [cited 2021 Jun 1];70(7):593–620. Available from: 10.1037/cap0000254 26436311 PMC5853107

[59] Chiesa A, Serretti A. Mindfulness-based interventions for chronic pain: A systematic review of the evidence [Internet]. Vol. 17, Journal of Alternative and Complementary Medicine. Mary Ann Liebert, Inc. 140 Huguenot Street, 3rd Floor New Rochelle, NY 10801 USA; 2011 [cited 2021 Jun 1]. p. 83–93. Available from: www.liebertpub.com 10.1089/acm.2009.0546 21265650

[60] Bowen S, Witkiewitz K, Clifasefi SL, Grow J, Chawla N, Hsu SH, et al. Relative efficacy of mindfulness-based relapse prevention, standard relapse prevention, and treatment as usual for substance use disorders. JAMA Psychiatry [Internet]. 2014 May 1 [cited 2021 Jun 1];71(5):547–56. Available from: https://jamanetwork.com/ 24647726 10.1001/jamapsychiatry.2013.4546 PMC4489711

[61] Mason AE, Epel ES, Kristeller J, Moran PJ, Dallman M, Lustig RH, et al. Effects of a mindfulness-based intervention on mindful eating, sweets consumption, and fasting glucose levels in obese adults: data from the SHINE randomized controlled trial. Journal of Behavioral Medicine [Internet]. 2016 Apr 1 [cited 2021 Jun 1];39(2):201–13. Available from: https://link.springer.com/article/10.1007/s10865-015-9692-8 26563148 10.1007/s10865-015-9692-8 PMC4801689

[62] Joyce S, Shand F, Tighe J, Laurent SJ, Bryant RA, Harvey SB. Road to resilience: A systematic review and meta-analysis of resilience training programmes and interventions [Internet]. Vol. 8, BMJ Open. BMJ Publishing Group; 2018 [cited 2021 Jun 1]. p. 17858. Available from: http://bmjopen.bmj.com/ 29903782 10.1136/bmjopen-2017-017858 PMC6009510

[63] Carson JW, Carson JW, Carson KM, Gil KM, Baucom DH. Mindfulness-based relationship enhancement. Behavior Therapy. 2004 [cited 2021 Jun 1]; http://citeseerx.ist.psu.edu/viewdoc/summary?doi=10.1.1.465.5158

[64] Medeiros S, Guendelman S. A Socio-affective, Developmentally Informed Perspective for Contemplative Practices in Adolescence: Towards Resilient Communities. In: Enhancing Resilience in Youth. Cham: Springer International Publishing; 2019.

[65] Jazaieri H, McGonigal K, Jinpa T, Doty JR, Gross JJ, Goldin PR. A randomized controlled trial of compassion cultivation training: Effects on mindfulness, affect, and emotion regulation. Motivation and Emotion. 2014 Feb 13;38(1).

[66] Kirby JN, Tellegen CL, Steindl SR. A Meta-Analysis of Compassion-Based Interventions: Current State of Knowledge and Future Directions. Behavior Therapy. 2017 Nov;48(6). doi: 10.1016/j.beth.2017.06.003 29029675

[67] Singer T, Kok BE, Bornemann B, Zuborg S, Bolz M, Bochow CA. The ReSource project: Background, design, samples, and measurements. 2nd ed. Leipzig, Germany: Max Planck Institute for Human Cognitive and Brain Sciences; 2016.

[68] Singer T, Engert V. It matters what you practice: differential training effects on subjective experience, behavior, brain and body in the ReSource Project. Vol. 28, Current Opinion in Psychology. Elsevier B.V.; 2019. p. 151–8. doi: 10.1016/j.copsyc.2018.12.005 30684917

[69] Kok BE, Singer T. Effects of contemplative dyads on engagement and perceived social connectedness over 9 months of mental training a randomized clinical trial. JAMA Psychiatry [Internet]. 2017 Feb 1 [cited 2021 Jun 1];74(2):126–34. Available from: https://jamanetwork.com/ 28030741 10.1001/jamapsychiatry.2016.3360

[70] Kok BE, Singer T. Phenomenological Fingerprints of Four Meditations: Differential State Changes in Affect, Mind-Wandering, Meta-Cognition, and Interoception Before and After Daily Practice Across 9 Months of Training. Mindfulness [Internet]. 2017 Feb 1 [cited 2021 Jun 1];8(1):218–31. Available from: https://link.springer.com/article/10.1007/s12671-016-0594-9 28163798 10.1007/s12671-016-0594-9 PMC5241345

[71] Trautwein FM, Kanske P, Böckler A, Singer T. Differential benefits of mental training types for attention, compassion, and theory of mind. Cognition. 2020 Jan 1;194:104039. doi: 10.1016/j.cognition.2019.104039 31450018 PMC6891878

[72] Hildebrandt LK, McCall C, Singer T. Socioaffective versus sociocognitive mental trainings differentially affect emotion regulation strategies. Emotion [Internet]. 2019 Dec 1 [cited 2021 Jun 1];19(8):1329–42. Available from: https://psycnet.apa.org/journals/emo/19/8/1329 30589299 10.1037/emo0000518

[73] Hildebrandt LK, McCall C, Singer T. Differential Effects of Attention-, Compassion-, and Socio-Cognitively Based Mental Practices on Self-Reports of Mindfulness and Compassion. Mindfulness. 2017 Dec 6;8(6). 10.1007/s12671-017-0716-z PMC5693975 29201246

[74] Böckler A, Tusche A, Schmidt P, Singer T. Distinct mental trainings differentially affect altruistically motivated, norm motivated, and self-reported prosocial behaviour. Scientific Reports [Internet]. 2018 Dec 1 [cited 2021 Jun 1];8(1):13560. Available from: www.nature.com/scientificreports/ 30202029 10.1038/s41598-018-31813-8 PMC6131389

[75] Valk SL, Bernhardt BC, Trautwein FM, Böckler A, Kanske P, Guizard N, et al. Structural plasticity of the social brain: Differential change after socio-affective and cognitive mental training. Science Advances [Internet]. 2017 Oct 1 [cited 2021 Jun 1];3(10):e1700489. Available from: http://advances.sciencemag.org/ 28983507 10.1126/sciadv.1700489 PMC5627980

[76] Engert V, Kok BE, Papassotiriou I, Chrousos GP, Singer T. Specific reduction in cortisol stress reactivity after social but not attention-based mental training. Science Advances [Internet]. 2017 Oct 1 [cited 2021 Jun 1];3(10):e1700495. Available from: http://advances.sciencemag.org/ 28983508 10.1126/sciadv.1700495 PMC5627978

[77] Rauschenberg C, Schick A, Hirjak D, Seidler A, Paetzold I, Apfelbacher C, et al. Evidence synthesis of digital interventions to mitigate the negative impact of the COVID-19 pandemic on public mental health: Rapid meta-review [Internet]. Vol. 23, Journal of Medical Internet Research. JMIR Publications Inc.; 2021 [cited 2021 Jun 1]. p. e23365. Available from: https://www.jmir.org/2021/3/e23365 33606657 10.2196/23365 PMC7951054

[78] Cahn BR, Polich J. Meditation states and traits: EEG, ERP, and neuroimaging studies. Psychological Bulletin [Internet]. 2006 Mar [cited 2021 Jun 1];132(2):180–211. Available from: 10.1037/cap0000254 16536641

[79] Raffone A, Srinivasan N. The exploration of meditation in the neuroscience of attention and consciousness. 10.1007/s10339-009-0354-z 20041276

[80] Grossman P, Niemann L, Schmidt S, Walach H. Mindfulness-based stress reduction and health benefits: A meta-analysis. Journal of Psychosomatic Research. 2004 Jul 1;57(1):35–43. doi: 10.1016/S0022-3999(03)00573-7 15256293

[81] Khoury B, Sharma M, Rush SE, Fournier C. Mindfulness-based stress reduction for healthy individuals: A meta-analysis. Vol. 78, Journal of Psychosomatic Research. Elsevier Inc.; 2015. p. 519–28. doi: 10.1016/j.jpsychores.2015.03.009 25818837

[82] Goldin PR, Gross JJ. Effects of Mindfulness-Based Stress Reduction (MBSR) on Emotion Regulation in Social Anxiety Disorder. Emotion [Internet]. 2010 Feb [cited 2021 Jun 1];10(1):83–91. Available from: https://psycnet.apa.org/journals/emo/10/1/83 20141305 10.1037/a0018441 PMC4203918

[83] Bornemann B, Herbert BM, Mehling WE, Singer T. Differential changes in self-reported aspects of interoceptive awareness through 3 months of contemplative training. Frontiers in Psychology [Internet]. 2014 Jan 6 [cited 2021 Jun 1];5(OCT):1504. Available from: www.frontiersin.org 25610410 10.3389/fpsyg.2014.01504 PMC4284997

[84] Bornemann B, Singer T. Taking time to feel our body: Steady increases in heartbeat perception accuracy and decreases in alexithymia over 9 months of contemplative mental training. Psychophysiology [Internet]. 2017 Mar 1 [cited 2021 Jun 1];54(3):469–82. Available from: https://onlinelibrary.wiley.com/doi/full/10.1111/psyp.12790 27925645 10.1111/psyp.12790

[85] Bornemann B, Kovacs P, Singer T. Voluntary upregulation of heart rate variability through biofeedback is improved by mental contemplative training. Scientific Reports [Internet]. 2019 Dec 1 [cited 2021 Jun 1];9(1):1–13. Available from: 10.1038/s41598-019-44201-7 31133673 PMC6536553

[86] Hanley AW, Mehling WE, Garland EL. Holding the body in mind: Interoceptive awareness, dispositional mindfulness and psychological well-being. Journal of Psychosomatic Research. 2017 Aug;99. 10.1016/j.jpsychores.2017.05.014 PMC5522814 28712417

[87] Kikusui T, Winslow JT, Mori Y. Social buffering: Relief from stress and anxiety [Internet]. Vol. 361, Philosophical Transactions of the Royal Society B: Biological Sciences. Royal Society; 2006 [cited 2021 Jun 1]. p. 2215–28. Available from: https://royalsocietypublishing.org/ 17118934 10.1098/rstb.2006.1941 PMC1764848

[88] Bergen-Cico D, Possemato K, Cheon S. Examining the efficacy of a brief mindfulness-based stress reduction (brief MBSR) program on psychological health. Journal of American College Health [Internet]. 2013 Aug 1 [cited 2021 Jun 1];61(6):348–60. Available from: https://www.tandfonline.com/doi/abs/10.1080/07448481.2013.813853 doi: 10.1080/07448481.2013.813853 23930749

[89] Birnie K, Speca M, Carlson LE. Exploring self-compassion and empathy in the context of mindfulness-based stress reduction (MBSR). Stress and Health [Internet]. 2010 Dec 1 [cited 2021 Jun 1];26(5):359–71. Available from: https://onlinelibrary.wiley.com/doi/full/10.1002/smi.1305

[90] Ramaci T, Bellini D, Presti G, Santisi G. Psychological Flexibility and Mindfulness as Predictors of Individual Outcomes in Hospital Health Workers. Frontiers in Psychology. 2019 Jun 12;10. doi: 10.3389/fpsyg.2019.01302 31249541 PMC6582771

[91] de Lissnyder E, Koster EHW, Goubert L, Onraedt T, Vanderhasselt MA, de Raedt R. Cognitive control moderates the association between stress and rumination. Journal of Behavior Therapy and Experimental Psychiatry. 2012 Mar 1;43(1):519–25. doi: 10.1016/j.jbtep.2011.07.004 21813083

[92] Carmody J, Baer RA, Lykins ELB, Olendzki N. An empirical study of the mechanisms of mindfulness in a mindfulness-based stress reduction program. Journal of Clinical Psychology [Internet]. 2009 Jun 1 [cited 2021 Jun 1];65(6):613–26. Available from: www.interscience.wiley.com 19267330 10.1002/jclp.20579

[93] Cobb S. Social support as a moderator of life stress. Psychosomatic Medicine [Internet]. 1976 [cited 2021 Jun 1];38(5):300–14. Available from: /record/1977-13045-001 doi: 10.1097/00006842-197609000-00003 981490

[94] Morrison R O’Connor RC. Predicting psychological distress in college students: The role of rumination and stress. Journal of Clinical Psychology [Internet]. 2005 Apr 1 [cited 2021 Jun 1];61(4):447–60. Available from: www.interscience.wiley.com 15468342 10.1002/jclp.20021

[95] Brosschot JF, Gerin W, Thayer JF. The perseverative cognition hypothesis: A review of worry, prolonged stress-related physiological activation, and health. Vol. 60, Journal of Psychosomatic Research. Elsevier; 2006. p. 113–24. 10.1016/j.jpsychores.2005.06.074 16439263

[96] Gilbert P, McEwan K, Matos M, Rivis A. Fears of compassion: Development of three self-report measures. Psychology and Psychotherapy: Theory, Research and Practice [Internet]. 2011 Sep 1 [cited 2021 Jun 1];84(3):239–55. Available from: www.wileyonlinelibrary.com 10.1348/147608310X526511 22903867

[97] Doane LD, Adam EK. Loneliness and cortisol: Momentary, day-to-day, and trait associations. Psychoneuroendocrinology. 2010 Apr 1;35(3):430–41. doi: 10.1016/j.psyneuen.2009.08.005 19744794 PMC2841363

[98] Hawkley LC, Cacioppo JT. Loneliness and pathways to disease. In: Brain, Behavior, and Immunity. Academic Press Inc.; 2003. p. 98–105. 10.1016/s0889-1591(02)00073-9 12615193

[99] Linz R, Singer T, Engert V. Interactions of momentary thought content and subjective stress predict cortisol fluctuations in a daily life experience sampling study. Scientific Reports [Internet]. 2018 Dec 1 [cited 2021 Jun 1];8(1):1–11. Available from: www.nature.com/scientificreports 30337580 10.1038/s41598-018-33708-0 PMC6193976

[100] Ruby FJM, Smallwood J, Engen H, Singer T. How Self-Generated Thought Shapes Mood-The Relation between Mind-Wandering and Mood Depends on the Socio-Temporal Content of Thoughts. PLoS ONE [Internet]. 2013 Oct 23 [cited 2021 Jun 1];8(10):e77554. Available from: www.plosone.org 24194889 10.1371/journal.pone.0077554 PMC3806791

[101] Snippe E, Viechtbauer W, Geschwind N, Klippel A, de Jonge P, Wichers M. The Impact of Treatments for Depression on the Dynamic Network Structure of Mental States: Two Randomized Controlled Trials. Scientific Reports. 2017 Jun 20;7(1). doi: 10.1038/srep46523 28425449 PMC5397847

[102] Creswell JD, Irwin MR, Burklund LJ, Lieberman MD, Arevalo JMG, Ma J, et al. Mindfulness-Based Stress Reduction training reduces loneliness and pro-inflammatory gene expression in older adults: A small randomized controlled trial. Brain, Behavior, and Immunity. 2012 Oct;26(7). doi: 10.1016/j.bbi.2012.07.006 22820409 PMC3635809

[103] Jin Y, Zhang M, Wang Y, An J. The relationship between trait mindfulness, loneliness, regulatory emotional self-efficacy, and subjective well-being. Personality and Individual Differences. 2020 Feb;154. doi: 10.1016/j.paid.2019.109711 32308249 PMC7164798

[104] Veronese N, Galvano D, D’Antiga F, Vecchiato C, Furegon E, Allocco R, et al. Interventions for reducing loneliness: An umbrella review of intervention studies. Health & Social Care in the Community. 2020 Dec 5. 10.1111/hsc.13248 33278311

[105] Lee RM, Robbins SB. The relationship between social connectedness and anxiety, self-esteem, and social identity. Journal of Counseling Psychology. 1998 Jul;45(3).

[106] Steptoe A, Owen N, Kunz-Ebrecht SR, Brydon L. Loneliness and neuroendocrine, cardiovascular, and inflammatory stress responses in middle-aged men and women. Psychoneuroendocrinology. 2004 Jun 1;29(5):593–611. doi: 10.1016/S0306-4530(03)00086-6 15041083

[107] Queen TL, Stawski RS, Ryan LH, Smith J. Loneliness in a day: Activity engagement, time alone, and experienced emotions. Psychology and Aging [Internet]. 2014 [cited 2021 Jun 1];29(2):297–305. Available from: https://psycnet.apa.org/journals/pag/29/2/297 24955998 10.1037/a0036889 PMC4161136

[108] Twenge JM, Spitzberg BH, Campbell WK. Less in-person social interaction with peers among U.S. adolescents in the 21st century and links to loneliness. Journal of Social and Personal Relationships [Internet]. 2019 Jun 1 [cited 2021 Jun 1];36(6):1892–913. Available from: https://journals.sagepub.com/doi/full/10.1177/0265407519836170

[109] Prieto-Flores ME, Fernandez-Mayoralas G, Forjaz MJ, Rojo-Perez F, Martinez-Martin P. Residential satisfaction, sense of belonging and loneliness among older adults living in the community and in care facilities. Health and Place. 2011 Nov 1;17(6):1183–90. doi: 10.1016/j.healthplace.2011.08.012 21924944

[110] Akin A. Self-compassion and Loneliness. International Online Journal of Educational Sciences [Internet]. 2010 [cited 2021 Jun 1];2(3):702–18. Available from: www.iojes.net

[111] Liu X, Yang Y, Wu H, Kong X, Cui L. The roles of fear of negative evaluation and social anxiety in the relationship between self-compassion and loneliness: a serial mediation model. Current Psychology [Internet]. 2020 Sep 4 [cited 2021 Jun 1];1–9. Available from: https://link.springer.com/article/10.1007/s12144-020-01001-x

[112] Segrin C, Domschke T. Social support, loneliness, recuperative processes, and their direct and indirect effects on health. Health Communication [Internet]. 2011 Apr [cited 2021 Jun 1];26(3):221–32. Available from: https://www.tandfonline.com/doi/abs/10.1080/10410236.2010.546771 21318918 10.1080/10410236.2010.546771

[113] Beck A T, Steer R A, Brown G. Beck depression inventory–II. Psychological Assessment. 1996.

[114] Spielberger CD. State-Trait Anxiety Inventory. In: The Corsini Encyclopedia of Psychology. Hoboken, NJ, USA: John Wiley & Sons, Inc.; 2010.

[115] Fjorback LO, Arendt M, Ornbol E, Fink P, Walach H. Mindfulness-based stress reduction and mindfulness-based cognitive therapy—a systematic review of randomized controlled trials [Internet]. Vol. 124, Acta Psychiatrica Scandinavica. John Wiley & Sons, Ltd; 2011 [cited 2021 Jun 1]. p. 102–19. Available from: https://onlinelibrary.wiley.com/doi/full/10.1111/j.1600-0447.2011.01704.x 21534932 10.1111/j.1600-0447.2011.01704.x

[116] Goldberg SB, Tucker RP, Greene PA, Davidson RJ, Wampold BE, Kearney DJ, et al. Mindfulness-based interventions for psychiatric disorders: A systematic review and meta-analysis. Vol. 59, Clinical Psychology Review. Elsevier Inc.; 2018. p. 52–60. doi: 10.1016/j.cpr.2017.10.011 29126747 PMC5741505

[117] Frostadottir AD, Dorjee D. Effects of mindfulness based cognitive therapy (MBCT) and compassion focused therapy (CFT) on symptom change, mindfulness, self-compassion, and rumination in clients with depression, anxiety, and stress. Frontiers in Psychology [Internet]. 2019 May 17 [cited 2021 Jun 1];10(MAY):1099. Available from: www.frontiersin.org 31164849 10.3389/fpsyg.2019.01099 PMC6534108

[118] Farb N A S, Anderson A K, Irving J A, Segal Z V. Mindfulness interventions and emotion regulation. In: Gross, editor. Handbook of emotion regulation. The Guilford Press; 2014. p. 548–67.

[119] Wheeler MS, Arnkoff DB, Glass CR. The Neuroscience of Mindfulness: How Mindfulness Alters the Brain and Facilitates Emotion Regulation [Internet]. Vol. 8, Mindfulness. Springer New York LLC; 2017 [cited 2021 Jun 1]. p. 1471–87. Available from: https://link.springer.com/article/10.1007/s12671-017-0742-x

[120] Desrosiers A, Vine V, Klemanski DH, Nolen-Hoeksema S. Mindfulness and emotion regulation in depression and anxiety: Common and distinct mechanisms of action. Depression and Anxiety [Internet]. 2013 Jul 1 [cited 2021 Jun 1];30(7):654–61. Available from: https://onlinelibrary.wiley.com/doi/full/10.1002/da.22124 23592556 10.1002/da.22124 PMC4012253

[121] Fales CL, Barch DM, Rundle MM, Mintun MA, Snyder AZ, Cohen JD, et al. Altered Emotional Interference Processing in Affective and Cognitive-Control Brain Circuitry in Major Depression. Biological Psychiatry. 2008 Feb 15;63(4):377–84. doi: 10.1016/j.biopsych.2007.06.012 17719567 PMC2268639

[122] Paulus MP. Cognitive control in depression and anxiety: Out of control? Vol. 1, Current Opinion in Behavioral Sciences. Elsevier Ltd; 2015. p. 113–20.

[123] Kashdan TB, Rottenberg J. Psychological flexibility as a fundamental aspect of health. Vol. 30, Clinical Psychology Review. Elsevier Inc.; 2010. p. 865–78. doi: 10.1016/j.cpr.2010.03.001 21151705 PMC2998793

[124] McCracken LM, Badinlou F, Buhrman M, Brocki KC. The role of psychological flexibility in the context of COVID-19: Associations with depression, anxiety, and insomnia. Journal of Contextual Behavioral Science. 2021 Jan 1;19:28–35.

[125] Zhou X, Zhu H, Zhang B, Cai T. Perceived social support as moderator of perfectionism, depression, and anxiety in college students. Social Behavior and Personality. 2013;41(7):1141–52.

[126] Dar KA, Iqbal N, Mushtaq A. Intolerance of uncertainty, depression, and anxiety: Examining the indirect and moderating effects of worry. Asian Journal of Psychiatry. 2017 Oct 1;29:129–33. doi: 10.1016/j.ajp.2017.04.017 29061409

[127] Russell JA, Weiss A, Mendelsohn GA. Affect Grid: A Single-Item Scale of Pleasure and Arousal. Journal of Personality and Social Psychology [Internet]. 1989 [cited 2021 Jun 1];57(3):493–502. Available from: https://psycnet.apa.org/journals/psp/57/3/493

[128] Hoffmann F, Banzhaf C, Kanske P, Bermpohl F, Singer T. Where the depressed mind wanders: Self-generated thought patterns as assessed through experience sampling as a state marker of depression. Journal of Affective Disorders. 2016 Jul 1;198:127–34. doi: 10.1016/j.jad.2016.03.005 27015160

[129] Naragon-Gainey K. Affective models of depression and anxiety: Extension to within-person processes in daily life. Journal of Affective Disorders. 2019 Jan 15;243:241–8. doi: 10.1016/j.jad.2018.09.061 30248635

[130] Nolen-Hoeksema S, Wisco BE, Lyubomirsky S. Rethinking Rumination. Perspectives on Psychological Science [Internet]. 2008 Sep 1 [cited 2021 Jun 1];3(5):400–24. Available from: https://journals.sagepub.com/doi/full/10.1111/j.1745-6924.2008.00088.x 26158958 10.1111/j.1745-6924.2008.00088.x

[131] Smallwood J, O’Connor RC. Imprisoned by the past: Unhappy moods lead to a retrospective bias to mind wandering. Cognition and Emotion [Internet]. 2011 [cited 2021 Jun 1];25(8):1481–90. Available from: https://www.tandfonline.com/action/journalInformation?journalCode=pcem20 21432633 10.1080/02699931.2010.545263

[132] Everaert J, Duyck W, Koster EHW. Attention, interpretation, and memory biases in subclinical depression: A proof-of-principle test of the combined cognitive biases hypothesis. Emotion [Internet]. 2014 [cited 2021 Jun 1];14(2):331–40. Available from: 10.1037/cap0000254 24512247

[133] Koster EHW, Crombez G, Verschuere B, de Houwer J. Attention to threat in anxiety-prone individuals: Mechanisms underlying attentional bias. Cognitive Therapy and Research [Internet]. 2006 Oct 10 [cited 2021 Jun 1];30(5):635–43. Available from: https://link.springer.com/article/10.1007/s10608-006-9042-9

[134] Mennen AC, Norman KA, Turk-Browne NB. Attentional bias in depression: understanding mechanisms to improve training and treatment. Vol. 29, Current Opinion in Psychology. Elsevier B.V.; 2019. p. 266–73. doi: 10.1016/j.copsyc.2019.07.036 31521030 PMC6980447

[135] Sanchez-Lopez A, de Raedt R, van Put J, Koster EHW. A novel process-based approach to improve resilience: Effects of computerized mouse-based (gaze)contingent attention training (MCAT)on reappraisal and rumination. Behaviour Research and Therapy. 2019 Jul 1;118:110–20. doi: 10.1016/j.brat.2019.04.005 31048096

[136] Connor KM, Davidson JRT. Development of a new Resilience scale: The Connor-Davidson Resilience scale (CD-RISC). Depression and Anxiety [Internet]. 2003 Sep 1 [cited 2021 Jun 2];18(2):76–82. Available from: www.interscience.wiley.com 12964174 10.1002/da.10113

[137] Smith BW, Dalen J, Wiggins K, Tooley E, Christopher P, Bernard J. The brief resilience scale: Assessing the ability to bounce back. International Journal of Behavioral Medicine [Internet]. 2008 Jul [cited 2021 Jun 1];15(3):194–200. Available from: https://link.springer.com/article/10.1080/10705500802222972 18696313 10.1080/10705500802222972

[138] Chen S, Bonanno GA. Psychological Adjustment During the Global Outbreak of COVID-19: A Resilience Perspective. Psychological Trauma: Theory, Research, Practice, and Policy [Internet]. 2020 [cited 2021 Jun 1]; Available from: https://psycnet.apa.org/journals/tra/12/S1/S51 32538658 10.1037/tra0000685

[139] Silberstein LR, Tirch D, Leahy RL, McGinn L. Mindfulness, psychological flexibility and emotional schemas. International Journal of Cognitive Therapy [Internet]. 2012 Dec 4 [cited 2021 Jun 1];5(4):406–19. Available from: https://guilfordjournals.com/doi/abs/10.1521/ijct.2012.5.4.406

[140] Friborg O, Hjemdal O, Rosenvinge JH, Martinussen M. A new rating scale for adult resilience: What are the central protective resources behind healthy adjustment? International Journal of Methods in Psychiatric Research [Internet]. 2003 Jun 1 [cited 2021 Jun 1];12(2):65–76. Available from: https://onlinelibrary.wiley.com/doi/full/10.1002/mpr.143 12830300 10.1002/mpr.143 PMC6878238

[141] Russo SJ, Murrough JW, Han MH, Charney DS, Nestler EJ. Neurobiology of resilience [Internet]. Vol. 15, Nature Neuroscience. Nature Publishing Group; 2012 [cited 2021 Jun 1]. p. 1475–84. Available from: https://www.nature.com/articles/nn.3234 23064380 10.1038/nn.3234 PMC3580862

[142] Ehret AM, Joormann J, Berking M. Examining risk and resilience factors for depression: The role of self-criticism and self-compassion. Cognition and Emotion [Internet]. 2015 [cited 2021 Jun 1];29(8):1496–504. Available from: https://www.tandfonline.com/action/journalInformation?journalCode=pcem20 25517734 10.1080/02699931.2014.992394

[143] Trompetter HR, De Kleine Elian •, Bohlmeijer ET. Why Does Positive Mental Health Buffer Against Psychopathology? An Exploratory Study on Self-Compassion as a Resilience Mechanism and Adaptive Emotion Regulation Strategy. Cognitive Therapy and Research. 2016;41. 10.1007/s10608-016-9774-0 PMC5410199 28515539

[144] Pidgeon AM, Keye M. Relationship between resilience, mindfulness, and pyschological well-being in university students. International Journal of Liberal Arts and Social Science. 2014;2(5):27–32.

[145] Tugade MM, Fredrickson BL. Regulation of positive emotions: Emotion regulation strategies that promote resilience. Journal of Happiness Studies [Internet]. 2007 Sep 19 [cited 2021 Jun 1];8(3):311–33. Available from: https://link.springer.com/article/10.1007/s10902-006-9015-4

[146] Hoorelbeke K, van den Bergh N, Wichers M, Koster EHW. Between vulnerability and resilience: A network analysis of fluctuations in cognitive risk and protective factors following remission from depression. Behaviour Research and Therapy. 2019 May 1;116:1–9. doi: 10.1016/j.brat.2019.01.007 30710666

[147] Lesh T v. Zen Meditation and the Development of Empathy in Counselors. In: Shapiro DH, Walsh RN, editors. Meditation Classic and Contemporary Perspectives. Routledge; 2017.

[148] Keefe T. Empathy: the critical skill. Social Work. 1976 Jan;21(1):10–4.

[149] Shapiro SL, Schwartz GE, Bonner G. Effects of mindfulness-based stress reduction on medical and premedical students. Journal of Behavioral Medicine [Internet]. 1998 [cited 2021 Jun 2];21(6):581–99. Available from: https://link.springer.com/article/10.1023/A:1018700829825 9891256 10.1023/a:1018700829825

[150] Dekeyser M, Raes F, Leijssen M, Leysen S, Dewulf D. Mindfulness skills and interpersonal behaviour. Personality and Individual Differences. 2008 Apr 1;44(5):1235–45.

[151] Winning AP, Boag S. Does brief mindfulness training increase empathy? The role of personality. Personality and Individual Differences. 2015 Nov 1;86:492–8.

[152] Kanske P, Böckler A, Trautwein FM, Singer T. Dissecting the social brain: Introducing the EmpaToM to reveal distinct neural networks and brain-behavior relations for empathy and Theory of Mind. NeuroImage. 2015 Nov 5;122:6–19. doi: 10.1016/j.neuroimage.2015.07.082 26254589

[153] Raab K. Mindfulness, Self-Compassion, and Empathy Among Health Care Professionals: A Review of the Literature. Journal of Health Care Chaplaincy [Internet]. 2014 [cited 2021 Jun 2];20(3):95–108. Available from: https://www.tandfonline.com/doi/abs/10.1080/08854726.2014.913876 doi: 10.1080/08854726.2014.913876 24926896

[154] Klimecki OM, Leiberg S, Lamm C, Singer T. Functional neural plasticity and associated changes in positive affect after compassion training. Cerebral Cortex [Internet]. 2013 Jul 1 [cited 2021 Jun 1];23(7):1552–61. Available from: https://academic.oup.com/cercor/article/23/7/1552/288473 22661409 10.1093/cercor/bhs142

[155] Klimecki OM, Leiberg S, Ricard M, Singer T. Differential pattern of functional brain plasticity after compassion and empathy training. Social Cognitive and Affective Neuroscience [Internet]. 2013 Jun 1 [cited 2021 Jun 1];9(6):873–9. Available from: https://academic.oup.com/scan/article/9/6/873/1669505 23576808 10.1093/scan/nst060 PMC4040103

[156] Singer T, Klimecki OM. Empathy and compassion. Vol. 24, Current Biology. Cell Press; 2014. p. R875–8. 10.1016/j.cub.2014.06.054 25247366

[157] de Vignemont F, Singer T. The empathic brain: how, when and why? Trends in Cognitive Sciences. 2006 Oct 1;10(10):435–41. doi: 10.1016/j.tics.2006.08.008 16949331

[158] Klimecki O, Singer T. Empathic distress fatigue rather than compassion fatigue? Integrating findings from empathy research in psychology and social neuroscience. In: Oakley B, Knafo A, Madhavan G, Wilson DS, editors. Pathological altruism. USA: Oxford University Press; 2012. p. 368–83.

[159] Beddoe AE, Murphy SO. Does mindfulness decrease stress and foster empathy among nursing students? Journal of Nursing Education. 2004;43(7):305–12. doi: 10.3928/01484834-20040701-07 15303583

[160] Engen HG, Singer T. Compassion-based emotion regulation up-regulates experienced positive affect and associated neural networks. Social Cognitive and Affective Neuroscience [Internet]. 2014 Oct 21 [cited 2021 Jun 2];10(9):1291–301. Available from: http://mrtools.mgh.harvard.edu/10.1093/scan/nsv008 PMC4560943 25698699

[161] Lamm C, Singer T. The role of anterior insular cortex in social emotions. [Internet]. Vol. 214, Brain structure & function. Springer; 2010 [cited 2021 Jun 1]. p. 579–91. Available from: https://link.springer.com/article/10.1007/s00429-010-0251-3 20428887 10.1007/s00429-010-0251-3

[162] Silani G, Bird G, Brindley R, Singer T, Frith C, Frith U. Levels of emotional awareness and autism: An fMRI study. Social Neuroscience [Internet]. 2008 [cited 2021 Jun 1];3(2):97–112. Available from: https://www.tandfonline.com/action/journalInformation?journalCode=psns20 18633852 10.1080/17470910701577020

[163] Bird G, Viding E. The self to other model of empathy: Providing a new framework for understanding empathy impairments in psychopathy, autism, and alexithymia. Vol. 47, Neuroscience and Biobehavioral Reviews. Elsevier Ltd; 2014. p. 520–32. 10.1016/j.neubiorev.2014.09.021 25454356

[164] Herbert BM, Herbert C, Pollatos O. On the relationship between interoceptive awareness and alexithymia: Is interoceptive awareness related to emotional awareness? Journal of Personality [Internet]. 2011 Oct 1 [cited 2021 Jun 1];79(5):1149–75. Available from: https://onlinelibrary.wiley.com/doi/full/10.1111/j.1467-6494.2011.00717.x 21241306 10.1111/j.1467-6494.2011.00717.x

[165] Galante J, Galante I, Bekkers MJ, Gallacher J. Effect of kindness-based meditation on health and well-being: A systematic review and meta-analysis. Journal of Consulting and Clinical Psychology [Internet]. 2014 [cited 2021 Jun 2];82(6):1101–14. Available from: https://psycnet.apa.org/journals/ccp/82/6/1101 24979314 10.1037/a0037249

[166] Leiberg S, Klimecki O, Singer T. Short-term compassion training increases prosocial behavior in a newly developed prosocial game. PLoS ONE [Internet]. 2011 [cited 2021 Jun 2];6(3):17798. Available from: http://www.nccr-neuro.ethz.ch 10.1371/journal.pone.0017798 PMC3052380 21408020

[167] Klimecki OM, Mayer S v., Jusyte A, Scheeff J, Schönenberg M. Empathy promotes altruistic behavior in economic interactions. Scientific Reports [Internet]. 2016 Aug 31 [cited 2021 Jun 2];6(1):1–5. Available from: www.nature.com/scientificreports 27578563 10.1038/srep31961 PMC5005993

[168] Weng HY, Schuyler B, Davidson RJ. The Impact of Compassion Meditation Training on the Brain and Prosocial Behavior. In: Seppälä Emma M., Simon-Thomas Emiliana, Brown Stephanie L., Worline Monica C., Cameron C. Daryl, Doty James R., editors. The Oxford handbook of compassion science. Oxford University Press; 2017. p. 133–46.

[169] Singer T, Lamm C. The Social Neuroscience of Empathy The ReSource Project (www.resource-project.org) View project Understanding endogenous emotions View project. 2009 [cited 2021 Jun 1]; https://www.researchgate.net/publication/281218239

[170] Klimecki OM. The Role of Empathy and Compassion in Conflict Resolution. Emotion Review [Internet]. 2019 Oct 1 [cited 2021 Jun 1];11(4):310–25. Available from: 10.1177/1754073919838609

[171] Klengel T, Binder EB. Epigenetics of Stress-Related Psychiatric Disorders and Gene × Environment Interactions. Vol. 86, Neuron. Cell Press; 2015. p. 1343–57. doi: 10.1016/j.neuron.2015.05.036 26087162

[172] Aristizabal MJ, Anreiter I, Halldorsdottir T, Odgers CL, McDade TW, Goldenberg A, et al. Biological embedding of experience: A primer on epigenetics [Internet]. Vol. 117, Proceedings of the National Academy of Sciences of the United States of America. National Academy of Sciences; 2020 [cited 2021 Jun 1]. p. 23261–9. Available from: www.pnas.org/cgi/doi/10.1073/pnas.1820838116 31624126 10.1073/pnas.1820838116 PMC7519272

[173] Zannas AS, Arloth J, Carrillo-Roa T, Iurato S, Röh S, Ressler KJ, et al. Lifetime stress accelerates epigenetic aging in an urban, African American cohort: Relevance of glucocorticoid signaling. Genome Biology [Internet]. 2015 Dec 17 [cited 2021 Jun 2];16(1):1–12. Available from: https://link.springer.com/articles/10.1186/s13059-015-0828-5 26673150 10.1186/s13059-015-0828-5 PMC4699359

[174] Czamara D, Eraslan G, Page CM, Lahti J, Lahti-Pulkkinen M, Hämäläinen E, et al. Integrated analysis of environmental and genetic influences on cord blood DNA methylation in new-borns. Nature Communications [Internet]. 2019 Dec 1 [cited 2021 Jun 1];10(1):1–18. Available from: 10.1038/s41467-019-10461-0 31186427 PMC6559955

[175] Schiele MA, Gottschalk MG, Domschke K. The applied implications of epigenetics in anxiety, affective and stress-related disorders—A review and synthesis on psychosocial stress, psychotherapy and prevention. Clinical Psychology Review. 2020 Apr 1;77:101830. doi: 10.1016/j.cpr.2020.101830 32163803

[176] Alladi CG, Etain B, Bellivier F, Marie-Claire C. Dna methylation as a biomarker of treatment response variability in serious mental illnesses: A systematic review focused on bipolar disorder, schizophrenia, and major depressive disorder [Internet]. Vol. 19, International Journal of Molecular Sciences. MDPI AG; 2018 [cited 2021 Jun 1]. p. 3026. Available from: www.mdpi.com/journal/ijms 10.3390/ijms19103026 PMC6213157 30287754

[177] Vinkers CH, Geuze E, van Rooij SJH, Kennis M, Schür RR, Nispeling DM, et al. Successful treatment of post-traumatic stress disorder reverses DNA methylation marks. Molecular Psychiatry [Internet]. 2021 Apr 1 [cited 2021 Jun 1];26(4):1264–71. Available from: https://www.nature.com/articles/s41380-019-0549-3 31645664 10.1038/s41380-019-0549-3

[178] Liu YZ, Wang YX, Jiang CL. Inflammation: The common pathway of stress-related diseases [Internet]. Vol. 11, Frontiers in Human Neuroscience. Frontiers Media S. A; 2017 [cited 2021 Jun 2]. p. 316. Available from: www.frontiersin.org 28676747 10.3389/fnhum.2017.00316 PMC5476783

[179] Rohleder N. Stimulation of systemic low-grade inflammation by psychosocial stress [Internet]. Vol. 76, Psychosomatic Medicine. Lippincott Williams and Wilkins; 2014 [cited 2021 Jun 1]. p. 181–9. Available from: https://journals.lww.com/psychosomaticmedicine/Fulltext/2014/04000/Stimulation_of_Systemic_Low_Grade_Inflammation_by.8.aspx 24608036 10.1097/PSY.0000000000000049

[180] Bierhaus A, Wolf J, Andrassy M, Rohleder N, Humpert PM, Petrov D, et al. A mechanism converting psychosocial stress into mononuclear cell activation. Proceedings of the National Academy of Sciences of the United States of America [Internet]. 2003 Feb 18 [cited 2021 Jun 1];100(4):1920–5. Available from: www.pnas.orgcgidoi10.1073pnas.0438019100 12578963 10.1073/pnas.0438019100 PMC149934

[181] Pace TWW, Mletzko TC, Alagbe O, Musselman DL, Nemeroff CB, Miller AH, et al. Increased stress-induced inflammatory responses in male patients with major depression and increased early life stress. American Journal of Psychiatry. 2006;163(9):1630–3. doi: 10.1176/ajp.2006.163.9.1630 16946190

[182] Zannas AS, Jia M, Hafner K, Baumert J, Wiechmann T, Pape JC, et al. Epigenetic upregulation of FKBP5 by aging and stress contributes to NF-κB-driven inflammation and cardiovascular risk. Proceedings of the National Academy of Sciences of the United States of America [Internet]. 2019 Jun 4 [cited 2021 Jun 2];166(23):11370–9. Available from: https://www.pnas.org/content/116/23/11370 31113877 10.1073/pnas.1816847116 PMC6561294

[183] Miller GE, Chen E, Sze J, Marin T, Arevalo JMG, Doll R, et al. A Functional Genomic Fingerprint of Chronic Stress in Humans: Blunted Glucocorticoid and Increased NF-κB Signaling. Biological Psychiatry. 2008 Aug 15;64(4):266–72. doi: 10.1016/j.biopsych.2008.03.017 18440494 PMC2581622

[184] Perrin AJ, Horowitz MA, Roelofs J, Zunszain PA, Pariante CM. Glucocorticoid resistance: Is it a requisite for increased cytokine production in depression? A systematic review and meta-analysis. [Internet]. Vol. 10, Frontiers in Psychiatry. Frontiers Media S.A.; 2019 [cited 2021 Jun 1]. p. 423. Available from: www.frontiersin.org 31316402 10.3389/fpsyt.2019.00423 PMC6609575

[185] Baumeister D, Akhtar R, Ciufolini S, Pariante CM, Mondelli V. Childhood trauma and adulthood inflammation: A meta-analysis of peripheral C-reactive protein, interleukin-6 and tumour necrosis factor-α. Molecular Psychiatry [Internet]. 2016 May 1 [cited 2021 Jun 1];21(5):642–9. Available from: www.nature.com/mp 26033244 10.1038/mp.2015.67 PMC4564950

[186] Miller AH, Maletic V, Raison CL. Inflammation and Its Discontents: The Role of Cytokines in the Pathophysiology of Major Depression. Vol. 65, Biological Psychiatry. Elsevier; 2009. p. 732–41. doi: 10.1016/j.biopsych.2008.11.029 19150053 PMC2680424

[187] Miller AH, Raison CL. Are anti-inflammatory therapies viable treatments for psychiatric disorders? Where the rubber meets the road [Internet]. Vol. 72, JAMA Psychiatry. American Medical Association; 2015 [cited 2021 Jun 1]. p. 527–8. Available from: https://jamanetwork.com/journals/jamapsychiatry/fullarticle/2212255 10.1001/jamapsychiatry.2015.22 PMC5542670 25853989

[188] Shields GS, Skwara AC, King BG, Zanesco AP, Dhabhar FS, Saron CD. Deconstructing the effects of concentration meditation practice on interference control: The roles of controlled attention and inflammatory activity. Brain, Behavior, and Immunity. 2020 Oct 1;89:256–67. doi: 10.1016/j.bbi.2020.06.034 32640286

[189] Bagby RM, Parker JDA, Taylor GJ. The twenty-item Toronto Alexithymia scale-I. Item selection and cross-validation of the factor structure. Journal of Psychosomatic Research. 1994 Jan 1;38(1):23–32. doi: 10.1016/0022-3999(94)90005-1 8126686

[190] Kroenke K, Spitzer RL, Williams JBW. The PHQ-9: Validity of a brief depression severity measure. Journal of General Internal Medicine [Internet]. 2001 Sep 1 [cited 2021 Jun 2];16(9):606–13. Available from: https://onlinelibrary.wiley.com/doi/full/10.1046/j.1525-1497.2001.016009606.x 11556941 10.1046/j.1525-1497.2001.016009606.x PMC1495268

[191] Spitzer RL, Kroenke K, Williams JBW, Löwe B. A brief measure for assessing generalized anxiety disorder: The GAD-7. Archives of Internal Medicine [Internet]. 2006 May 22 [cited 2021 Jun 2];166(10):1092–7. Available from: https://jamanetwork.com/ 16717171 10.1001/archinte.166.10.1092

[192] Rek K, Thielmann I, Henkel M, Crawford M, Piccirilli L, Graff A, et al. A psychometric evaluation of the standardized assessment of severity of personality disorder (SASPD) in nonclinical and clinical german samples. Psychological Assessment [Internet]. 2020 Oct 1 [cited 2021 Jun 1];32(10):984–90. Available from: https://psycnet.apa.org/journals/pas/32/10/984 32730074 10.1037/pas0000926

[193] Wittchen HU, Höfler M, Gander F, Pfister H, Storz S, Ustun B, et al. Screening for mental disorders: Performance of the Composite International Diagnostic Screener (CID-S). International Journal of Methods in Psychiatric Research [Internet]. 1999 Jun 1 [cited 2021 Jun 1];8(2):59–70. Available from: https://onlinelibrary.wiley.com/doi/full/10.1002/mpr.57

[194] Faul F, Erdfelder E, Lang AG, Buchner A. G*Power 3: A flexible statistical power analysis program for the social, behavioral, and biomedical sciences. In: Behavior Research Methods [Internet]. Psychonomic Society Inc.; 2007 [cited 2021 Jun 2]. p. 175–91. Available from: https://link.springer.com/article/10.3758/BF03193146 10.3758/bf03193146 17695343

[195] Pruessner JC, Wolf OT, Hellhammer DH, Buske-Kirschbaum A, von Auer K, Jobst S, et al. Free cortisol levels after awakening: A reliable biological marker for the assessment of adrenocortical activity. Life Sciences. 1997 Nov 21;61(26):2539–49. doi: 10.1016/s0024-3205(97)01008-4 9416776

[196] Cohen S, Kamarck T, Mermelstein R. A global measure of perceived stress. Journal of health and social behavior. 1983;24(4):385–96. 6668417

[197] Russell D, Peplau LA, Cutrona CE. The revised UCLA Loneliness Scale: Concurrent and discriminant validity evidence. Journal of Personality and Social Psychology [Internet]. 1980 [cited 2021 Jun 2];39(3):472–80. Available from: https://psycnet.apa.org/journals/psp/39/3/472 7431205 10.1037//0022-3514.39.3.472

[198] Garnefski N, Kraaij V, Spinhoven P. Negative life events, cognitive emotion regulation and emotional problems. Personality and Individual Differences. 2001 Jun 1;30(8):1311–27.

[199] Kaufman EA, Xia M, Fosco G, Yaptangco M, Skidmore CR, Crowell SE. The Difficulties in Emotion Regulation Scale Short Form (DERS-SF): Validation and Replication in Adolescent and Adult Samples. Journal of Psychopathology and Behavioral Assessment [Internet]. 2016 Sep 1 [cited 2021 Jun 2];38(3):443–55. Available from: https://link.springer.com/article/10.1007/s10862-015-9529-3 10.1007/s10862-015-9529-3 PMC12788809 41522882

[200] Caprara GV, Steca P, Zelli A, Capanna C. A New Scale for Measuring Adults’ Prosocialness. European Journal of Psychological Assessment. 2005;21(2).

[201] Mehling WE, Acree M, Stewart A, Silas J, Jones A. The multidimensional assessment of interoceptive awareness, version 2 (MAIA-2). PLoS ONE [Internet]. 2018 Dec 1 [cited 2021 Jun 2];13(12):e0208034. Available from: 10.1371/journal.pone.0208034 30513087 PMC6279042

[202] Gilbert P, McEwan K, Gibbons L, Chotai S, Duarte J, Matos M. Fears of compassion and happiness in relation to alexithymia, mindfulness, and self-criticism. Psychology and Psychotherapy: Theory, Research and Practice [Internet]. 2012 Dec 1 [cited 2021 Jun 2];85(4):374–90. Available from: www.wileyonlinelibrary.com 10.1111/j.2044-8341.2011.02046.x 23080529

[203] Gu J, Baer R, Cavanagh K, Kuyken W, Strauss C. Development and Psychometric Properties of the Sussex-Oxford Compassion Scales (SOCS). Assessment [Internet]. 2020 Jan 1 [cited 2021 Jun 2];27(1):3–20. Available from: 10.1177/1073191119860911 31353931 PMC6906538

[204] Neff KD, Tóth-Király I, Knox MC, Kuchar A, Davidson O. The Development and Validation of the State Self-Compassion Scale (Long- and Short Form). Mindfulness [Internet]. 2021 Jan 1 [cited 2021 Jun 2];12(1):121–40. Available from: https://link.springer.com/article/10.1007/s12671-020-01505-4

[205] Paulus C, Erziehungswissenschaft FR. DER SAARBRÜCKER PERSÖNLICHKEITSFRAGEBOGEN SPF (IRI) ZUR MESSUNG VON EMPATHIE [Internet]. 2009 [cited 2021 Jun 1]. http://psydok.psycharchives.de/jspui/handle/20.500.11780/3343

[206] Bradley KA, Debenedetti AF, Volk RJ, Williams EC, Frank D, Kivlahan DR. AUDIT-C as a brief screen for alcohol misuse in primary care. Alcoholism: Clinical and Experimental Research [Internet]. 2007 Jul 1 [cited 2021 Jun 2];31(7):1208–17. Available from: https://onlinelibrary.wiley.com/doi/full/10.1111/j.1530-0277.2007.00403.x 17451397 10.1111/j.1530-0277.2007.00403.x

[207] Murphy RO, Ackermann KA, Handgraaf MJJ. Measuring Social Value Orientation. Judgment and Decision Making [Internet]. 2011 Dec 1 [cited 2021 Jun 2];6(8):771–81. Available from: https://papers.ssrn.com/abstract=1804189

[208] Jones B, Rachlin H. Social discounting. Psychological Science [Internet]. 2006 Apr 6 [cited 2021 Jun 2];17(4):283–6. Available from: https://journals.sagepub.com/doi/full/10.1111/j.1467-9280.2006.01699.x 16623683 10.1111/j.1467-9280.2006.01699.x

[209] Smyth JM, Zawadzki MJ, Juth V, Sciamanna CN. Global life satisfaction predicts ambulatory affect, stress, and cortisol in daily life in working adults. Journal of Behavioral Medicine [Internet]. 2017 Apr 1 [cited 2021 Jun 2];40(2):320–31. Available from: https://link.springer.com/article/10.1007/s10865-016-9790-2 27600638 10.1007/s10865-016-9790-2

[210] Carver CS. You want to measure coping but your protocol’s too long: Consider the brief COPE. International Journal of Behavioral Medicine [Internet]. 1997 [cited 2021 Jun 2];4(1):92–100. Available from: https://link.springer.com/article/10.1207/s15327558ijbm0401_6 16250744 10.1207/s15327558ijbm0401_6

[211] Feldman G, Hayes A, Kumar S, Greeson J, Laurenceau J-P. Mindfulness and Emotion Regulation: The Development and Initial Validation of the Cognitive and Affective Mindfulness Scale-Revised (CAMS-R). Journal of Psychopathology and Behavioral Assessment. 2007;29(3).

[212] Tran US, Glück TM, Nader IW. Investigating the Five Facet Mindfulness Questionnaire (FFMQ): Construction of a Short Form and Evidence of a Two-Factor Higher Order Structure of Mindfulness. Journal of Clinical Psychology [Internet]. 2013 Sep 1 [cited 2021 Jun 1];69(9):951–65. Available from: https://onlinelibrary.wiley.com/doi/full/10.1002/jclp.21996 23784693 10.1002/jclp.21996

[213] Kertz SJ, Lee J, Björgvinsson T. Psychometric properties of abbreviated and ultra-brief versions of the penn state worry questionnaire. Psychological Assessment [Internet]. 2014 [cited 2021 Jun 2];26(4):1146–54. Available from: https://psycnet.apa.org/journals/pas/26/4/1146 24932640 10.1037/a0037251

[214] Gabrys RL, Tabri N, Anisman H, Matheson K. Cognitive control and flexibility in the context of stress and depressive symptoms: The cognitive control and flexibility questionnaire. Frontiers in Psychology [Internet]. 2018 Nov 19 [cited 2021 Jun 2];9(NOV):2219. Available from: www.frontiersin.org 30510530 10.3389/fpsyg.2018.02219 PMC6252356

[215] Bond FW, Hayes SC, Baer RA, Carpenter KM, Guenole N, Orcutt HK, et al. Preliminary Psychometric Properties of the Acceptance and Action Questionnaire-II: A Revised Measure of Psychological Inflexibility and Experiential Avoidance. Behavior Therapy. 2011 Dec 1;42(4):676–88. doi: 10.1016/j.beth.2011.03.007 22035996

[216] Aron A, Aron EN, Smollan D. Inclusion of Other in the Self Scale and the Structure of Interpersonal Closeness. Journal of Personality and Social Psychology [Internet]. 1992 [cited 2021 Jun 2];63(4):596–612. Available from: /fulltext/1993-03996-001.html

