# Supplementary material for: Investigating differential effects of socio-emotional and mindfulness-based online interventions on mental health, resilience and social capacities during the COVID-19 pandemic: The study protocol
Source: PLoS One. 2021 Nov 4;16(11):e0256323. doi: 10.1371/journal.pone.0256323 (PMC8568275; doi:10.1371/journal.pone.0256323)
Supplement: S1 File — (DOCX) [file pone.0256323.s001.docx]

| Titel der Studie | **CovSocial Phase 2 - investigating differential effects of mindfulness based online-interventions on mental wellbeing and social cohesion**  **CovSocial Phase 2 - Untersuchung differenzieller Effekte von achtsamkeitsbasierten Online-Interventionen auf psychisches Wohlbefinden und soziale Kohäsion** |
| --- | --- |
| 1. Entscheidungen anderer Ethikkommissionen in derselben Sache | entfällt |
| 2. Gegenstand der Studie und ihre Ziele; Angabe der Hypothesen, getrennt in Haupt- und Sekundärhypothesen sowie der klinischen Parameter (primäre und sekundäre Endpunkte), anhand derer die Hypothesen geprüft werden sollen | Ziel der geplanten Studie ist es, die Wirksamkeit von zwei achtsamkeitsbasierten Online-Interventionen auf die Reduktion von Stress und Einsamkeit sowie die Steigerung von psychischem Wohlbefinden, prosozialem Verhalten und Mitgefühl zu untersuchen und miteinander zu vergleichen. Die vorgelegte Studie dient hierbei als Phase 2 des CovSocial-Projekts, dessen Ziel es ist, die Veränderung von psychischer Gesundheit sowie sozialer Kohäsion über die Zeit der SARS-CoV-2-Pandemie in einer großen Stichprobe von Berliner Bürger*innen zu untersuchen. In Phase 1 (EA4/172/20) wurden hierzu Persönlichkeits- und Verhaltensmerkmale (Trait) identifiziert, die mit höherer Vulnerabilität für Stress und psychische Gesundheit oder verminderter Resilienz (State) während der SARS-CoV-2-Pandemie assoziiert sind. Zusätzlich wurden State-Maße zu den relevanten Faktoren für 7 Zeitpunkte (T1-T7) von Januar 2020 bis März 2021 erhoben. Auch sogenannte polygene Risikoscores (genetisch und epigenetisch) wurden in der ersten Phase des Projekts in einer Substichprobe erhoben, welche individuelle Bewältigungsmuster beeinflussen könnten (Wray et al., 2020). Diese spiegeln das aggregierte genetische Risiko für eine bestimmte Erkrankung (z.B. psychische Erkrankungen wie Depression oder Angststörungen etc.) wider oder korrelieren mit der Ausprägung bestimmter Phänotypen und Verhaltensmuster (z.B. Neurotizismus, Optimismus, Belohnungssystem etc.), die für die individuelle Ausprägung von Stress, Resilienz und Sozialer Kohäsion wichtig sein könnten. Die Berechnung dieser polygenen Risikoscores beruht auf Ergebnissen großen Studien, oft von internationalen Konsortien und großen Biobanken. Der prädiktive Wert dieser Scores nimmt mit der Größe der Kohorten und Metaanalysen zu und diese Scores müssen daher immer der aktuellen Literatur angepasst werden.  Phase 2 besteht aus der Erforschung von achtsamkeitsbasierten mentalen Interventionsprogrammen in einer Subgruppe der in Phase 1 untersuchten Proband*innen. Diese Interventionsprogramme wurden auf der Grundlage eines mentalen Trainingsprogramms, dem ReSource-Projekt (Singer et al., 2016), erstellt, welches diese mentale Interventionen als Elemente enthielt. Die Wirksamkeit auf Stressreduktion und eine Steigerung von prosozialem Verhalten dieses Programms wurde in zahlreichen Studien nachgewiesen (Hildebrandt et al, 2017; Singer & Engert, 2019). In der geplanten Interventionsstudie Phase 2 des CovSocial-Projekts sollen nun die mentalen Trainingsübungen ohne den Kontext anderer Interventionselemente erforscht werden. So soll die Skalierbarkeit kurzer Online-Interventionen untersucht werden.  Im Spezifischen sollen zwei verschiedene Interventionsgruppen miteinander sowie mit einer Retest-Kontrollgruppe anhand von Prä- und Posttestungen sowie mit täglichen oder wöchentlichen online-app-basierten ambulatorischen Messprotokoll („ecological momentary-assessment (EMA)“) Fragen verglichen werden. Die eine Trainingsgruppe durchläuft ein klassisches mehrwöchiges achtsamkeitsbasiertes Meditationsprogramm und die andere Gruppe ein gleichlanges auf kurze tägliche Partnerübungen beruhendes Dyadenprogramm (Kok & Singer, 2017). Beide mentalen Trainingsprogramme haben in vorheriger Forschung in ähnlicher Form Stress- und Einsamkeitsreduzierende Wirkungen sowie eine Steigerung in sozialen Kompetenzen und Prosozialität gezeigt (Singer & Engert, 2019). Als abhängige Variablen stehen im Fokus die Erfassung von Veränderungen im Stresserleben, in erlebter Einsamkeit, in Resilienz, in psychischer Gesundheit sowie sozialer Nähe, sozialer Kohäsion und sozialen Fähigkeiten wie (Selbst)Mitgefühl und Empathie. Zudem sollen durch längsschnittliche Daten aus Phase 1 sowohl Prä- als auch trainingsbedingte Effekte in Phase 2 vorhergesagt werden.  Die SARS-CoV-2-Pandemie stellt eine globale Herausforderung mit tiefgreifenden Auswirkungen auf Gesundheitssysteme, Wirtschaft und soziales Leben dar. Angesichts der staatlichen Einschränkungen, kombiniert mit der Angst, sich anzustecken, und dem Risiko, den Arbeitsplatz zu verlieren, wird die Situation mittlerweile als ein belastendes Lebensereignis für den Großteil der Bevölkerung angesehen (Horesh & Brown, 2020). Daraus entsteht die Notwendigkeit, psychische Folgen der Pandemie und des damit verbundenen Lockdowns zu untersuchen sowie Personen zu identifizieren, die das größte Risiko haben, nach dem Erleben von COVID-19-bedingtem Stress psychische Probleme zu entwickeln. Durch die Intervention sollen diese Personen dabei unterstützt werden, Stress zu reduzieren und ihr psychisches Wohlbefinden zu erhöhen. Zudem können zukünftig Personen mit einem erhöhten Risiko für eine hohe Stressbelastung frühzeitig identifiziert werden. So können schwere Stressreaktionen und das Auftreten von psychischen Störungen prospektiv verhindert werden.  Untersuchungen zu den psychischen Folgen von Pandemien haben gezeigt, dass insbesondere die Quarantäne- und Isolationsregelungen in einem Anstieg von Stress, Angst und Nervosität und Depressionen resultiert sind (Brooks et al., 2020). In einer Studie zur SARS-Pandemie 2002/2003 stellte die soziale Isolation den größten Faktor für spätere starke Stressreaktionen dar (Bai et al., 2004). Da insbesondere die wiederholten Phasen von Lockdowns in Berlin seit März 2020 ebenfalls vor allem durch den Appell zur Selbstisolation und Kontaktminimierung geprägt war, kann davon ausgegangen werden, dass auch hier viele Menschen in dieser Zeit von erhöhtem Stress betroffen waren. Weitere Belastungsfaktoren für Stress in der Pandemie sind unterschiedliche individuelle Herausforderungen wie Angst vor Arbeitslosigkeit, Sorge um die Gesundheit oder der erhöhte Kinderbetreuungsaufwand durch die Schließung von Schulen und Kindergärten. Aufgrund der sozialen Isolation, der hohen Ungewissheit, individueller Probleme sowie der potenziell schädigenden, bedrohenden oder herausfordernden Auswirkungen der COVID-19-Pandemie kann angenommen werden, dass infolge der Pandemie der Stress in der Bevölkerung stark angestiegen ist. Diese Vermutung wird durch erste Ergebnisse von Studien zu den Auswirkungen der COVID-19-Pandemie auf die psychische Gesundheit bestätigt. In Deutschland wurde eine Zunahme von Angststörungssymptomen, Depressionen und psychologischem Stress festgestellt (Bäuerle et al., 2020). Dieser Anstieg wurde vor allem durch das Ausmaß der Einschränkung von sozialen Kontakten und die Schwere der Veränderung des Alltags vorhergesagt (Benke et al., 2020).  Stressbelastung geht mit einer Aktivierung stress-bezogener biologischer Regulationssysteme, wie der Hypothalamus-Hypophysen-Nebennierenrinden-Achse (HHNA) und dem sympatho-adrenergen System einher. Ist die Stressbelastung chronisch, wie während der anhaltenden Covid-Pandemie, kann die dauerhafte Aktivierung dieser biologischen Stress-Achsen zu Veränderungen in neuroendokrinen, immunologischen, metabolischen Regelkreisen, und zellulären Alterungsprozessen führen, die wiederum dem erhöhten Risiko für körperliche und psychische Erkrankungen bei chronischer Stressbelastung zugrunde liegen (Cohen et al., 2012). Es ist bekannt, dass lebensgeschichtlich frühe Stresserfahrungen das Risiko für stressbezogene Erkrankungen erhöhen. Stress während sensibler Phasen der Entwicklung führt zu einer biologischen Einbettung auf epigenetischer Ebene sowie Änderungen in neuralen und peripheren Regulationssystemen, welche ultimativ zum Krankheitsrisiko beitragen, vor allem nach erneuter Stressbelastung im späteren Leben (Vulnerabilitätsmodell; Heim et al., 2019). Ein weiteres Ziel dieser Studie ist es daher, den Zusammenhang zwischen chronischer Stressbelastung während der COVID-19-Pandemie und Veränderungen in stress-relevanten biologischen Systemen zu untersuchen, sowie Faktoren zu identifizieren, die die Vulnerabilität für Veränderungen stress-assoziierter biologischer Systeme während der COVID-19-Pandemie erhöhen.  Diese biologischen Veränderungen können zudem im Laufe der Zeit über die Transkriptionsfaktoraktivierung eingebettet werden, indem die Genregulation und -expression durch Veränderungen im Epigenom verändert wird (Klengel & Binder, 2015). Epigenetische Veränderungen verändern die Gentranskription nicht durch Veränderung der Genomsequenz, sondern durch Veränderung ihrer Zugänglichkeit für Transkriptionsregulatoren (z.B. DNA-Methylierung, Histonmodifikationen; Aristizabal et al., 2019). Im Falle von chronischem Stress werden Stresshormone oder Glukokortikoide, die als Transkriptionsfaktoren fungieren und dauerhafte epigenetische Veränderungen induzieren können, wahrscheinlich eine herausragende Rolle bei diesen Veränderungen spielen, wie die Überschneidung der epigenetischen Effekte von chronischem Stress und Glukokortikoiden zeigt sowie die Anreicherung der DNA-Methylierungsstelle der Glukokortikoid-Antwort bei stressbedingten epigenetischen Veränderungen (Zannas et al., 2015). Diese Umwelt-abhängigen epigenetischen Veränderungen werden durch genetische Faktoren beeinflusst, so dass Gene, Umwelt und Epigenetik zusammen untersucht werden sollten (Czamara et al., 2019). Epigenetische Marker können zudem sowohl Erfolge therapeutischer Interventionen vorhersagen als auch durch derartige Interventionen langfristig verändert werden (Schiele, Gottschalk & Domschke, 2020; Goud Alladi et al., 2018; Vinkers et al., 2019)  Für die Studie sollen Daten von insgesamt 300 Personen (100 Personen pro Gruppe) vor, während und nach der geplanten Intervention erhoben werden. Die Proband*innen werden mittels stratifizierter Randomisierung drei Gruppen zugeteilt **(Anlage L).** Die Proband*innen durchlaufen jeweils ein 10-wöchiges Interventionstraining. Gruppe I wird sechsmal pro Woche die 12-minütige Affektdyade durchführen (Kok & Singer, 2017). Diese tägliche app-basierte online Partnerübung besteht für die Person, die jeweils spricht (6 Minuten) aus einer nicht-urteilenden Beschreibung von stressvollen sowie mit Dankbarkeit geprägten Situationen während des letzten Tages und deren Effekt auf die Körperempfindung (interozeptives Körpergewahrsein). Die Person, die zuhört, (6 Minuten) trainiert jeweils nicht-urteilendes empathisches Zuhören. Nach der Hälfte der Zeit werden die Rollen gewechselt. Gruppe II wird sechsmal pro Woche eine 12-minütige Achtsamkeitsmeditation sowie die Atemmeditation durchführen. Gruppe III dient als Retest-Kontrollgruppe mit Wartelistenbedingung und wird nach der Post-Testung der drei Gruppen, wie vorher Gruppe I nun auch sechsmal pro Woche die 12-minütige Affektdyade durchführen. Den Gruppen I und II wird zudem angeboten, nach der Post-Testung die jeweils praktizierten achtsamkeitsbasierten Intervention per App weiter fortzuführen. Je nach Höhe der Drop-Out-Rate sowie Finanzierungsmöglichkeiten, wird auch eine Follow-Up-Messung der Gruppen I und II erfolgen. Eine graphische Darstellung des Studiendesigns ist Abbildung 1 zu entnehmen.  **Abbildung 1.** *Studienablauf der Phase 2 im CovSocial-Projekt.*  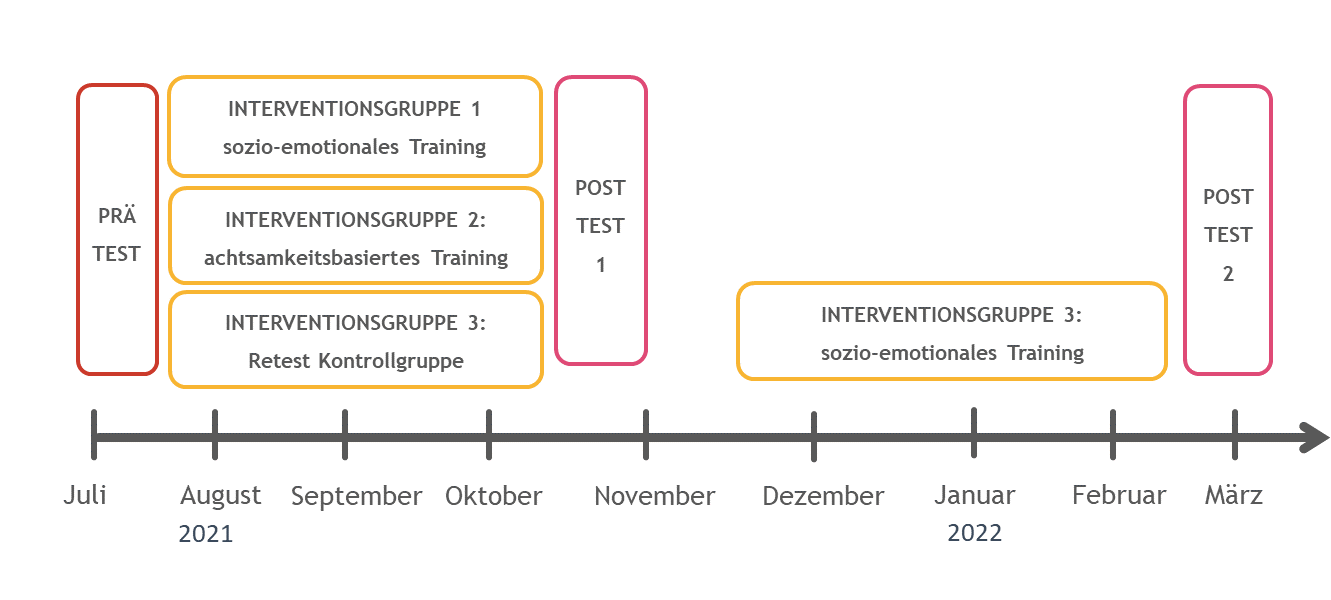  Psychologische, behaviorale und biologische Parameter werden von allen Proband*innen vor und nach der Intervention erhoben. Psychologische Daten werden mit Online-Fragebögen sowie einem ambulatorischen Messprotokoll („ecological momentary-assessment (EMA)“) erfasst. Dabei werden Daten in Echtzeit während des gewohnten Alltags der Proband*innen gesammelt. Biologische Marker betreffen neuroendokrine, immunologische, metabolische Parameter, sowie Indikatoren zellulärer Alterungsprozesse sowie genomweite epigenetische Marker. Sie werden durch eine Blutprobe vor der Intervention sowie Speichelproben vor und nach der Intervention erhoben. Behaviorale Daten werden mithilfe von computerbasierten Experimenten erhoben. Während der jeweiligen Intervention werden zudem weitere psychologische Daten über das Smartphone -basierte Verfahren der EMA erhoben.  Folgende Hypothesen werden getestet:  H1: Wir erwarten Interventionseffekte auf den erhobenen Stressmarkern aus Speichelproben, EMA-Messungen und Fragebögen  H1a: Wir erwarten für Individuen, die das sozio-emotionale Training oder das achtsamkeitsbasierte Training durchlaufen, im Vergleich zur Retest-Kontrollgruppe eine Reduktion in subjektiv erlebtem Stress, gemessen durch den PSS-10, beim Posttest im Vergleich zum Prätest.  H1b: Wir erwarten eine Reduktion der Cortisol Awakening Response (CAR) für Individuen, die das sozio-emotionale Training durchlaufen, im Vergleich zu jenen, die das achtsamkeitsbasierte Training durchlaufen und zur Test-Retest-Kontrollgruppe. Wir erwarten zudem, dass diese Reduktion durch einen Anstieg an Akzeptanz als Stressbewältigungsstrategien, gemessen durch den CERQ, mediiert wird.  H1c: Individuen, die eine höhere Ausprägung auf psychologischen Resilienzmarkern aufweisen (in Phase 1 des Projekts erhoben) sowie einem höheren Anstieg an subjektivem Stresserleben während der Pandemie berichten (in Phase 1 des Projekts erhoben) zeigen nach dem sozio-emotionalen oder nach dem achtsamkeitsbasierten Training eine stärkere Reduktion in subjektivem Stresserleben, gemessen mit dem PSS-10, als Individuen mit niedrigeren Werten auf psychologischen Resilienz-markern oder niedrigerem Anstieg an subjektivem Stresserleben während der Pandemie.  H1d: Individuen, die eine höhere Ausprägung auf psychologischen Resilienzmarkern aufweisen (in Phase 1 des Projekts erhoben) sowie einem höheren Anstieg an subjektivem Stresserleben während der Pandemie berichten (in Phase 1 des Projekts erhoben) zeigen nach dem sozio-emotionalen Training eine stärkere Reduktion in der Cortisol Awakening Response (CAR) als Individuen mit niedrigeren Werten auf psychologischen Resilienzmarkern oder niedrigerem Anstieg an subjektivem Stresserleben während der Pandemie.  H2: Wir erwarten Interventionseffekte auf subjektiv empfundene Einsamkeit.  H2a: Wir erwarten von Prätest zu Posttest eine Reduktion in subjektiv empfundener Einsamkeit, gemessen mit dem UCLA, für Individuen, die das sozio-emotionale Training durchlaufen, aber nicht für jene, die das achtsamkeitsbasierte Training durchlaufen oder der Test-Retest-Kontrollgruppe angehören.  H2b: Wir erwarten einen Anstieg an subjektiv empfundener sozialer Nähe für Individuen, die das sozio-emotionale Training durchlaufen, aber nicht für jene, die das achtsamkeitsbasierte Training durchlaufen. Weiterhin, erwarten wir einen Anstieg an geteilten persönlichen Informationen im Laufe des sozio- emotionalen Trainings.  H2c: Die Reduktion in subjektiv empfundener Einsamkeit geht mit einem Anstieg von subjektiv empfundener sozialer Nähe, gemessen mit dem IOS vor und nach dem sozio-emotionalen Training, sowie mit einem Anstieg an in der Dyade jeweils geteilten persönlichen Informationen (personal disclosure) einher.  H3: Wir erwarten Interventionseffekte auf die psychische Gesundheit.  H3a: Wir erwarten eine stärkere Reduktion in der Ausprägung subklinischer depressiver Symptomatik, gemessen mit dem BDI-II, für Individuen, die das sozio-emotionale Training durchlaufen, als für die, die das achtsamkeitsbasierte Training durchlaufen oder der Test-Retest-Kontrollgruppe angehören. Wir erwarten zudem eine stärkere Reduktion in der Ausprägung subklinischer depressiver Symptomatik für Individuen, die das achtsamkeitsbasierte Training durchlaufen als für jene, die der Test-Retest-Kontrollgruppe angehören.  H3b: Wir erwarten, dass eine Reduktion in der Ausprägung subklinischer depressiver Symptomatik, gemessen mit dem BDI-II, von Prätest zu Posttest bei Individuen, die das sozio-emotionale Training durchlaufen, mit einem vermehrten Gebrauch von Akzeptanz als Stressbewältigungsstrategie, gemessen mit dem CERQ, im Laufe der Intervention zusammenhängt, und bei Individuen, die das achtsamkeitsbasierte Training durchlaufen, mit einem geringeren Gebrauch von Rumination als Copingstrategie, gemessen mit dem CERQ, im Laufe der Intervention zusammenhängt.  H3c: Wir erwarten, dass eine größere Reduktion in der Ausprägung subklinischer Angstsymptome, gemessen mit dem STAI, von Prätest zu Posttest für Individuen, die das sozio-emotionale Training durchlaufen, als für die, die das achtsamkeitsbasierte Training durchlaufen oder der Retest-Kontrollgruppe angehören. Wir erwarten zudem eine stärkere Reduktion in der Ausprägung subklinischer Angstsymptome für Individuen, die das achtsamkeitsbasierte Training durchlaufen als für jene, die der Test-Retest-Kontrollgruppe angehören.  H3d: Wir erwarten, dass Werte auf psychologischen Resilienz- und Vulnerabilitätsmarkern (in Phase 1 des Projekts erhoben) sowohl auf Trait-Ebene als auch bezüglich deren Veränderung während der Pandemie in 2020 und Anfang 2021 hinweg die Stärke der trainingsbedingten Veränderungen in der Ausprägung subklinischer Angstsymptome (STAI) und depressiver Symptomatik (BDI) von Prätest zu Posttest vorhersagen können.  H4: Wir erwarten Interventionseffekte auf psychische Resilienz.  H4a: Wir erwarten einen stärkeren Anstieg an psychologischer Resilienz, gemessen mit der BRS, für Individuen, die das sozio-emotionale Training durchlaufen, als für die, die das achtsamkeitsbasierte Training durchlaufen oder der Retest-Kontrollgruppe angehören. Wir erwarten zudem einen stärkeren Anstieg an psychologischer Resilienz für Individuen, die das achtsamkeitsbasierte Training durchlaufen als für jene, die der Retest-Kontrollgruppe angehören.  H4b: Wir erwarten, dass der Anstieg an psychologischer Resilienz (BRS) von Prätest zu Posttest für Individuen, die das sozio-emotionale Training durchlaufen, mit einem mit einem vermehrten Gebrauch von Akzeptanz und sozialer Unterstützung als Copingstrategien, gemessen mit dem CERQ, sowie mit einem Anstieg an psychologischer Flexibilität, gemessen mit dem Affective Flexibility Task, im Laufe der Intervention zusammenhängt. Für Individuen, die das achtsamkeitsbasierte Training durchlaufen, erwarten wir, dass der Anstieg an psychologischer Resilienz von Prätest zu Posttest mit einer Reduktion von Rumination (CERQ) und einem Anstieg an Akzeptanz (CERQ) zusammenhängt.  H4c: Wir erwarten, dass Werte auf psychologischen Resilienz- und Vulnerabilitätsmarkern (in Phase 1 des Projekts erhoben) sowohl auf Trait-Ebene als auch bezüglich deren Veränderung während der Pandemie in 2020 und Anfang 2021 hinweg die Stärke der trainingsbedingten Veränderungen in psychologischer Resilienz von Prätest zu Posttest vorhersagen können.  H5: Wir erwarten Interventionseffekte auf Empathie und Mitgefühl.  H5a: Wir erwarten einen stärkeren Anstieg an Empathie und Mitgefühl, gemessen mit dem EmpaToM, dem IRI und dem SCS, für Individuen, die das sozio-emotionale Training durchlaufen, als für die, die das achtsamkeitsbasierte Training durchlaufen oder der Retest-Kontrollgruppe angehören. Wir erwarten zudem einen stärkeren Anstieg an Empathie, aber nicht in Mitgefühl, für Individuen, die das achtsamkeitsbasierte Training durchlaufen als für jene, die der Retest-Kontrollgruppe angehören. Wir erwarten keine Veränderungen von Empathie und Mitgefühl in der Test-Retest-Kontrollgruppe von Prätest zu Posttest. Zudem erwarten wir in keiner Gruppe Veränderung in Theory of Mind bzw. kognitiver Perspektivenübernahme.  H5b: Wir erwarten, dass ein Anstieg an Empathie in beiden Interventionsgruppen durch einen Anstieg an interozeptiver Wahrnehmung (gemessen mit EMA-Fragen vor und nach dem Training), Achtsamkeit (gemessen mit dem MAIA und CAMS-R) und einem vermehrten Gebrauch von Akzeptanz als Copingstrategie mediiert ist.  H5c: Wir erwarten, dass ein Anstieg an Mitgefühl von Prätest zu Posttest durch eine Reduktion von Angst vor Mitgefühl, einen Anstieg an Selbst-Mitgefühl (SCS Subskala self-kindness), eine Reduktion von persönlichem Distress (IRI Subskala Personal Distress), einen Anstieg an Akzeptanz (CERQ) und positive Gefühle (Affekt Grid) mediiert ist.  H5d: Auf Grund positiver Assoziationen zwischen sozialen Fähigkeiten und adaptiven Copingstrategien (der in Phase 1 des Projekts gefunden wurde), erwarten wir, dass der Gebrauch von adaptiven Copingstrategien, die in Phase 1 erhoben wurden, einen stärkeren trainingsbedingten Anstieg an Empathie und Mitgefühl vorhersagen.  H5e: Wir erwarten weiterhin, dass Werte auf psychologischen Sozialen Kohäsions- und Vulnerabilitätsmarkern (in Phase 1 des Projekts erhoben) sowohl auf Trait-Ebene als auch bezüglich deren Veränderung während der Pandemie in 2020 und Anfang 2021 hinweg die Stärke der trainingsbedingten Veränderungen in Empathie und Mitgefühl vorhersagen werden.  H6: Wir erwarten Interventionseffekte auf prosoziales Verhalten.  H6a: Wir erwarten einen stärkeren Anstieg an altruistischem prosozialem Verhalten, gemessen mit dem PSA und einer Batterie von Verhaltenstests für altruistisches prosoziales Verhalten (Böckler et al., 2018), für Individuen, die das sozio-emotionale Training durchlaufen, als für die, die das achtsamkeitsbasierte Training durchlaufen oder der Retest-Kontrollgruppe angehören. Wir erwarten keine Veränderungen von prosozialem Verhalten von Prätest zu Posttest für Individuen, die das achtsamkeitsbasierte Training durchlaufen oder der Retest-Kontrollgruppe angehören.  H6b: Wir erwarten, dass ein Anstieg an altruistischem prosozialem Verhalten durch den Anstieg an Empathie und Mitgefühl vorausgesagt werden kann. Weiterhin erwarten wir, dass eine Reduktion von Angst vor dem Ausdruck von Mitgefühl gegenüber anderen, gemessen mit der Fear of Compassion Scale, individuelle Unterschiede in trainingsbedingten Anstiegen in prosozialem Verhalten vorhersagen kann.  H6c: Wir erwarten weiterhin, dass Werte auf psychologischen Sozialen Kohäsions- sowie Vulnerabilitätsmarkern (in Phase 1 des Projekts erhoben) sowohl auf Trait-Ebene als auch bezüglich deren Veränderung während der Pandemie in 2020 und Anfang 2021 hinweg die Stärke der trainingsbedingten Veränderungen in Prosozialem Verhalten vorhersagen werden.  **Hypothesen zu stressbiologischen Systemen aus Blutproben**  H7a: Wahrgenommene Stressbelastung während der Pandemie (Phase 1) ist mit Aktivierung stressbiologischer Systeme assoziiert.  H7b: Dies geht mit immunologischen und metabolischen sowie Veränderungen in zellulären Alterungsprozessen einher.  H7c: Diese stress-assoziierten biologischen Marker mediieren den Zusammenhang zwischen subjektivem Stressempfinden während der Pandemie und psychischer und körperlicher Gesundheit zum Zeitpunkt des Prätests.  H8: Es bestehen interindividuelle Unterschiede bzgl. der Stärke des Zusammenhangs zwischen subjektiv erlebtem Stress (Phase 1) während der Pandemie und Veränderungen in stressbiologischen Systemen in Abhängigkeit von Stressbelastung während der Kindheit (in Phase 1 mit Childhood Trauma Questionnaire erhoben).  H8a: Individuen mit hoher frühkindlicher Stressbelastung (in Phase 1 mit Childhood Trauma Questionnaire erhoben) zeigen ein höheres Ausmaß der Dysregulation in stress-biologischen Parametern zum Zeitpunkt des Prätests.  H8b: Individuen mit hoher frühkindlicher Stressbelastung zeigen eine stärkere Beeinträchtigung der psychischen und körperlichen Gesundheit zum Zeitpunkt des Prätests.  H8c: Diese stress-assoziierten biologischen Marker mediieren den Zusammenhang zwischen hoher Stressbelastung während der Kindheit und psychischer und körperlicher Gesundheit zum Zeitpunkt des Prätests.  **Hypothesen zu genetischen und epi-genetischen Markern**  H9: Es soll ein Einfluss von polygenen und polyepigenetischen Risikoscores auf trainingsbedingte Interventionseffekte untersucht werden.  H9a: Es soll explorativ untersucht werden ob polygene Risikoscores, die in Phase 1 erhoben wurden, die unter den Hypothesen 1- 6 erwähnten Effekte der Intervention vorhersagen können.  H9b: Es soll untersucht werden ob polyepigenetische Risikoscores, die in Phase 1 erhoben wurden, die unter den Hypothesen 1- 6 erwähnten Effekte der Intervention vorhersagen können.  H10: Es soll untersucht werden, ob die sozio-emotionalen und achtsamkeitsbasierten Interventionen sich positiv auf die polyepigenetische Risikoscores im Posttest im Vergleich zum Prätest auswirken.  *Literatur:*  Bai, Y., Lin, C. C., Lin, C. Y., Chen, J. Y., Chue, C. M., & Chou, P. (2004). Survey of stress reactions among health care workers involved with the SARS outbreak. *Psychiatric Services*, *55*(9), 1055-1057.  Bäuerle, A., Teufel, M., Musche, V., Weismüller, B., Kohler, H., Hetkamp, M., ... & Skoda, E. M. (2020). Increased generalized anxiety, depression and distress during the COVID-19 pandemic: a cross-sectional study in Germany. *Journal of Public Health*, *42*(4), 672-678.  Benke, C., Autenrieth, L. K., Asselmann, E., & Pané-Farré, C. A. (2020). Lockdown, quarantine measures, and social distancing: Associations with depression, anxiety and distress at the beginning of the COVID-19 pandemic among adults from Germany. *Psychiatry research*, *293*, 113462.  Brooks, S. K., Webster, R. K., Smith, L. E., Woodland, L., Wessely, S., Greenberg, N., & Rubin, G. J. (2020). The psychological impact of quarantine and how to reduce it: rapid review of the evidence. *The lancet*, *395*(10227), 912-920.  Cohen, S., Janicki-Deverts, D., Doyle, W. J., Miller, G. E., Frank, E., Rabin, B. S., & Turner, R. B. (2012). Chronic stress, glucocorticoid receptor resistance, inflammation, and disease risk. *Proceedings of the National Academy of Sciences*, *109*(16), 5995-5999.  Czamara, D., Eraslan, G., Page, C.M., Lahti, J., Lahti-Pulkkinen, M., Hämäläinen, E., Kajantie, E., Laivuori, H., Villa, P.M., Reynolds, R.M., Nystad, W., Håberg, S.E., London, S.J., O'Donnell, K.J., Garg, E., Meaney, M.J., Entringer, S., Wadhwa, P.D., Buss, C., Jones, M.J., Lin, D.T.S., MacIsaac, J.L., Kobor, M.S., Koen, N., Zar, H.J., Koenen, K.C., Dalvie, S., Stein, D.J., Kondofersky, I., Müller, N.S., & Theis, F.J. (2019). Major Depressive Disorder Working Group of the Psychiatric Genomics Consortium, Räikkönen, K., Binder, E.B. Integrated analysis of environmental and genetic influences on cord blood DNA methylation in new-borns. Nature Communications, 10(1), 2548. doi: 10.1038/s41467-019-10461-0. PMID: 31186427; PMCID: PMC6559955.  Goud Alladi, C., Etain, B., Bellivier, F., & Marie-Claire, C. (2018). DNA methylation as a biomarker of treatment response variability in serious mental illnesses: a systematic review focused on bipolar disorder, schizophrenia, and major depressive disorder. International journal of molecular sciences, 19(10), 3026.  Heim, C. M., Entringer, S., & Buss, C. (2019). Translating basic research knowledge on the biological embedding of early-life stress into novel approaches for the developmental programming of lifelong health. Psychoneuroendocrinology, 105, 123-137.  Hildebrandt, L. K., McCall, C., & Singer, T. (2017). Differential effects of attention-, compassion-, and socio-cognitively based mental practices on self-reports of mindfulness and compassion. *Mindfulness, 8(6),* 1488-1512.  Horesh, D., & Brown, A. D. (2020). Traumatic stress in the age of COVID-19: A call to close critical gaps and adapt to new realities. *Psychological Trauma: Theory, Research, Practice, and Policy*, *12*(4), 331.  Klengel, T., & Binder, E.B. (2015). Epigenetics of Stress-Related Psychiatric Disorders and Gene × Environment Interactions. Neuron, 86(6), 1343-57.  Kok, B. E., & Singer, T. (2017). Effects of contemplative dyads on engagement and perceived social connectedness over 9 months of mental training: A randomized clinical trial. *JAMA Psychiatry,* *74*(2), 126−134.  Schiele, M.A., Gottschalk, M.G., & Domschke, K. (2020). The applied implications of epigenetics in anxiety, affective and stress-related disorders – A review and synthesis on psychosocial stress, psychotherapy and prevention. Clinical Psychology Review, 77, <https://doi.org/10.1016/j.cpr.2020.101830>  Singer, T., & Engert, V. (2019). It matters what you practice: Differential training effects on subjective experience, behavior, brain and body in the *ReSource Project*. *Current Opinion in Psychology, 28,* 151–158.  Singer, T., Kok, B. E., Bornemann, B., Zurborg, S., Bolz, M., & Bochow, C. (2016). *The ReSource Project: Background, design, samples, and measurements*. Max Planck Institute for Human Cognitive and Brain Sciences, Leipzig.Smith, B. W., Dalen, J., Wiggins, K., Tooley, E., Christopher, P., & Bernard, J. (2008). The brief resilience scale: assessing the ability to bounce back. *International journal of behavioral medicine*, *15*(3), 194-200.  Vinkers, C. H., Geuze, E., van Rooij, S. J., Kennis, M., Schür, R. R., Nispeling, D. M., ... & Boks, M. P. (2019). Successful treatment of post-traumatic stress disorder reverses DNA methylation marks. Molecular psychiatry, 1-8.  Wray, N.R., Lin, T., Austin, J., McGrath, J.J., Hickie, I.B., Murray, G.K., & Visscher, P.M. (2020). From Basic Science to Clinical Application of Polygenic Risk Scores: A Primer. JAMA Psychiatry, doi: 10.1001/jamapsychiatry.2020.3049. Epub ahead of print. PMID: 32997097.  Zannas, A.S., Arloth, J. Carrillo-Roa, T., Iurato, S., Röh, S., Ressler, K.J., Nemeroff, C.B., Smith, A.K., Bradley, B., Heim, C., Menke, A., Lange, J.F., Brückl, T., Ising, M., Wray, N.R., Erhardt, A., Binder, E.B., & Mehta, D. (2018). Lifetime stress accelerates epigenetic aging in an urban, African American cohort: relevance of glucocorticoid signaling. Genome Biology, 16, 266. doi: 10.1186/s13059-015-0828-5. Erratum in: Genome Biology, 19(1), 61. PMID: 26673150; PMCID: PMC4699359. |
| 3. Erläuterung der Bedeutung der Studie | Die Bedeutung der Studie begründet sich in der Notwendigkeit der wissenschaftlichen Untersuchung und Validierung von Online-Interventionen, die effektiv Stress reduzieren sowie das psychische Wohlbefinden und die soziale Kohäsion erhöhen. Die SARS-CoV-2 Pandemie stellt durch die massiven gesellschaftlichen Veränderungen und Maßnahmen wie „Physical Distancing“ einen großen Stressor dar, der nicht nur auf individueller Ebene Risikofaktoren für psychische Störungen wie sozialer Stress, wahrgenommene Einsamkeit oder erhöhte Ängstlichkeit steigen lässt, sondern auch auf gesellschaftlicher Ebene entscheidende Grundlagen des Zusammenhalts und der sozialen Kohäsion verändert.  Um diesen potentiell negativen Auswirkungen dieser SARS-CoV-2 Pandemie auf psychischer Ebene zu begegnen, soll mit dem online-app-basierten 10-wöchigen sozio-emotionalen Training (Affekt-Dyaden) und dem gleichlangen achtsamkeitsbasierten Training (Atemmeditation) geprüft werden, ob a) diese mentalen Programme zeitlich effizient und digital durchführbar sind und b) frühere positive Effekte auf Stressreduktion und Steigerung des mentalen sowie sozialen Wohlbefindens, die in sehr viel intensiveren mehrmonatigen nicht-online Trainingsprogrammen wie dem ReSource Projekt (Singer et al., 2016) beobachtet werden konnten (Engert & Singer, 2019), auch beobachtet werden können, wenn die mentalen Programme sehr verkürzt (auf nur 10 Wochen), nur online per App (ohne Einführungsretreats und ohne wöchentliche realen Sitzungen mit Lehrer*innen) sowie in sehr abgespeckter Form (Fokus auf eine 10-minütige Übung pro Tag) stattfindet. Die bisherige Forschung zu den affektiven Dyaden hat zwar gezeigt, dass diese Intervention das subjektive Empfinden sozialer Zusammengehörigkeit erhöht, aber die stressreduzierende sowie soziale Kompetenz und soziale Kohäsion fördernde Wirkung wurde bisher nur im Zusammenhang mit dem täglichen Üben anderer mentalen Übungen und dies über mehrere Monate aufgezeigt (Engert et al., 2017; Böckler et al., 2018). Diese Intervention soll nun im Kontext der erhöhten Belastung durch die Veränderungen infolge der SARS-CoV-2 Pandemie angewendet werden. Dies soll erlauben zu prüfen, ob solche mentalen Trainingsprogramme auch global als Online-Programme ohne große tägliche Anforderungen global skalierbar eingesetzt werden könnten, um den erhöhten Zahlen an Stress- und mentalen Erkrankungen in der Bevölkerung entgegenwirken zu können.  Die Erkenntnisse aus dieser Studie dienen sowohl der Entwicklung präventiver Interventionsmaßnahmen zur Reduktion von Einsamkeit und Stress in einem digitalen, zeitlich und örtlich flexiblen Format als auch der Untersuchung, inwiefern spezifische Risikogruppen besonders von der Intervention profitieren können.  Ein Verständnis über die grundlegenden biologischen Mechanismen, die mit einem erhöhten Krankheitsrisiko nach chronischer Stressbelastung einhergehen, könnte perspektivisch die Entwicklung von individuellen, mechanismen-basierten Therapieformen unterstützen, die auf eine Prävention oder Reversibilität mit biologischer Einbettung nach Stresserfahrungen abzielen.  *Literatur*  Engert, V., Kok, B. E., Papassotiriou, I., Chrousos, G. P., & Singer, T. (2017). Specific reduction in cortisol stress reactivity after social but not attention-based mental training. *Science Advances*, *3*(10), e1700495.  Böckler, A., Tusche, A., Schmidt, P., & Singer, T. (2018). Distinct mental trainings differentially affect altruistically motivated, norm motivated, and self-reported prosocial behaviour. *Scientific reports*, *8*(1), 1-14.  Singer, T., Kok, B. E., Bornemann, B., Zurborg, S., Bolz, M., & Bochow, C. (2016). *The ReSource Project: Background, design, samples, and measurements*. Max Planck Institute for Human Cognitive and Brain Sciences, Leipzig.Smith, B. W., Dalen, J., Wiggins, K., Tooley, E., Christopher, P., & Bernard, J. (2008). The brief resilience scale: assessing the ability to bounce back. *International journal of behavioral medicine*, *15*(3), 194-200. |
| 4. Welche der folgenden Bestimmungen finden  Anwendung  a) Medizinproduktegesetz  gemäß § 23b MPG - Ausnahme der klin.  Prüfung  b) Strahlenschutzgesetz und  Strahlenschutzverordnung  c) Gendiagnostikgesetz  d) Datenschutzgesetze:  - Konkrete Angabe des durch die  verantwortliche Stelle zu erfüllenden  Datenschutzgesetzes (für die Charité = -EU-Datenschutzgrundverordnung (DSGVO), Berliner Datenschutzgesetz - BlnDSG). - Ggf. entsprechend des Teilnehmerkreises zusätzlich zu beachtende Landesdatenschutzgesetze oder BDSG | Folgende Bestimmungen finden in der vorliegenden Studie Anwendung:   - Datenschutzgesetze: - EU-DSGVO - Berliner Datenschutzgesetz - Gendiagnostikgesetz |
| 5. Ggf.: Bezeichnung und Charakterisierung der Prüfprodukte | entfällt |
| 6. wesentliche Ergebnisse der vorklinischen Tests oder Gründe für die Nichtdurchführung derselben | entfällt |
| 7. Wesentlicher Inhalt und Ergebnisse der vorangegangenen Studien/Anwendungen der in der Studie zu prüfenden Produkte | entfällt |
| 8. Beschreibung der vorgesehenen Maßnahmen/Untersuchungsmethoden und eventuelle Abweichungen von den in der med. Praxis üblichen Maßnahmen/Untersuchungen (was ist „Routine“, was wird davon abweichend in der Studie gemacht?) Kommen validierte Fragebögen studienbedingt zum Einsatz, geben Sie bitte die Bezeichnung der Fragebögen an und wo diese publiziert sind (Referenzen). Nicht validierte Fragebögen bitte als Anlage beifügen. | Es sollen zwei mentale Trainingsprogramme (achtsamkeitsbasiert und sozio-emotionales Dyaden Training) miteinander und mit einer Re-test Kontrollgruppe verglichen werden.  Aus den *N* = 3522 Teilnehmer*innen, die vollständig an Phase 1 des CovSocial Projekts teilgenommen haben, sollen *n* = 300 Proband*innen (100 Proband*innen per Gruppe) für Phase 2 rekrutiert werden. Alle Teilnehmer*innen erhalten dazu eine E-Mail mit der Einladung zur Teilnahme an Phase 2 (siehe **Anlage A-1**). Diese E-Mail enthält neben generellen Informationen auch Ein- und Ausschlusskriterien sowie einen Link zum Online-Fragebogen für das Pre-Screening, welches vor Einschluss in die Studie durchgeführt wird. Dieses erfolgt über einen Online-Fragebogen (angesiedelt auf einem gesicherten Server der Max-Planck-Gesellschaft). Bei Interesse an einer Teilnahme und nach dem Ausfüllen einer Einverständniserklärung zur Teilnahme am Online-Screening können die Proband*innen aus Phase 1 diesen Fragebogen ausfüllen. Dazu haben sie nach Erhalt der Einladung per Mail 14 Tage Zeit. Die darin enthaltenen Fragen beziehen sich auf die Ein- und Ausschlusskriterien und sind in **Anlage B** genauer einzusehen. Proband*innen, die die Einschlusskriterien erfüllen (s.u.), werden per Email zu einem Online-Informationsabend eingeladen **(Anlage A-2)**, welcher einen Monat nach Erhalt der Einladungs-Email stattfindet. Proband*innen, die nach dem Informationsabend weiterhin Interesse an der Teilnahme an der Interventionsstudie haben können sich über einen Link in einer darauffolgenden Email **(Anlage A-3)** endgültig für die Interventionsstudie anmelden. Sie werden in der auf den Informationsabend folgenden Wochen 30-minütige Telefonate mit geschulten Achtsamkeitstrainer*innen **(Anlage C)** führen. Im Anschluss an die Telefonate findet die finale Zuordnung der Teilnehmer*innen zu den drei Gruppen statt. Die Zuordnung erfolgt nach stratifizierter Randomisierung auf Basis relevanter Variablen **(Anlage L)** und die Proband*innen geben ihr Einverständnis zur Teilnahme an der Hauptstudie der Phase 2 des CovSocial-Projekts. Die Hauptstudie besteht aus 2, optional 3 Untersuchungszeitpunkten Prätest, Posttest 1 und Posttest 2. Gruppe I und II führen die Intervention zwischen Prätest und Posttest 1 durch. Für Gruppe III erfolgt die Intervention nach Posttest 1.  **Abbildung 2.** *Ablauf der Testungen in Phase 2 des CovSocial-Projekts.*  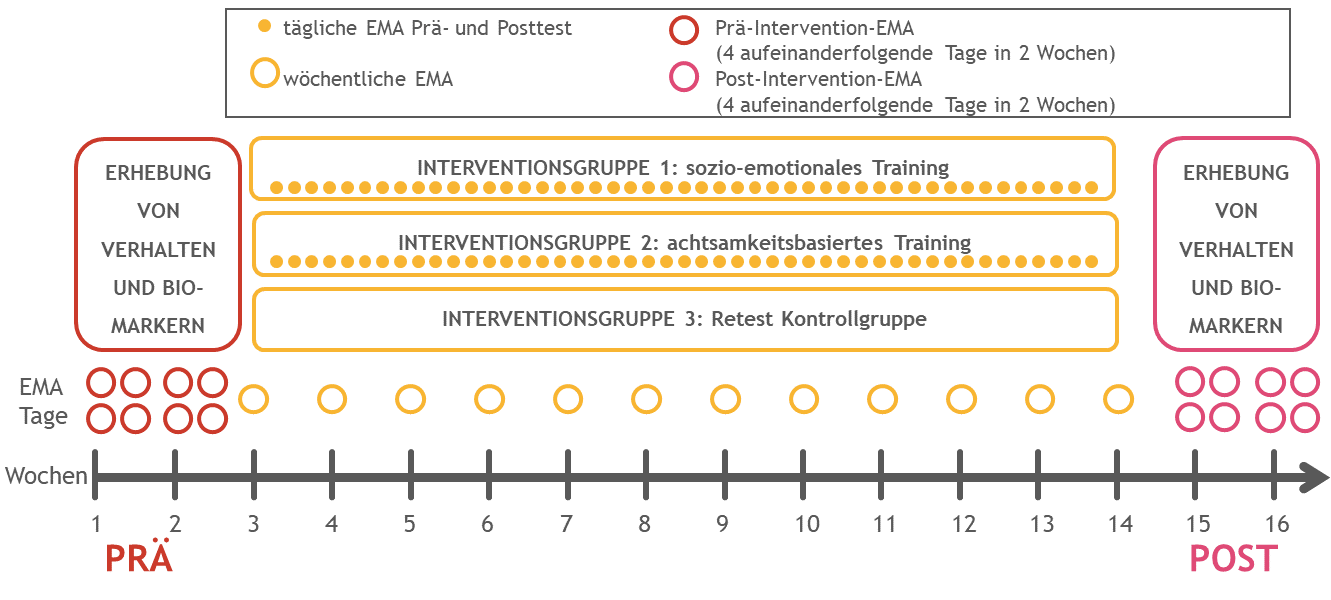  **Untersuchungsmethoden:**  **a) Prescreening**  Die in **Anlage B** aufgeführten Fragen werden von allen interessierten Teilnehmer*innen aus Phase 1 des CovSocial-Projekts ausgefüllt.  **b) Prä- und Post-Testung**  *Ecological Momentary Assessment, „EMA“:* Über die CovSocial-App erhalten die Teilnehmer*innen in zwei aufeinander folgenden Wochen an je vier aufeinander folgenden Tagen Fragen zu ihrem aktuellen Befinden (siehe **Anlage D**). Diese vier Tage verteilen sich auf zwei Wochentage und 2 Wochenendtage, um ein möglichst breites Spektrum im Erleben und Empfinden der Teilnehmer*innen im Alltag abzudecken und beziehen sich auf folgende Lebensbereiche: Schlaf (nur zum jeweils ersten Zeitpunkt des Tages), Stress, Affektive Stimmung, Valenz sowie zeitlicher und sozialer Bezug der eigenen Gedanken, Einsamkeit.  Die Beantwortung der Fragebögen dauert jeweils circa 2 Minuten. Zudem sollen die Teilnehmer*innen pro Tag fünf Speichelproben sammeln (unmittelbar nach dem Erwachen, +30, +60, +240 und +300 Minuten nach dem Erwachen). Die Materialien hierzu werden ihnen bei der Teilnahme an der vor Ort Untersuchung der behavioralen Daten mitgegeben oder, falls dieser Termin nach der geplanten EMA—Untersuchung liegt, postalisch zugeschickt. Die Teilnehmer*innen bringen diese gesammelten Materialen nach Ende der Prä- bzw. Post-Testung ins Forschungslabor der Forschungsgruppe Soziale Neurowissenschaften zurück oder schicken diese ebenfalls postalisch.  *Blutprobe:* Zum Prätest wird mittels peripherer Venenpunktion 50 ml Blut gewonnen um folgende Marker zu bestimmen:   - endokrine Marker: z.B. Cortisol, DHEAS, Adrenalin, Noradrenalin - immunologische Marker: pro- und anti-inflammatorische Zytokine, CRP - metabolische Marker: z.B. Adipokine, Leptin, HbA1c, Triglyceride, gesättigte und ungesättigte Fettsäuren). - Marker der Zellalterung: z.B. Telomerlänge, mitogen-stimulierte Telomeraseaktivität, P16INK4a, DNA re-pair capacity, p53)   Daraus wird ein „allostatic load score“ gebildet, der die physiologischen Konsequenzen einer chronischen Stress-Exposition über verschiedene Systeme hinweg abbildet (Juster et al., 2010).  *Eine Erhebung genomischer Marker aus Speichel DNA:* Zum Prätest und Posttest I werden mittels Speichelproben folgende epigenetische Marker gewonnen:   - DNA Methylierung, genomweit, gemessen z.B. mit kommerziellen Methylierungs-Arrays, wie dem Illumina EPIC DNA Methylation Array und gezielten Untersuchungen mittels „targeted bisulfite sequencing“   *Fragebögen*: Folgende Fragebögen werden online über die WebApp des CovSocial-Projekts zum Prätest, Posttest 1 und Posttest 2 ausgefüllt. Mithilfe standardisierter Fragebögen werden psychische und verhaltensbezogene Faktoren erfasst:   - Perceived Stress Scale (PSS-10; Cohen et al., 1983; Cohen & Williamson; 1988) - UCLA Loneliness Scale (Döring & Bortz, 1993; Russell, Peplau & Cutrona, 1980) - Beck Depression Inventory-II (BDI-II; Beck, Steer & Brown, 1996) - Connor-Davidson Resilience Scale (CD-RISC; Connor & Davidson, 2003) - State-Trait Anxiety Inventory (STAI; Spielberger, 2010) - Cognitive Emotion Regulation Questionnaire (CERQ; Garnefski, Kraaij, Spinhoven, 2001; Loch, Hiller & Witthöft, 2011) - Difficulties in Emotion Regulation Scale (DERS; Kaufman et al., 2016) - Brief Resilience Scale (BRS; Chmitorz et al., 2018; Smith et al., 2008) - Prosocialness Scale for Adults (PSA; Caprara et al., 2005) - Self-Compassion Scale (SCS; Hupfeld & Ruffieux, 2011; Raes et al., 2011) - Fear of Compassion Scale (FoC; Gilbert et al., 2012) - Saarbrücker Persönlichkeitsfragebogen/Interpersonal Reactivity Index (IRI; Davis, 1980; Paulus, 2009) - Social Value Orientation (Murphy, Ackermann & Handgraaf, 2011)   *Behaviorale Daten:* Die behavioralen Daten werden mithilfe von computergestützen Experimenten im Forschungslabor erhoben. Diese erheben Daten zu folgenden Faktoren:   - EmpaToM (Böckler et al, 2014) - ReSource Project spieltheoretische Paradigmen zu altruistischem prosozialem Verhalten (e.g. Böckler et al., 2018; Böckler, Tusche & Singer, 2016; Leiberg, Klimecki & Singer, 2011; Singer et al., 2016) - Dot probe task (Macleod, Matthews & Tata, 1986) - Scrambled Sentences Task (Everaert et al., 2014) - Affective Flexibility Task (Malooly, Genet & Siemer, 2013) - Implicit Association Test (Greenwald et al., 2003)   Daten zur frühkindlichen Stressbelastung, zur subjektiven Stressempfinden und zu psychischer und körperlicher Gesundheit während der Pandemie stehen aus CovSocial Phase 1 zur Verfügung.  **c) Intervention:** Die Interventionsphase besteht aus jeweils 10 Wochen. Dabei werden sechsmal pro Woche online mentale Übungen (sozio-emotionale Übung, die Affektdyade, für Gruppe I und achtsamkeitsbasierte Übungen wie Atemmeditation für Gruppe II) durchgeführt. Beide Übungen werden über die mobile CovSocial App durchgeführt. Die Affektdyade besteht aus einer 12-minütigen Übung zu zweit. Die Atemmeditation besteht aus einer 10-minütigen Übung, die alleine durchgeführt wird. Vor und nach den Übungen findet per App eine kurze Befragung statt (**Anlage E**). Ein genaues Protokoll beider Interventionen ist **Anlage F** zu entnehmen. Zudem finden einmal pro Woche 2-stündige Online-Treffen statt, in denen die Intervention mit geschulten Meditationslehrer*innen geübt wird, Feedback gegeben wird und Fragen gestellt werden können. Während der Interventionsphase wird zudem einmal pro Woche über die App eine Fragebogen-Erhebung durchgeführt. Die Teilnehmer*innen erhalten an diesem Tag Fragen zu ihrem aktuellen Befinden, insbesondere bezüglich Stress, Emotionsregulation, Sorgen, psychologische Flexibilität, Interozeption und Mitgefühl, die sie über die CovSocial-App am Handy oder am Computer beantworten können. Folgende Fragebögen werden hierfür verwendet:   - Mindfulness (CAMS-R) - CERQ (subscales Acceptance, Rumination, Positive Reappraisal) - Perceived Stress Scale – 4 (PSS-4) - Fear of Compassion Scale (FoC; Gilbert et al., 2012) subscale fear of expressing compassion for others - Self-Compassion Scale (SCS; Hupfeld & Ruffieux, 2011; Raes et al., 2011) subscale self-kindness - IRI (Davis, 1980; Paulus, 2009) subscales Personal Distress and Empathic Concern - Cube of Thoughts (Kok & Singer, 2017) - Worry (Penn State Worry Questionnaire, Kertz et al., 2014) - Cognitive Control and Flexibility Questionnaire (Gabrys et al., 2018) - Acceptance and Action Questionnaire (AAQ-II; Bond et al., 2011) - Coping strategies (Brief COPE; Carver, 1997) - 4 stress assessment questions (Smyth et al., 2017) - Fragen zu sozialer Zugehörigkeit wie in Phase 1 des CovSocial Projekts:   - **Ziehen Sie die Kreise so, dass Sie Ihre Zugehörigkeit zu den folgenden Gruppen am besten darstellen.**   *„Hinweis: Je näher Sie die Kreise zueinander ziehen, desto zugehöriger fühlten Sie sich dieser Gruppe im Januar 2020.“*   - - Ich und Familie   - Ich und Freund*innen   - Ich und Nachbarschaft   - Ich und Berliner Bevölkerung   - Ich und Deutsche Bevölkerung   - Ich und Europäische Bevölkerung   - Ich und Weltbevölkerung   **Posttestung:** Die Posttestung findet identisch zur Prä-Messung statt.  *Literatur:*  Beck, A. T., Steer, R. A., & Brown, G. (1996). Beck depression inventory–II. *Psychological Assessment*.  Berg, J., Dickhaut, J. & McCabe, K. (1995). Trust, Reciprocity, and Social History. *Games and Economic Behavior, 10*, 122-142.  Böckler, A., Tusche, A., Schmidt, P., & Singer, T. (2018). Distinct mental trainings differentially affect altruistically motivated, norm motivated, and self-reported prosocial behaviour. *Scientific Reports, 8:*13560*.*  Böckler, A., Kanske, P., Trautwein, F.-M., & Singer, T. (2014). *The EmpaToM: A novel fMRI-task separating affective and cognitive routes to social cognition.* Poster presented at 20th Annual Meeting of the Organization for Human Brain Mapping (OHBM), Hamburg, Germany.  Bond, F. W., Hayes, S. C., Baer, R. A., Carpenter, K. M., Guenole, N., Orcutt, H. K., ... & Zettle, R. D. (2011). Preliminary psychometric properties of the Acceptance and Action Questionnaire–II: A revised measure of psychological inflexibility and experiential avoidance. *Behavior Therapy, 42(4),* 676-688.  Caprara, G. V., Steca, P., Zelli, A., & Capanna, C. (2005). A new scale for measuring adults' prosocialness. *European Journal of psychological assessment*, *21*(2), 77-89.  Carver, C. S. (1997). You want to measure coping but your protocol’too long: Consider the brief cope. *International Journal of Behavioral Medicine, 4(1),* 92-100.  Chmitorz, A., Wenzel, M., Stieglitz, R. D., Kunzler, A., Bagusat, C., Helmreich, I., ... & Lieb, K. (2018). Population-based validation of a German version of the Brief Resilience Scale. *PloS one*, *13*(2), e0192761.  Cohen S, Kamarck T, Mermelstein R, 1983. A global measure of perceived stress. J Health Soc Behav 24, 385–396.  Cohen S, Williamson G, 1988. Perceived stress in a probability sample of the United States., in: Spacapan S, Oskamp S (Eds.), The Social Psychology of Health. Sage, Newbury Park, CA.  Connor, K. M., & Davidson, J. R. (2003). Development of a new resilience scale: The Connor‐Davidson resilience scale (CD‐RISC). *Depression and Anxiety, 18(2),* 76-82.  Davis, M. H. (1980). A multidimensional approach to individual differences in empathy. *JSAS Catalog of Selected Documents in Psychology, 10,* 85*.*  Döring, N., & Bortz, J. (1993). Psychometrische Einsamkeitsforschung: Deutsche Neukonstruktion der UCLA Loneliness Scale. *Diagnostica, 39(3)*, 224-239.  Engert, V., Kok, B. E., Papassotiriou, I., Chrousos, G. P., & Singer, T. (2017). Specific reduction in cortisol stress reactivity after social but not attention-based mental training. *Science Advances,* *3*(10): e1700495.  Everaert, J., Duyck, W., & Koster, E. H. (2014). Attention, interpretation, and memory biases in subclinical depression: A proof-of-principle test of the combined cognitive biases hypothesis. *Emotion, 14(2),* 331.  Gabrys, R. L., Tabri, N., Anisman, H., & Matheson, K. (2018). Cognitive control and flexibility in the context of stress and depressive symptoms: The cognitive control and flexibility questionnaire. *Frontiers in Psychology, 9*, 2219.  Garnefski, N., Kraaij, V., & Spinhoven, P. (2001). Negative life events, cognitive emotion regulation and emotional problems. *Personality and Individual differences*, *30*(8), 1311-1327.  Gilbert, P., McEwan, K., Gibbons, L., Chotai, S., Duarte, J., & Matos, M. (2012). Fears of compassion and happiness in relation to alexithymia, mindfulness and self-criticism. *Psychology and Psychotherapy*, *85*, 374–390. DOI:10.1111/j.2044-8341.2011.02046.x  Greenwald, A. G., Nosek, B. A., & Banaji, M. R. (2003). Understanding and using the implicit association test: I. An improved scoring algorithm. *Journal of personality and social psychology*, *85*(2), 197.  Hupfeld, J. & Ruffieux, N. (2011). Validierung einer deutschen Version der Self-Compassion Scale (SCS-D). *Zeitschrift für Klinische Psychologie und Psychotherapie, 40 (2),* 115–123.  Juster, R. P., McEwen, B. S., & Lupien, S. J. (2010). Allostatic load biomarkers of chronic stress and impact on health and cognition. *Neuroscience & Biobehavioral Reviews*, *35*(1), 2-16.  Kaufman, E.A., Xia, M., Fosco, G., Yaptangco, M., Skidmore, C.R., & Crowell, S. (2016). The Difficulties in Emotion Regulation Scale Short Form (DERS-SF): validation and replication in adolescent and adult samples. *Journal of Psychopathology and Behavioral Assessment, 38(443*), 443-455.  Kertz, S. J., Lee, J., & Björgvinsson, T. (2014). Psychometric properties of abbreviated and ultra-brief versions of the Penn State Worry Questionnaire. *Psychological assessment, 26(4),* 1146.  Kok, B. E., & Singer, T. (2017). Effects of contemplative dyads on engagement and perceived social connectedness over 9 months of mental training: A randomized clinical trial. *JAMA Psychiatry,* *74*(2), 126−134.  Leiberg, S., Klimecki, O., & Singer, T. (2011). Short-Term Compassion Training Increases Prosocial Behavior in a Newly Developed Prosocial Game. *PLoS ONE 6(3)*: e17798. https://doi.org/10.1371/journal.pone.0017798  Loch, N., Hiller, W., & Witthöft, M. (2011). Der cognitive emotion regulation questionnaire (CERQ). *Zeitschrift für Klinische Psychologie und Psychotherapie*.  Macleod, C., Mathews, A., & Tata, P. (1986). Attentional bias in emotional disorders. *Journal of Abnormal Psychology, 95(1),* 15–20.  Malooly, A. M., Genet, J. J., & Siemer, M. (2013). Individual differences in reappraisal effectiveness: the role of affective flexibility. *Emotion, 13(2),* 302.  Murphy, R. O., Ackermann, K. A., & Handgraaf, M. (2011). Measuring social value orientation. *Judgment and Decision making, 6(8),* 771-781.  Paulus, C. (2009). *Der Saarbrücker Persönlichkeitsfragebogen SPF (IRI) zur Messung von Empathie: psychometrische Evaluation der deutschen Version des Interpersonal Reactivity Index*. URL: http://psydok. sulb. uni-saarland. de/volltexte/2009/2363.  Raes, F., Pommier, E., Neff,K. D., & Van Gucht, D. (2011). Construction and factorial validation of a short form of the Self-Compassion Scale. *Clinical Psychology & Psychotherapy, 18*, 250-255.  Russell, D., Peplau, L. A., & Cutrona, C. E. (1980). The revised UCLA Loneliness Scale: concurrent and discriminant validity evidence. *Journal of personality and social psychology*, *39*(3), 472.  Singer, T., & Engert, V. (2019). It matters what you practice: Differential training effects on subjective experience, behavior, brain and body in the *ReSource Project*. *Current Opinion in Psychology, 28,* 151–158.  Singer, T., Kok, B. E., Bornemann, B., Zurborg, S., Bolz, M., & Bochow, C. (2016). *The ReSource Project: Background, design, samples, and measurements*. Max Planck Institute for Human Cognitive and Brain Sciences, Leipzig.Smith, B. W., Dalen, J., Wiggins, K., Tooley, E., Christopher, P., & Bernard, J. (2008). The brief resilience scale: assessing the ability to bounce back. *International journal of behavioral medicine*, *15*(3), 194-200.  Smyth, J. M., Zawadzki, M. J., Juth, V., & Sciamanna, C. N. (2017). Global life satisfaction predicts ambulatory affect, stress, and cortisol in daily life in working adults. *Journal of Behavioral Medicine, 40(2*), 320-331.  Spielberger, C. D. (2010). State‐Trait anxiety inventory. *The Corsini encyclopedia of psychology*, 1-1. |
| 9. Bewertung und Abwägung der vorhersehbaren Risiken und Nachteile der Studienteilnahme gegenüber dem erwarteten Nutzen für die Studienteilnehmer und zukünftig erkrankte Personen (Nutzen-Risiko-Abwägung) | Wir erwarten keinerlei potentielle Nachteile der Studienteilnehmer*innen. Die potentiellen Risiken sind sehr gering (s.u.). Die invasive Untersuchungsmethode der Blutentnahme wird von erfahrenem medizinischem Personal durchgeführt. Die Intervention wird von geschultem Fachpersonal (d.h. Trainer*innen achtsamkeitsbasierter Verfahren) durchgeführt, um das Risiko für die Teilnehmer*innen so gering wie möglich zu halten.  Der Nutzen für die Studienteilnehmer*innen ist durch die geplante Intervention zur Reduktion von Einsamkeit und Stress sowie zur Steigerung des psychischen Wohlbefindens potenziell sehr hoch. |
| a. zu prüfender medizinischer Nutzen für die Studienteilnehmer (individueller Nutzen für den einzelnen Patienten) | Der potentielle Nutzen der Studienergebnisse für die Studienteilnehmer*innen ist hoch, da die Teilnahme an mentalen Trainingsprogrammen zur Stress- und Einsamkeitsreduktion sowie zur Erhöhung der sozialen Kohäsion das psychische Wohlbefinden sowie Resilienz verbessern sowie präventiv die Entwicklung psychischer Störungen infolge einer hohen Stressbelastung entgegenwirken kann. |
| b. zu prüfender medizinischer Nutzen für zukünftig erkrankte Personen (Gruppennutzen) | Der potentielle Nutzen der Studienergebnisse für die Zukunft für Personen mit erhöhter Stressbelastung, sozialer Isolation sowie mentaler Risikofaktoren ist hoch, da aufgrund der Erkenntnisse dieser Studie frühzeitige Präventions- und Interventionsmaßnahmen entwickelt werden können, die der Entwicklung psychischer Störung infolge einer hohen Stressbelastung sowie sozialer Isolation entgegenwirken können. |
| c. **Risiken** und Belastungen für die  Studienteilnehmer (alle im Einzelnen auflisten) | Blutentnahme: Die Blutentnahme erfolgt über eine Venenpunktion, dabei gibt es seltene Risiken einer Infektion oder Verletzung von benachbartem Gewebe. Außer einem kurzen Schmerz beim Einstich der Nadel kann es gelegentlich zu einer leichten Einblutung kommen, die innerhalb weniger Tage verschwindet. Einige Personen reagieren auf eine Blutabnahme mit einer Kreislaufreaktion.  Fragebögen/ Interviews: Einige der Fragebögen und Interviews behandeln Informationen, die persönlicher und privater Natur sind und die, sofern sie außerhalb des Forschungskontexts gelangen würden, ein mögliches soziales Risiko darstellen könnten. Auch können durch einige der Fragebögen negative Erinnerungen geweckt und eventuelle Gefühle der Scham, Hilflosigkeit, Wut etc. ausgelöst werden.  Speichelproben: Es sind keine Risiken mit der Entnahme von Speichelproben verbunden.  Ganz generell bringt die Teilnahme an einer genetischen Untersuchung bzw. die Erhebung von Gesundheitsdaten das Risiko der Verletzung der Privatsphäre mit sich, mit eventuell negativen sozialen, psychologischen und ökonomischen Folgen. Aus den folgenden Gründen ist das Risiko in dieser Studie jedoch minimal: 1) Die Untersuchung des genetischen Materials dient **nicht** der Diagnostik genetischer Erkrankungen. 2) Alle Proben und genetischen Informationen werden außerdem zu jeder Zeit separat von Ihren persönlichen Informationen aufbewahrt und analysiert und nicht an Dritte weitergegeben. Es erfolgt daher auch keine persönliche Rückmeldung zu den Ergebnissen der genetischen Untersuchung. 3) Die Personen, welche die genetischen Merkmale messen, haben keinen Zugang zu Ihren persönlichen Informationen und haben keinen Zugriff auf die Kodierliste (siehe unten). Eine Verbindung zu den Proband*innen kann somit nicht hergestellt werden. 4) DNA Proben werden 10 Jahre nach Abschluss der Studie vernichtet und bis dahin einem zugangsgeschützten Biobank-Repositorium (MPI Psychiatrie) in pseudonymisierter Form gelagert. **Auf Grundlage der Daten ist nach Abschluss der Datenerhebung das Risiko eines direkten Rückschlusses auf eine Person zwar nicht ausgeschlossen, aber dennoch bestmöglich minimiert.**  **Intervention:**  Bei dem sozio-emotionalen Training mit täglichen Affekt-Dyaden kann das laute Erzählen von schwierig erlebten Situationen dazu führen, dass durch das Erzählen von negativen Emotionen sowohl beim Zuhörer als auch beim Sprecher negative Gefühle entstehen, die auch nach der mentalen Übung noch nachwirken können. Auch können achtsamkeitsbasierte Meditationen für Anfänger als unangenehm oder schwierig empfunden werden.  Um jedoch mögliche negative Erfahrungen abzufangen und zu besprechen, sind die wöchentlichen Treffen mit den Lehrer*innen eingeplant. Zudem wird wie unten im Detail beschrieben ein zwei-schrittiges Screeningverfahren getätigt, um Proband*innen mit psychischen Vorerkrankungen sowie mentaler Instabilität vor der Studie auszuschließen. |
| 10. Maßnahmen zur Risikobeherrschung | Die im Prescreening (**Anlage** B) erhobenen Daten sowie die zuvor geführten Telefonate sollen zudem das Risiko minimieren, dass Personen mit psychischen Erkrankungen oder mentaler Instabilität an der Interventionsstudie teilnehmen.  Da es grundsätzlich bei der web-basierten Erfassung und Speicherung von Daten ein Risiko von Datenpannen und Datenmissbrauch besteht, werden technische und organisatorische Maßnahmen zum Datenschutz und der Datensicherheit im Sinne der EU-DSGVO angewendet. |
| 11. Abbruchkriterien | Die Teilnahme an dieser Studie erfolgt auf freiwilliger Basis. Das vorzeitige Ausscheiden einer/s Proband*in aus der Studie erfolgt auf persönlichen Wunsch de/rs Proband*in, bei Kontaktverlust oder nach jedem Ereignis, welches nach Ansicht der Studienleitung eine weitere Studienteilnahme ausschließt. |
| 12. Anzahl, Alter und Geschlecht der betroffenen Personen | Die Rekrutierung der Stichprobe erfolgt innerhalb der Gruppe der Proband*innen, die an Phase 1 des CovSocial-Projekts teilgenommen haben (erwachsene Berliner*innen, 18-65 Jahre). Aus dieser Stichprobe von N = 3522 Proband*innen erfolgt die Einladung per E-Mail für die Teilnahme am Prescreening-Fragebogen (**Anlage** B). Ziel ist es, 300 Versuchspersonen den drei Gruppen (Sozio-emotionales und achtsamkeitsbasiertes Training, Retest-Kontrolle) randomisiert zuzuordnen. |
| 13. Biometrische Planung mit Angabe der statistischen Methodik, einschließlich der Begründung der Fallzahl. Angabe des/der Statistikers/Statistikerin  (sofern Beratung durch das Institut für Biometrie der Charité erfolgt, muss eine Unterschrift eingefügt werden) | Zur Überprüfung der Interventionseffekte werden gemischte Modelle verwendet, in denen Messzeitpunkt (Prätest und Posttest 1) und Gruppe (Gruppe I: Sozio-emotionale Intervention, Gruppe II: Achtsamkeitsintervention, Gruppe III: Re-test Kontrollgruppe + sozio-emotionale Intervention) sowie deren Interaktion als fixe Effekte festgelegt werden, sowie zufällige Effekte (within-subjects) über die Teilnehmer*innen-ID definiert werden. A-priori-Teststärkeanalysen wurden auf der Grundlage von vorherigen Ergebnissen aus einer mentalen Trainingsstudie, dem ReSource Projekt (Singer et al., 2016), erstellt, das die geplanten mentalen Trainings-Interventionen als Elemente enthielt. Die Effektstärken der Effekte nach den umfangreicheren 3-monatigen Trainingsmodulen wurden in den jeweils getesteten Kohorten von n=80 dabei als niedrig bis mittel eingestuft (z.B. Hildebrandt et al., 2017; Singer & Engert, 2019). Es ist möglich, dass die Interventionseffekte der sozio-emotionalen und achtsamkeitsbasierten online-Interventionen ohne den Kontext anderer Interventionselemente niedriger ausfallen können. Für die Berechnung von A-priori-Teststärkeanalysen wurde das Computerprogramm G*Power (Faul et al., 2007) verwendet. Hierbei wurde auf Varianzanalysen mit Messwiederholung und Interaktionen von Zwischengruppen- und Innergruppenvariablen Bezug genommen. Basierend auf einem alpha-Niveau von .05 und mit einer Power (1-ß) von .80, 3 Gruppen und 2 Messzeitpunkten, einer Korrelation von Variablen mit Messwiederholung von .39, die sich als untere Grenze der Retest-Reliabilität für die Cortisol Awakening Response (CAR) bestimmten ließ (Pruessner et al., 1997), und einer angenommenen kleinen Effektstärke von f = .10 ergibt sich eine Stichprobengröße von n=297. Mit einer geplanten Fallzahl von insgesamt n=300 lassen sich bei den verwendeten psychologischen Fragebögen, die durch höhere Retest-Reliabilitäten von mindestens .70 gekennzeichnet sind, geringe Effektstärken von bis zu f = .07 erfassen. Bei der Hinzunahme des dritten Messzeitpunkts (Posttest 2) kann unter gleichen Annahmen (Fallzahl n=300, Power (1-ß) = .80, α= .05) für Messmethoden mit einer Retest-Reliabilität von .39 einen Effekt der Stärke f = .09 und für Messmethoden mit einer Retest-Reliabilität von .70 einen Effekt der Stärke f = .06 erfasst werden.  Für genetische Marker betreffen unsere Hypothesen (H9) Zusammenhänge zwischen genetischen Daten aus Phase 1 des CovSocial Projekts und den unter Hypothesen 1-6 formulierten Interventionseffekten. Da diese genetischen Daten bereits erhoben wurden, ist für diese Fragestellungen keine biometrische Planung erforderlich.  Angesichts des relativen Mangels an Studien, die den Einfluss mentaler Trainings-Interventionen auf Veränderungen der DNA-Methylierung über die Zeit untersuchen, ist es schwierig, die erwarteten Effektgrößen abzuschätzen. Hypothesen (H9-H10) zur prä-post-interventionellen Veränderung der epigenetischen Marker werden daher explorativ analysiert.  *Literatur*  Faul, F., Erdfelder, E., Lang, A.-G., & Buchner, A. (2007). G*Power 3: A flexible statistical power analysis program for the social, behavioral, and biomedical sciences. *Behavior Research Methods, 39,* 175-191.  Hildebrandt, L. K., McCall, C., & Singer, T. (2017). Differential effects of attention-, compassion-, and socio-cognitively based mental practices on self-reports of mindfulness and compassion. *Mindfulness, 8(6),* 1488-1512.  Pruessner, J.C., Wolf, O.T., Hellhammer, D.H., BuskeKirschbaum, A., von Auer, K., Jobst, S., Kaspers, F., Kirschbaum, C., 1997. Free cortisol levels after awakening: a reliable biological marker for the assessment of adrenocortical activity. Life Science, 61, 2539–2549.  Singer, T., & Engert, V. (2019). It matters what you practice: Differential training effects on subjective experience, behavior, brain and body in the ReSource Project. Current Opinion in Psychology, 28, 151–158.  Singer, T., Kok, B. E., Bornemann, B., Zurborg, S., Bolz, M., & Bochow, C. (2016). *The ReSource Project: Background, design, samples, and measurements*. Max Planck Institute for Human Cognitive and Brain Sciences, Leipzig. |
| 14.  a. Darlegung und ggf. Erläuterung der **Ein- und Ausschlusskriterien** | Einschlusskriterien   - Teilnahme an Phase 1 - Einwilligungsfähigkeit - schriftliches Einverständnis - Alter 18-65 Jahre - Berliner Allgemeinbevölkerung - Deutsches Sprachverständnis zur Teilnahme an der sprachbasierten Intervention und selbstständigen Bearbeitung der Fragebögen notwendig   Ausschlusskriterien   - Fehlende technische Ausrüstung: kein Zugang zu Internet oder kein Mobiltelefon zur Nutzung einer App - Psychologie-Studierende - Erfahrung mit spiritueller Praxis - Regelmäßige Yoga-Praxis mit meditativen Anteilen innerhalb der letzten zwei Jahre - Medikation mit Auswirkung auf die erhobenen physiologischen Marker - Akute psychotherapeutische Behandlung oder Diagnose einer psychischen Störung innerhalb der letzten zwei Jahre - Diagnose einer psychotischen Störung - Ernsthafte Erkrankung oder chronische Schmerzen - Neurologische Einschränkungen - Alexithymie (TAS-20 > 60) - Erhöhte Werte auf Skalen zur Depressiven (PHQ-9 > 19) und Angstsymptomen (GAD-7 > 15) - Teilnahme an anderen Stressreduktions-Programmen - Suizidalität (gemessen mit PHQ-9 Item) - Telefonische Lehrer*innenbeurteilung mentaler Gesundheit oder Gegenindikation nach Einzelscreening (**Anlage C**) - Aktuelle Teilnahme an anderen Programmen teilnehmen, deren Ziele mit denen der hier vorgelegten Studie vergleichbar sind oder Vorhaben einer solche Teilnahme |
| b. **Studieninformation** (wer diese mündlich und schriftlich erteilt und Angabe, wie viel Zeit zwischen Aufklärung und Einwilligung verbleibt  (schriftliche Information als Anlage) | Die Teilnahmeinformation dieser Studie erfolgt schriftlich zu Beginn als Teil der online-basierten Datenerhebung. Siehe auch: **Anlage G „Teilnahmeinformation Prescreening“ und Anlage H „Teilnahmeinformation Studie“**  Hierbei werden zwei separate Teilnahmeinformationen erfolgen: Eine für das Prescreening und eine weitere für die finale Stichprobe der Teilnehmer*innen zu Beginn der Interventionsphase. |
| c. **Einwilligungserklärung** (schriftliche Form als Anlage | **Anlage I „Einwilligungserklärung Prescreening“ und Anlage J „Einwilligungserklärung Studie“**  Hierbei werden zwei separate Einwilligungserklärungen erfolgen: Eine für das Prescreening und eine weitere schriftliche für die finale Stichprobe der Teilnehmer*innen zu Beginn der Interventionsphase.  Die Einwilligungserklärungen erfolgen online. |
| d. Ggf. **Information und Einwilligung des gesetzlichen Vertreters** (ggf. auch  Beschreibung des Verfahrens zur Einrichtung einer gerichtlichen Betreuung) | entfällt |
| 15. Maßnahmen zur Gewinnung von Studienteilnehmern (Aushang?, Zeitungsannoncen? Etc.) | Es werden alle diejenigen Proband*innen per Email kontaktiert, die Ihre Einwilligung zur Teilnahme an weiteren Phasen der Studie zu Beginn der Phase 1 der CovSocial-Studie („Online-Erhebung von sozialem Zusammenhalt und psychischer Gesundheit während der SARS-CoV-2-Pandemie“) gegeben haben. |
| 16. Ggf.: **Grund für die Einbeziehung und Darlegung des therapeutischen Nutzens für Personen, die minderjährig und/oder nicht einwilligungsfähig sind.** | entfällt |
| 17. Beziehung zwischen Studienteilnehmer und Studienarzt/-ärztin (Ist der Studienarzt zugleich der  behandelnde Arzt?) | entfällt  Es handelt sich um keine klinische Proband*innen-Population. |
| 18. Erklärung zur Einbeziehung möglicherweise vom Sponsor abhängiger Personen | Vom Sponsor abhängige Personen werden nicht in die Studie eingeschlossen. |
| 19. Maßnahmen, die eine Feststellung zulassen, ob ein Studienteilnehmer an mehreren Studien zugleich oder vor Ablauf einer in der vorangegangenen Studie festgelegten Frist teilnimmt.  Ist die Teilnahme an mehreren Studien möglich? | Die Proband*innen werden im Rahmen des Prescreenings (**Anlage B**) gefragt, ob sie aktuell an anderen Programmen teilnehmen, deren Ziele mit denen der hier vorgelegten Studie vergleichbar sind oder eine solche Teilnahme planen. |
| 20. Ggf.: Honorierung bzw. Kostenerstattung der Studienteilnehmer (Höhe, wofür soll gezahlt werden?) | Für die Teilnahme an der Studie gibt es eine Aufwandsentschädigung entsprechend des zeitlichen Aufwands der Proband*innen. Es wird die Zeit für die Fragebogen, die Erhebungen im Labor (Verhaltenstests und Blutproben), Speichelproben Zuhause und eine pauschale Fahrtzeit von 30 Minuten/Fahrt entschädigt.  Dieser wird voraussichtlich für die Prätestung 8,5 Stunden betragen. Für die Interventionsphase wird der Zeitaufwand für die täglichen/wöchentlichen Fragen über die Webapp entschädigt. Dieser beträgt ca. 20 min pro Woche, dementsprechend 3,5 Stunden für den 10-wöchigen Interventionszeitraum. Die Posttestung wird voraussichtlich ca. 7 Stunden betragen. Die Höhe der Entschädigung beträgt 10€ pro Stunde  In Anbetracht der aufgewendeten Zeit, die für die Studienteilnahme seitens der Proband*innen aufgebracht werden muss, stellt dieser Betrag (10€/Stunde) eine angemessene Kompensation dar, motiviert aber gleichzeitig keine Teilnahme aus finanziellen Gründen. Die Aufwandsentschädigung wird auf das Bankkonto der jeweiligen Proband*innen überwiesen. Die Proband*innen füllen dafür einen Antrag auf Erstattung aus (**Anlage K).** |
| 21. Ggf.: Plan für die Weiterbehandlung und medizinische Betreuung der betroffenen Personen nach dem Ende der Studie | entfällt |
| 22. Ggf.: Versicherung der Studienteilnehmer  (Versicherungsbestätigung und Versicherungsbedingungen, Versicherer, Versicherungsumfang, Versicherungsdauer) | entfällt |
| 23. Dokumentationsverfahren:  - Ggf. Verweis auf CRF-Bögen  -Angabe der zu erfassenden Daten  - Probenumgang  - Aufbewahrung / Archivierung (inkl. Fristen)  - Zugang zu den Daten und Proben | Die erhobenen personenbezogenen und pseudonymisierten Daten werden in elektronischer Form dokumentiert.  Die Studiendaten umfassen:   - Fragebögen/ Interviewdaten in digitaler Form - Biologischen Proben - Behaviorale Daten aus computerbasierten Experimenten sowie in diesem Zusammenhang stehende Fragen und Instruktionen auf Papierbögen.   Die Daten werden in verschlüsselter Form verarbeitet und gespeichert. Hierzu versieht  die Studienleitung die Daten mit einer Codenummer (Pseudonymisierung der Daten). Auf den Codeschlüssel, der es erlaubt, die studienbezogenen Daten mit den personenbezogenen Daten in Verbindung zu bringen, hat nur die Studienleitung Zugriff. Es existiert nur eine Kodierliste, die von der Studienleitung unter Verschluss gehalten wird.  Die Versuchsteilnehmer*innen haben das Recht auf Auskunft über alle ihre personenbezogenen Daten. Sie haben auch Anrecht auf Korrektur eventueller Ungenauigkeiten in ihren personenbezogenen Daten und können jederzeit der Weiterverarbeitung ihrer im Rahmen der Studie erhobenen Daten widersprechen und ihre Löschung bzw. Vernichtung verlangen. Dies ist so lange möglich, wie die Kodierliste existiert.  Alle Fragebögen/Interviews, Daten der elektronischen Fragebögen, der biologischen Proben, und der behavioralen Daten sowie dazugehörige Papierfragebögen werden ohne persönliche Identifizierungsmerkmale nur mit dem Code versehen aufbewahrt und sind ausschließlich Mitgliedern des Studienteams zugänglich.  Die genetischen Proben werden an ein Labor in München zu der Kooperationspartnerin des Projekts, Prof. Dr. Elisabeth Binder, für genetische und epigenetische Auswertungen gesendet. Die Proben werden ausschließlich in pseudonymisierter Form versendet.  Alle weiteren biologischen Proben werden in Gefrierschränken, die sich im Labor der Forschungsgruppe Soziale Neurowissenschaften der Max-Planck-Gesellschaft befinden, gelagert. Diese biologischen Proben werden im Institut für Medizinische Psychologie der Charité pseudonymisiert ausgewertet. Die Räume, in denen sich die Gefrierschränke befinden, sind verschlossen und nur Mitarbeiter*innen des Forschungslabors haben Zutritt.  Die Untersuchungsergebnisse werden elektronisch gespeichert und verarbeitet. Weitere Angaben zum Datenschutz sowie zur Aufbewahrung und Archivierung sind unter 26. und 27. zu finden. |
| 24. Ggf.: Beschreibung, wie der Gesundheitszustand gesunder betroffener Personen dokumentiert werden soll | Der Gesundheitszustand der Teilnehmer*innen wird im Prescreening mittels Fragebögen nach subjektiver Selbstauskunft erhoben (siehe **Anlage B**). |
| 25. Ggf.: Methoden, unerwünschte Ereignisse festzustellen, zu dokumentieren und mitzuteilen (wann, von wem und wie ?) | entfällt |
| 26. Vorgehen zum Schutz der Geheimhaltung der gespeicherten Daten, Dokumente und ggf. Proben, Darlegung der Pseudonymisierung oder Anonymisierung der Daten und Proben von Studienteilnehmern (Initialen und Geburtsdatum als Codierungsschema sind nicht zulässig!)  - Beschreibung der Trennung von Krankenakten, Studiendokumentation und Zuordnung der personenbezogenen Daten  - Nennung der Zugriffsrechte einschließlich des Zugangs zu Teilnehmeridentifikationslisten während und nach der Studiendurchführung  - Detaillierte Angabe der Verfahren für die Übertragung, Verschlüsselung, Einschränkung der Verarbeitung (Sperrung) und Löschung (einschließlich Angabe der ggf. verwendeten Netzstruktur und verwendete Server).  -ggf. Zugang zu identifizierenden Daten für gesetzlich berechtigte Prüfer (Dritte) zur zweckgebundenen Einsichtnahme in die dafür erforderlichen Akten. | Die für die Studie geltenden gesetzlichen Bestimmungen des Datenschutzes nach EU-DSGVO sowie des Berliner Landesdatenschutzgesetz sowie des Gendiagnostikgesetzes werden für alle personenbezogenen Daten erfüllt.  Da es sich um eine Fortführung des Forschungsprojekts CovSocial handelt, existieren bereits erhobene personenidentifizierende Daten. Diese umfassen E-Mail-Adressen, Namen sowie Adressen der Teilnehmer*innen. In diesem Teil der Studie werden daher nur die Bankdetails der Proband*innen für die Auszahlung der Aufwandsentschädigung neu erfragt. Dafür erhalten die Proband*innen über die Webapp und/oder per Email ein Formular zum Antrag auf Erstattung der Aufwandsentschädigung (**Anlage K**), das Sie mit Ihren Bankdetails ausfüllen und ausdrucken können. Das Formular bringen sie unterschrieben zur Präuntersuchung oder Postuntersuchung mit. Mit der Unterschrift bestätigen die Proband*innen die Richtigkeit der Angaben und erlauben die Weiterleitung dieser an die für die Forschungsgruppe Soziale Neurowissenschaften zuständige Buchhaltung. Die Weiterleitung erfolgt durch die administrativen Mitarbeiter*innen der Forschungsgruppe Soziale Neurowissenschaften.  In dem ersten Studienabschnitt des Projekts wurde bereits für jede*n Teilnehmer*in eine pseudonyme Identifikationsnummer generiert, die keinen Rückschluss auf die Identität der Studienteilnehmer*innen zulässt. In der Folge wird nur diese für die Kennzeichnung sämtlicher erhobener Daten verwendet. Die Identifikationsnummer und personenbezogene Informationen werden auf einem zugriffsgesicherten Server der MPG (Subserver 1) gespeichert. Die Datenerhebung und -speicherung erfolgt auf einem hiervon getrennten Server der MPG (Subserver 2), auf dem auch die Webanwendung installiert ist. Programmiert wurde die Webanwendung von der Digitalagentur CosmoCode, die von der Max-Planck-Gesellschaft beauftragt wurde. Die Speichelproben und Blutproben werden mit einem separaten Codeschlüssel versehen (dual coding). Die Kodierliste, die die Verbindung zwischen der Genetik-ID/Blut-ID/Kortisol-ID und der Studien-ID herstellen lässt, ist auf dem Subserver 1 des MPG gespeichert und nur bestimmten, von der Studienleitung festgelegten Person und der Studienleitung zugänglich. Die Kodierliste der Blut-ID, so wie die Kontaktdaten der Proband*innen werden über einen verschlüsselten Kanal dem Institut für Medizinische Psychologie zur Verfügung gestellt. Sie wird passwortgeschützt nur der administrativen Assistenz des Instituts, für die Terminplanung des Laborbesuchs zur Blutentnahme, zugänglich sein. Die Proben werden in den Laboren, in denen die Analyse stattfindet (Institut für Psychiatrie - MPG und Institut für Medizinische Psychologie - Charité) in zugriffssicheren Gefrierschränken in abschließbaren Räumen gelagert.  Der Zugriff auf und die Übertragung von ausschließlich pseudonymisierten studienbezogenen Daten von einem weiteren Server der MPG zu den Mitarbeitern*innen des Projekts CovSocial erfolgt über eine verschlüsselte Verbindung in das Wissenschaftsnetz. MPG-externe Kooperationspartner*innen an der Charité - Universitätsmedizin Berlin und der Humboldt-Universität zu Berlin können die pseudonymisierten Daten gemäß DSGVO-konformer Richtlinien an ihren jeweiligen Institutionen zu wissenschaftlichen Zwecken verwenden.  Die Daten werden pseudonymisiert ausgewertet und 10 Jahre gespeichert. Nach diesem Zeitraum wird die Kodierliste, die es erlaubt, die studienbezogenen Daten mit den personengebundenen und identifizierenden Daten in Verbindung zu bringen, gelöscht.  Ausgedruckte, schriftliche Unterlagen werden in abschließbaren Räumen und Schränken aufbewahrt, zu denen nur Studienbeteiligte Zugriff haben. Auch hierbei werden die personenbezogenen Daten getrennt von den Studiendaten aufbewahrt. Zugriff auf die personenidentifizierenden Daten haben nur von der Studienleitung auserwählte Mitarbeiter, die aber keinen Zugriff auf die Studiendaten haben. Zugriff auf die Studiendaten haben nur Anwender*innen mit entsprechender Berechtigung. Die Vergabe der Zugriffsrechte wird durch die Studienleitung an den jeweiligen Standorten an entsprechende Studienmitarbeiter*innen übertragen. |
| 27. Erklärung zur Einhaltung des Datenschutzes  - Zusicherung, dass alle über den Studienteilnehmer erhobenen und gespeicherten Daten vertraulich (Datengeheimnis und ärztliche Schweigepflicht) behandelt werden.  - Zusicherung, dass die identifizierenden Daten nur dem Studienleiter oder von ihm beauftragten Mitarbeitern zugänglich sind.  - Angabe der Maßnahmen zur Sicherstellung der Vertraulichkeit  - Maßnahmen zur datenschutzgerechten Übermittlung von Daten, die für Dritte keinen Personenbezug herstellen lassen.  - Angaben zu Auskunfts-, Widerrufs-, Berichtigungs- und Löschmöglichkeiten,  - Maßnahmen zur Sicherstellung der Rechte der Teilnehmer.  - Falls Übermittlungen ins Nicht-EU-Ausland vorgesehen sind: Maßnahmen zur Einhaltung des Datenschutzes (z.B. Vorliegen eines Angemessenheitsbeschlusses der EUKommission oder explizite Einwilligung der Studienteilnehmer in solche Übermittlungen | Der Schutz persönlicher Daten aller Studienteilnehmer*innen wird gewährleistet. Die Verarbeitung und Veröffentlichung von Daten erfolgten ausschließlich in pseudonymisierter Form.  Entsprechend der Datenschutzgesetzgebung werden sämtliche Daten vertraulich behandelt. Sämtliche Daten werden physisch oder logisch zugriffsgeschützt (wie unter 26 beschrieben).  Zwischen der Max-Planck-Gesellschaft als Eigentümerin der in diesem Projekt verwendeten WebApp bzw. mobilen App und dem Digitaldienstleister CosmoCode besteht ein Vertrag zur Auftragsdatenverarbeitung (AVV) gemäß Art. 28 DSGVO, der die technischen und organisatorischen Maßnahmen des Dienstleisters im Sinne des Art. 32 DSGVO regelt.  Zwischen der Max-Planck-Gesellschaft und den für die Studie eingestellten MBSR-Trainer*innen, die mit den Teilnehmer*innen ein telefonisches Screening im Einzelgespräch und wöchentliche Gruppen-Monitoring-Konferenzen in Gruppen (à 20-25 Personen) durchführen, ist die Schweigepflicht vertraglich geregelt. Außerdem besteht ein Vertrag zur Auftragsdatenverarbeitung (AVV) gemäß Art. 28 DSGVO, der die technischen und organisatorischen Maßnahmen des Dienstleisters im Sinne des Art. 32 DSGVO regelt.  Die Studienteilnehmer*innen erhalten die Möglichkeit, jederzeit (auch nach der Befragung) ihre Zustimmung zur Teilnahme an der Studie zu widerrufen, ohne dass daraus Nachteile resultieren. In diesem Falle werden sämtliche personenidentifizierende Daten gelöscht.  Die Proband*innen haben jederzeit die Möglichkeit, mit dem Studien-Personal Kontakt aufzunehmen.  Zur vollständigen Information, siehe Teilnahmeinformation (**Anlage H**) und Einwilligungserklärung (**Anlage J**). |
| 28. Namen und Anschriften der Einrichtungen, die als Studienzentrum oder Studienlabor in die Studie eingebunden sind, sowie der Studienleiter und der Studienärzte  - Angabe beteiligter externer Dienstleister mit Angabe der Datenzugriffsmöglichkeit | **Studienzentren**  Charité – Universitätsmedizin Berlin  Institut für Medizinische Psychologie  Campus Charité Mitte  Luisenstraße 57  10117 Berlin  Forschungsgruppe Soziale Neurowissenschaften  Max-Planck-Gesellschaft  Campus Nord, Haus 5  Humboldt-Universität zu Berlin  Philippstrasse 13  10099 Berlin  **Studienleiterin CovSocial Projekt**  Prof. Dr. Tania Singer (Gastwissenschaftlerin)  Charité – Universitätsmedizin Berlin  Klinik für Psychiatrie und Psychotherapie  Campus Charité Mitte  Charitéplatz 1  10117 Berlin  tania.singer@charite.de  Prof. Dr. Tania Singer (wissenschaftliche Leiterin)  Forschungsgruppe Soziale Neurowissenschaften  Max-Planck-Gesellschaft  Campus Nord, Haus 5  Humboldt-Universität zu Berlin  Philippstrasse 13  10099 Berlin  E-Mail: singer@social.mpg.de  **Teilleitung Phase 2 stressbedingte biologische Marker**  Prof. Dr. Sonja Entringer  Charité – Universitätsmedizin Berlin  Institut für Medizinische Psychologie  Luisenstraße 57  10117 Berlin  E-Mail: [sonia.entringer@charite.de](mailto:sonia.entringer@charite.de)  Prof. Dr. Christine Heim (Teilleitung Phase 2 stressbedingte biologische Marker)  Charité – Universitätsmedizin Berlin  Institut für Medizinische Psychologie  Luisenstraße 57  10117 Berlin  E-Mail: [christine.heim@charite.de](mailto:christine.heim@charite.de)  **Weitere Kooperationspartner*innen**  Prof. Dr. med. Mazda Adli (Phase 1)  Charité – Universitätsmedizin Berlin  Klinik für Psychiatrie und Psychotherapie  Campus Charité Mitte  Charitéplatz 1  10117 Berlin  E-Mail: mazda.adli@charite.de  Prof. Dr. Manuel Voelkle  Humboldt-Universität zu Berlin  Lebenswissenschaftliche Fakultät  Institut für Psychologie  Unter den Linden 6  10099 Berlin  E-Mail: manuel.voelkle@hu-berlin.de  Prof. Dr. Elisabeth Binder  Max-Planck-Institut für Psychiatrie  Kraepelinstr. 2 - 10  80804 München  E-Mail: [binder@psych.mpg.de](mailto:binder@psych.mpg.de)  **Externe Dienstleister**  CosmoCode GmbH  Prenzlauer Allee 36G  10405 Berlin  Telefon: +49 30 814 50 40 70  Telefax: +49 30 2809 7093  Mail: [info@cosmocode.de](mailto:info@cosmocode.de) |
| 29. Angaben zur Eignung der Prüfstelle, insbesondere zur Angemessenheit der dort vorhandenen Mittel und Einrichtungen sowie des zur Durchführung der klinischen Prüfung zur Verfügung stehenden Personals und zu Erfahrungen in der Durchführung ähnlicher Studien | Die an der Studie beteiligten Personen und Professor*innen verfügen über langjährige Expertise auf dem Gebiet der psychobiologischen Forschung und der Durchführung von Interventionsstudien, was sich durch vielfältige Publikationen, Buchbeiträge und Vortragsveranstaltungen aller Partner*innen dokumentieren lässt. |
| 30. Vereinbarung über den Zugang des Prüfers/Hauptprüfers/Leiters der klinischen Prüfung, zu den Daten und den Grundsätzen über die Publikation.  - Publikationen in einer Form, die keinen Rückschluss auf die Person zulässt. | Die Veröffentlichung von Ergebnissen erfolgt ausschließlich in pseudonymisierter Form. |
| 31. Angaben zur Finanzierung der Studie: Finanzierungsquelle (Name und Sitz) und Höhe der Förderung in €.  -ggf. Angabe der Kostenstelle zur ILV Abrechnung der Gebühr | Finanzierung durch:  Forschungsgruppe Soziale Neurowissenschaften  Max-Planck-Gesellschaft  Campus Nord, Haus 5  Humboldt-Universität zu Berlin  Philippstrasse 13  10099 Berlin  Charité – Universitätsmedizin Berlin  Institut für Medizinische Psychologie  Luisentraße 57  10117 Berlin  Max-Planck-Institut für Psychiatrie  Kraepelinstr. 2 - 10  80804 München |

Prof. Dr. Tania Singer Prof. Dr. Sonja Entringer Prof. Dr. Christine Heim

Studienleitung Teilleitung Bereich stressbedingte Teilleitung Bereich stressbedingte biologische Marker biologische Marker
