# Supplementary material for: Investigating differential effects of socio-emotional and mindfulness-based online interventions on mental health, resilience and social capacities during the COVID-19 pandemic: The study protocol
Source: PLoS One. 2021 Nov 4;16(11):e0256323. doi: 10.1371/journal.pone.0256323 (PMC8568275; doi:10.1371/journal.pone.0256323)
Supplement: S4 File — (DOCX) [file pone.0256323.s004.docx]

Berlin, 20.07.2021

**Translation of Amendment to Ethics Proposal by Social Neuroscience Lab, Max Planck Society**

**In this amendment, highlighted portions refer to additions to the original ethics proposal, and portions with strikethrough refer to deletions from the original ethics proposal.**

| Title of the Study | **CovSocial Phase 2 – Investigating differential effects of mindfulness-based online interventions on mental wellbeing and social cohesion** |
| --- | --- |
| 1. Decisions of other ethics committees on the same topic | Not applicable |
| 2. Subject of the study and objectives; Specification of hypotheses, divided into main and secondary hypotheses like the clinical parameter (primary and secondary endpoints), against which the hypotheses are to be tested. | **The proposed study aims to investigate and compare the effectiveness of two online mental health interventions in reducing stress and loneliness and increasing psychological well-being, prosocial behaviour and compassion. The study presented here serves as phase 2 of the CovSocial project, which aims to investigate changes in mental health and social cohesion over the period of the SARS-CoV-2 pandemic in a large sample of Berlin citizens. In phase 1 (EA4/172/20), personality and behavioural traits were identified that were associated with higher vulnerability to stress and mental health or lower resilience (state) during the SARS-CoV-2 pandemic. In addition, state measures of the relevant factors were collected for 7 time points (T1-T7) between January 2020 and March 2021. So-called polygenic risk scores (genetic and epigenetic) were also collected in a subsample during the first phase of the project, which could influence individual coping patterns (Wray et al., 2020).** These reflect the aggregate genetic risk for a specific illness (e.g. mental illnesses such as depression or anxiety disorders, etc.) or correlate with the expression of certain phenotypes and behavioural patterns (e.g. neuroticism, optimism, reward system, etc.) that could be important for the individual expression of stress, resilience and social cohesion. The calculation of these polygenic risk scores is based on the results of large studies, often from international consortia and large biobanks. The predictive value of these scores increases with the size of the cohorts and meta-analyses and these scores must therefore always be adapted to the latest literature.  Phase 2 consists of exploring mindfulness-based mental intervention programmes in a subgroup of the probands studied in phase 1. These intervention programmes were based on a mental training programme, the ReSource project (Singer et al., 2016), which included these mental interventions as elements. The effectiveness on stress reduction and an increase in prosocial behaviour through this programme has been demonstrated in numerous studies (Hildebrandt et al, 2017; Singer & Engert, 2019). In the planned intervention study phase 2 of the CovSocial project, the mental training exercises will now be studied without the context of other intervention elements. Thus, the scalability of short online interventions will be investigated.  Specifically, two different intervention groups will be compared with each other and with a retest control group using pre- and post-tests and daily or weekly online app-based ambulatory measurement protocol ("ecological momentary-assessment (EMA)") questions. One training group underwent a classic multi-week mindfulness-based meditation programme and the other group underwent an equally long dyad programme based on short daily partner exercises (Kok & Singer, 2017). Both mental training programmes have been shown in previous research to similarly reduce stress and loneliness and to increase social skills and prosociality (Singer & Engert, 2019). As dependent variables, the focus is on recording changes in stress experience, experienced loneliness, resilience, mental health as well as social proximity, social cohesion and social skills such as (self-)compassion and empathy. In addition, longitudinal data from phase 1 will be used to predict both pre-training and training-related effects in phase 2.  The SARS-CoV-2 pandemic is a global challenge with profound implications for health systems, economics and social life. Given the government restrictions, combined with the fear of becoming infected and the risk of losing one's job, the situation is now seen as a stressful life event for most of the population (Horesh & Brown, 2020). This creates a need to examine the psychological consequences of the pandemic and its associated lockdown, as well as to identify individuals who are at the greatest risk of developing mental health problems after experiencing COVID-19-related stress. The intervention aims to support these individuals in reducing stress and increasing their psychological well-being. In future, individuals at increased risk for high-stress levels can be identified at an early stage. Thus, severe stress reactions and the occurrence of mental disorders can be prevented prospectively.  Studies on the psychological consequences of pandemics have shown that quarantine and isolation regimes in particular have resulted in an increase in stress, anxiety and nervousness and depression (Brooks et al., 2020). In a study of the 2002/2003 SARS pandemic, social isolation was the largest factor in subsequent severe stress reactions (Bai et al., 2004). Since the repeated phases of lockdowns in Berlin since March 2020 in particular were also primarily characterised by the appeal for self-isolation and contact minimisation, it can be assumed that many people were also affected by increased stress during this time. Other stress factors in the pandemic include various individual challenges such as fear of unemployment, concerns about health or the increased childcare burden due to the closure of schools and kindergartens. Due to the social isolation, high uncertainty, individual problems as well as the potentially damaging, threatening or challenging effects of the COVID-19 pandemic, it can be assumed that as a result of the pandemic, stress in the population has risen sharply. This assumption is confirmed by initial results of studies on the effects of the COVID-19 pandemic on mental health. In Germany, an increase in anxiety disorder symptoms, depression and psychological stress was found (Bäuerle et al., 2020). This increase was mainly predicted by the extent of restriction of social contacts and the severity of the change in everyday life (Benke et al., 2020).  Stress exposure is associated with activation of stress-related biological regulatory systems, such as the hypothalamic-pituitary-adrenal axis (HPA axis) and the sympatho-adrenergic system. If the stress load is chronic, as during the ongoing covid pandemic, the prolonged activation of these biological stress axes can lead to changes in neuroendocrine, immunological, metabolic regulatory circuits, and cellular ageing processes, which in turn underlie the increased risk of physical and mental illness associated with chronic stress load (Cohen et al., 2012). It is known that life-history early stress experiences increase the risk for stress-related diseases. Stress during sensitive periods of development leads to biological embedding at the epigenetic level as well as changes in neural and peripheral regulatory systems, which ultimately contribute to disease risk, especially after renewed stress exposure later in life (vulnerability model; Heim et al., 2019). A further aim of this study is therefore to investigate the relationship between chronic stress exposure during the COVID-19 pandemic and changes in stress-related biological systems, as well as to identify factors that increase vulnerability to changes in stress-associated biological systems during the COVID-19 pandemic.  These biological changes can be embedded over time via transcription factor activation by altering gene regulation and expression through changes in the epigenome (Klengel & Binder, 2015). Epigenetic changes alter gene transcription not by changing the genome sequence, but by changing its accessibility to transcriptional regulators (e.g. DNA methylation, histone modifications; Aristizabal et al., 2019). In the case of chronic stress, stress hormones or glucocorticoids, which can act as transcription factors and induce permanent epigenetic changes, are likely to play a prominent role in these changes, as shown by the overlap of the epigenetic effects of chronic stress and glucocorticoids, as well as the enrichment of the DNA methylation site of the glucocorticoid response in stress-induced epigenetic changes (Zannas et al., 2015). These environmental epigenetic changes are influenced by genetic factors, so genes, environment and epigenetics should be studied together (Czamara et al., 2019). Epigenetic markers can also both predict the success of therapeutic interventions and be altered by such interventions in the long term (Schiele, Gottschalk & Domschke, 2020; Goud Alladi et al., 2018; Vinkers et al., 2019).  For the study, data will be collected from a total of 300 subjects (100 subjects per group) before, during and after the planned intervention. Subjects will be allocated to three groups using stratified randomisation. The subjects will each undergo 10 weeks of intervention training. Group I will complete the 12-minute affect dyad six times per week (Kok & Singer, 2017). This daily app-based online partner exercise consists of a person speaking (6 minutes) of a non-judgmental description of stressful and grateful situations they were in during the last day and the effect on their body sensation (interoceptive body awareness). The person listening (6 minutes) trains non-judgmental empathic listening. After half the time, the roles are changed. Group II will do 12-minute mindfulness meditations and breathing meditations six times a week. Group III will serve as a retest control group with a waiting list condition and, after post-testing the three groups, will now also do the 12-minute affect dyad six times a week, as group I did before. Groups I and II will also be offered to continue the respective mindfulness-based intervention practised via the app after the post-testing. Depending on the level of drop-out rate as well as funding possibilities, a follow-up measurement of groups I and II will also take place. A graphical representation of the study design can be found in Figure 1.  **Figure 1**: *Study plan of phase 2 in the CovSocial project*.  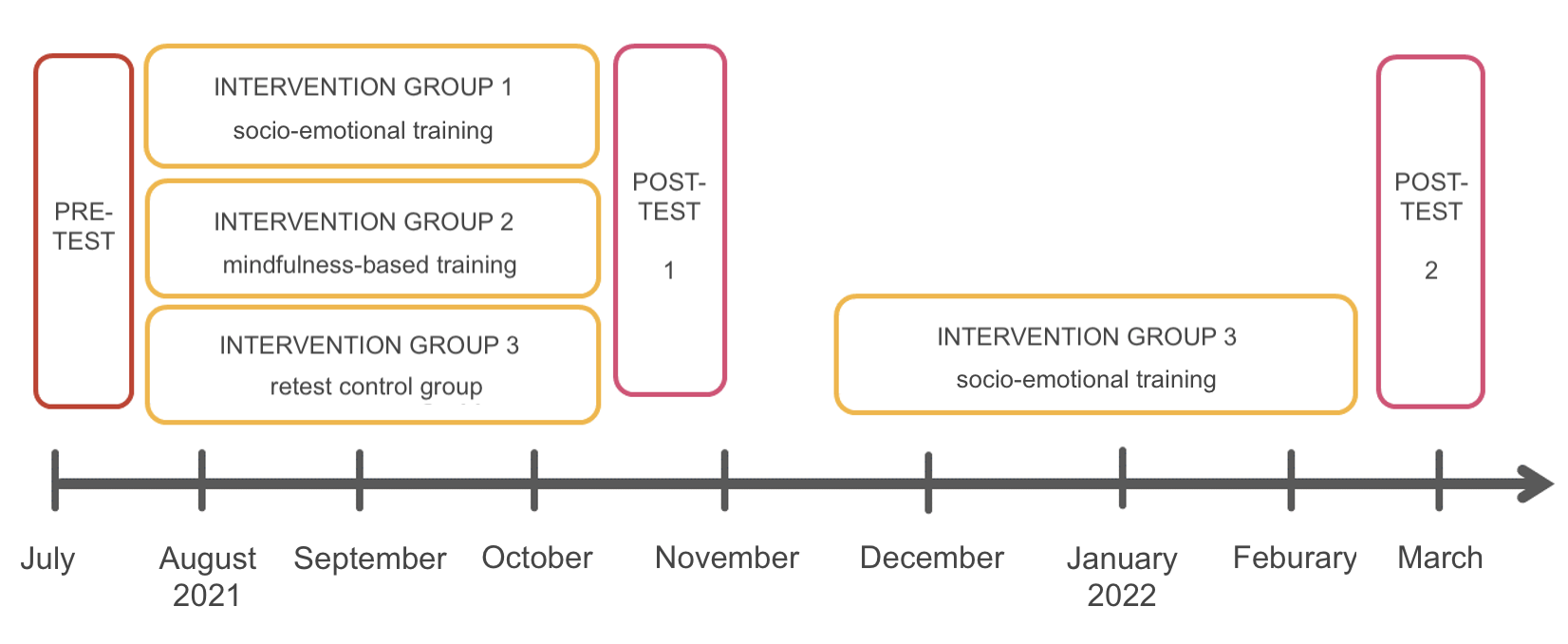  Psychological, behavioural and biological parameters will be collected from all probands before and after the intervention. Psychological data will be collected with online questionnaires and an ambulatory measurement protocol ("ecological momentary assessment (EMA)"). Data will be collected in real-time during the subjects' usual everyday life. Biological markers include neuroendocrine, ~~immunological and metabolic~~ parameters, as well as ~~indicators of cellular ageing processes~~ and genome-wide epigenetic markers. They will be collected through a ~~pre-intervention blood sample~~ and pre and post-intervention saliva samples. Behavioural data will be collected using computer-based experiments. During the respective intervention, further psychological data will also be collected via the smartphone-based procedure of the EMA.  The following hypotheses are tested:  H1: We expect intervention effects on the stress markers collected from saliva samples, EMA measurements and questionnaires.  H1a: For individuals undergoing the socio-emotional or mindfulness training, we expect a greater reduction in subjectively experienced stress compared to the retest control group, as measured by the PSS-10, at post-test compared to the pre-test.  H1b: We expect a reduction in Cortisol Awakening Response (CAR) for individuals undergoing the socio-emotional training compared to those undergoing the mindfulness-based training and to the test-retest control group. We also expect this reduction to be mediated by an increase in acceptance as stress coping strategies as measured by the CERQ.  H1c: Individuals who have higher scores on psychological resilience markers (collected in phase 1 of the project) and report a higher increase in subjective stress experience during the pandemic (collected in phase 1 of the project) show a greater reduction in subjective stress experience after socio-emotional or mindfulness-based training, as measured by the PSS-10 than individuals with lower scores on psychological resilience markers or lower increase in subjective stress experience during the pandemic.  H1d: Individuals who have higher scores on psychological resilience markers (collected in phase 1 of the project) and report a higher increase in subjective stress experience during the pandemic (collected in phase 1 of the project) show a greater reduction in the Cortisol Awakening Response (CAR) after socio-emotional training than individuals with lower scores on psychological resilience markers or lower increase in subjective stress experience during the pandemic.  H2: We expect intervention effects on subjective loneliness.  H2a: We expect a reduction from pre-test to post-test in subjective loneliness as measured by the UCLA for individuals undergoing socio-emotional training, but not for those undergoing mindfulness-based training or in the test-retest control group.  H2b: We expect an increase in subjectively perceived social closeness for individuals who undergo the socio-emotional training, but not for those who undergo the mindfulness-based training. Furthermore, we expect an increase in shared personal information over the course of the socio-emotional training.  H2c: The reduction in subjectively perceived loneliness is associated with an increase in subjectively perceived social closeness, measured with the IOS before and after the socio-emotional training, as well as an increase in personal information shared in the dyad (personal disclosure).  H3: We expect intervention effects on mental health.  H3a: We expect a greater reduction in the severity of subclinical depressive symptoms, as measured by the BDI-II, for individuals who undergo the socio-emotional training than for those who undergo the mindfulness-based training or belong to the test-retest control group. We also expect a greater reduction in the expression of subclinical depressive symptomatology for individuals who undergo the mindfulness-based training than for those who belong to the test-retest control group.  H3b: We expect that a reduction in the expression of subclinical depressive symptomatology, as measured by the BDI-II, from pre-test to post-test for individuals undergoing the socio-emotional training will be related to increased use of acceptance as a stress coping strategy, as measured by the CERQ, over the course of the intervention, and for individuals undergoing the mindfulness-based training will be related to decreased use of rumination as a coping strategy, as measured by the CERQ, over the course of the intervention.  H3c: We expect a greater reduction in the expression of subclinical anxiety symptoms, as measured by the STAI, from pre-test to post-test for individuals undergoing the socio-emotional training than for those undergoing the mindfulness-based training or in the retest control group. We also expect a greater reduction in the expression of subclinical anxiety symptoms for individuals undergoing the mindfulness-based training than for those in the test-retest control group.  H3d: We expect that scores on psychological resilience and vulnerability markers (collected in phase 1 of the project), both at the trait level and in terms of their change during the pandemic in 2020 and early 2021, will predict the magnitude of training-induced changes in the expression of subclinical anxiety symptoms (STAI) and depressive symptoms (BDI) from pre-test to post-test.  H4: We expect intervention effects on psychological resilience.  H4a: We expect a greater increase in psychological resilience, as measured by the BRS, for individuals undergoing the socio-emotional training than for those undergoing the mindfulness-based training or in the retest control group. We also expect a greater increase in psychological resilience for individuals who undergo the mindfulness-based training than for those in the retest control group.  H4b: We expect the increase in psychological resilience (BRS) from pre-test to post-test for individuals undergoing the socio-emotional training to be related to an increased use of acceptance and social support as coping strategies, as measured by the CERQ, as well as an increase in psychological flexibility, ~~as measured by the Affective Flexibility Task~~, over the course of the intervention. For individuals undergoing the mindfulness-based training, we expect the increase in psychological resilience from pre-test to post-test to be related to a reduction in rumination (CERQ) and an increase in acceptance (CERQ).  H4c: We expect that scores on psychological resilience and vulnerability markers (collected in phase 1 of the project), both at the trait level and in terms of their change during the pandemic in 2020 and early 2021, can predict the magnitude of training-induced changes in psychological resilience from pre-test to post-test.  H5: We expect intervention effects on empathy and compassion.  H5a: We expect a greater increase in empathy and compassion, as measured by the EmpaToM, the IRI and the SCS and the SOCS, for individuals undergoing the socio-emotional training than for those undergoing the mindfulness-based training or in the retest control group. We also expect a greater increase in empathy, but not in compassion, for individuals undergoing the mindfulness-based training than for those in the retest control group. We do not expect changes in empathy and compassion in the test-retest control group from pre-test to post-test. In addition, we do not expect any change in the Theory of Mind or cognitive perspective-taking in any group.  H5b: We expect increases in empathy in both intervention groups to be mediated by increases in interoceptive awareness (measured with EMA questions before and after training), mindfulness (measured with the MAIA and CAMS-R), and increased use of acceptance as a coping strategy.  H5c: We expect increases in compassion from pre-test to post-test to be mediated by a reduction in fear of compassion, an increase in self-compassion (SCS subscale self-kindness), a reduction in personal distress (IRI subscale personal distress), an increase in acceptance (CERQ) and positive feelings (Affect Grid).  H5d: Based on positive associations between social skills and adaptive coping strategies (found in Phase 1 of the project), we expect that the use of adaptive coping strategies elicited in Phase 1 will predict greater training-induced increases in empathy and compassion.  H5e: We further expect that scores on psychological social cohesion and vulnerability markers (collected in phase 1 of the project), both at the trait level and in terms of their change during the pandemic in 2020 and early 2021, will predict the strength of training-induced changes in empathy and compassion.  H6: We expect the intervention to have effects on prosocial behaviour.  H6a: We expect a greater increase in altruistic prosocial behaviour, as measured by the PSA and a battery of behavioural tests of altruistic prosocial behaviour (Böckler et al., 2018), for individuals undergoing the socio-emotional training than for those undergoing the mindfulness-based training or in the retest control group. We do not expect changes in prosocial behaviour from pre-test to post-test for individuals who undergo the mindfulness-based training or belong to the retest control group.  H6b: We expect an increase in altruistic prosocial behaviour to be predicted by increases in empathy and compassion. Furthermore, we expect that a reduction in fear of expressing compassion towards others, as measured by the Fear of Compassion Scale, may predict individual differences in training-induced increases in prosocial behaviour.  H6c: We further expect that scores on psychological social cohesion and vulnerability markers (collected in phase 1 of the project), both at the trait level and in terms of their change during the pandemic in 2020 and early 2021, will predict the strength of training-induced changes in prosocial behaviour.  **Hypotheses on inflammatory markers in saliva**  H7a: Perceived stress during the pandemic (phase 1) is associated with inflammatory markers (CRP) at the time of the pretest.  H7b: These stress-associated inflammatory markers mediate the association between subjective feelings of stress during the pandemic and mental health at the time of the pretest  H8a: There are interindividual differences in the strength of the association between subjective stress (phase 1) during the pandemic and CRP levels as a function of childhood stress (assessed in phase 1 with the Childhood Trauma Questionnaire).  H8b: Individuals with high early childhood stress levels show greater mental health impairment at the time of the pretest.  **~~Hypotheses on stress biological systems from blood samples~~**  ~~H7a: Perceived stress during the pandemic (phase 1) is associated with the activation of stress biological systems.~~  ~~H7b: This is associated with immunological and metabolic as well as changes in cellular ageing processes.~~  ~~H7c: These stress-associated biological markers mediate the association between subjective feelings of stress during the pandemic and mental and physical health at the time of the pre-test.~~  ~~H8: There are interindividual differences in the strength of the association between subjectively experienced stress (phase 1) during the pandemic and changes in stress-biological systems as a function of childhood stress (assessed in phase 1 with the Childhood Trauma Questionnaire).~~  ~~H8a: Individuals with high early childhood stress (assessed in phase 1 with the Childhood Trauma Questionnaire) show a higher degree of dysregulation in stress-biological parameters at the time of the pre-test.~~  ~~H8b: Individuals with high levels of early childhood stress show greater impairment in mental and physical health at the time of the pre-test.~~  ~~H8c: These stress-associated biological markers mediate the association between high-stress levels during childhood and mental and physical health at the time of the pre-test.~~  **Hypotheses on genetic and epigenetic markers**  H9: The influence of polygenic and polyepigenetic risk scores on training-related intervention effects should be investigated.  H9a: It should be investigated whether polygenic risk scores collected in phase 1 can predict the effects of the intervention mentioned in hypotheses 1-6.  H9b: It should be investigated whether polyepigenetic risk scores collected in phase 1 can predict the effects of the intervention mentioned under hypotheses 1- 6.  H10: It should be investigated whether the socio-emotional and mindfulness-based interventions have a positive effect on polyepigenetic risk scores in the post-test compared to the pre-test.  *Literature:*  Bai, Y., Lin, C. C., Lin, C. Y., Chen, J. Y., Chue, C. M., & Chou, P. (2004). Survey of stress reactions among health care workers involved with the SARS outbreak. *Psychiatric Services*, *55*(9), 1055-1057.  Bäuerle, A., Teufel, M., Musche, V., Weismüller, B., Kohler, H., Hetkamp, M., ... & Skoda, E. M. (2020). Increased generalized anxiety, depression and distress during the COVID-19 pandemic: a cross-sectional study in Germany. *Journal of Public Health*, *42*(4), 672-678.  Benke, C., Autenrieth, L. K., Asselmann, E., & Pané-Farré, C. A. (2020). Lockdown, quarantine measures, and social distancing: Associations with depression, anxiety and distress at the beginning of the COVID-19 pandemic among adults from Germany. *Psychiatry research*, *293*, 113462.  Brooks, S. K., Webster, R. K., Smith, L. E., Woodland, L., Wessely, S., Greenberg, N., & Rubin, G. J. (2020). The psychological impact of quarantine and how to reduce it: rapid review of the evidence. *The lancet*, *395*(10227), 912-920.  Cohen, S., Janicki-Deverts, D., Doyle, W. J., Miller, G. E., Frank, E., Rabin, B. S., & Turner, R. B. (2012). Chronic stress, glucocorticoid receptor resistance, inflammation, and disease risk. *Proceedings of the National Academy of Sciences*, *109*(16), 5995-5999.  Czamara, D., Eraslan, G., Page, C.M., Lahti, J., Lahti-Pulkkinen, M., Hämäläinen, E., Kajantie, E., Laivuori, H., Villa, P.M., Reynolds, R.M., Nystad, W., Håberg, S.E., London, S.J., O'Donnell, K.J., Garg, E., Meaney, M.J., Entringer, S., Wadhwa, P.D., Buss, C., Jones, M.J., Lin, D.T.S., MacIsaac, J.L., Kobor, M.S., Koen, N., Zar, H.J., Koenen, K.C., Dalvie, S., Stein, D.J., Kondofersky, I., Müller, N.S., & Theis, F.J. (2019). Major Depressive Disorder Working Group of the Psychiatric Genomics Consortium, Räikkönen, K., Binder, E.B. Integrated analysis of environmental and genetic influences on cord blood DNA methylation in new-borns. Nature Communications, 10(1), 2548. doi: 10.1038/s41467-019-10461-0. PMID: 31186427; PMCID: PMC6559955.  Goud Alladi, C., Etain, B., Bellivier, F., & Marie-Claire, C. (2018). DNA methylation as a biomarker of treatment response variability in serious mental illnesses: a systematic review focused on bipolar disorder, schizophrenia, and major depressive disorder. International journal of molecular sciences, 19(10), 3026.  Heim, C. M., Entringer, S., & Buss, C. (2019). Translating basic research knowledge on the biological embedding of early-life stress into novel approaches for the developmental programming of lifelong health. Psychoneuroendocrinology, 105, 123-137.  Hildebrandt, L. K., McCall, C., & Singer, T. (2017). Differential effects of attention-, compassion-, and socio-cognitively based mental practices on self-reports of mindfulness and compassion. *Mindfulness, 8(6),* 1488-1512.  Horesh, D., & Brown, A. D. (2020). Traumatic stress in the age of COVID-19: A call to close critical gaps and adapt to new realities. *Psychological Trauma: Theory, Research, Practice, and Policy*, *12*(4), 331.  Klengel, T., & Binder, E.B. (2015). Epigenetics of Stress-Related Psychiatric Disorders and Gene × Environment Interactions. Neuron, 86(6), 1343-57.  Kok, B. E., & Singer, T. (2017). Effects of contemplative dyads on engagement and perceived social connectedness over 9 months of mental training: A randomized clinical trial. *JAMA Psychiatry,* *74*(2), 126−134.  Schiele, M.A., Gottschalk, M.G., & Domschke, K. (2020). The applied implications of epigenetics in anxiety, affective and stress-related disorders – A review and synthesis on psychosocial stress, psychotherapy and prevention. Clinical Psychology Review, 77, <https://doi.org/10.1016/j.cpr.2020.101830>  Singer, T., & Engert, V. (2019). It matters what you practice: Differential training effects on subjective experience, behavior, brain and body in the *ReSource Project*. *Current Opinion in Psychology, 28,* 151–158.  Singer, T., Kok, B. E., Bornemann, B., Zurborg, S., Bolz, M., & Bochow, C. (2016). *The ReSource Project: Background, design, samples, and measurements*. Max Planck Institute for Human Cognitive and Brain Sciences, Leipzig.Smith, B. W., Dalen, J., Wiggins, K., Tooley, E., Christopher, P., & Bernard, J. (2008). The brief resilience scale: assessing the ability to bounce back. *International journal of behavioral medicine*, *15*(3), 194-200.  Vinkers, C. H., Geuze, E., van Rooij, S. J., Kennis, M., Schür, R. R., Nispeling, D. M., ... & Boks, M. P. (2019). Successful treatment of post-traumatic stress disorder reverses DNA methylation marks. Molecular psychiatry, 1-8.  Wray, N.R., Lin, T., Austin, J., McGrath, J.J., Hickie, I.B., Murray, G.K., & Visscher, P.M. (2020). From Basic Science to Clinical Application of Polygenic Risk Scores: A Primer. JAMA Psychiatry, doi: 10.1001/jamapsychiatry.2020.3049. Epub ahead of print. PMID: 32997097.  Zannas, A.S., Arloth, J. Carrillo-Roa, T., Iurato, S., Röh, S., Ressler, K.J., Nemeroff, C.B., Smith, A.K., Bradley, B., Heim, C., Menke, A., Lange, J.F., Brückl, T., Ising, M., Wray, N.R., Erhardt, A., Binder, E.B., & Mehta, D. (2018). Lifetime stress accelerates epigenetic aging in an urban, African American cohort: relevance of glucocorticoid signaling. Genome Biology, 16, 266. doi: 10.1186/s13059-015-0828-5. Erratum in: Genome Biology, 19(1), 61. PMID: 26673150; PMCID: PMC4699359. |
| 3. Explanation of the significance of the study | The importance of the study is based on the need for scientific investigation and validation of online interventions that effectively reduce stress and increase psychological well-being and social cohesion. The SARS-CoV-2 pandemic represents a major stressor due to the massive social changes and measures such as "physical distancing", which not only increases risk factors for mental disorders such as social stress, perceived loneliness or increased anxiety on an individual level but also changes crucial foundations of togetherness and social cohesion on a societal level.  To counter these potentially negative effects of this SARS-CoV-2 pandemic at the mental level, the online app-based 10-week socio-emotional training (affect dyads) and the mindfulness-based training (breathing meditation) of equal duration will be used to test, whether a) these mental programmes are time-efficient and digitally feasible and b) previous positive effects on stress reduction and increase of mental as well as social well-being, which are observed in much more intensive non-online training programmes lasting several months, such as the ReSource project (Singer et al. , 2016) (Engert & Singer, 2019), can also be observed when the mental programmes are shortened (to only 10 weeks), only online via the app (without introductory retreats and without weekly real sessions with teachers), and in a very reduced form (focus on one 10-minute exercise per day). Although previous research on affective dyads has shown that this intervention increases the subjective sense of social belonging, the stress-reducing and social competence and social cohesion-promoting effect have so far only been shown in the context of daily practice of other mental exercises and this over several months (Engert et al., 2017; Böckler et al., 2018). This intervention will now be applied in the context of the increased stress caused by the changes resulting from the SARS-CoV-2 pandemic. This should allow us to test whether such mental training programmes could also be used globally in a scalable way as online programmes without large daily requirements, to counteract the increased numbers of stress and mental illnesses in the population.  The findings from this study serve both to develop preventive intervention measures to reduce loneliness and stress in a digital format that is flexible in terms of time and placeand to investigate the extent to which specific risk groups can particularly benefit from the intervention.  An understanding of the basic biological mechanisms associated with an increased risk of disease following chronic stress exposure could support the development of individualised, mechanism-based forms of therapy aimed at prevention or reversibility with biological integration following stressful experiences.  *Literature:*  Engert, V., Kok, B. E., Papassotiriou, I., Chrousos, G. P., & Singer, T. (2017). Specific reduction in cortisol stress reactivity after social but not attention-based mental training. *Science Advances*, *3*(10), e1700495.  Böckler, A., Tusche, A., Schmidt, P., & Singer, T. (2018). Distinct mental trainings differentially affect altruistically motivated, norm motivated, and self-reported prosocial behaviour. *Scientific reports*, *8*(1), 1-14.  Singer, T., Kok, B. E., Bornemann, B., Zurborg, S., Bolz, M., & Bochow, C. (2016). *The ReSource Project: Background, design, samples, and measurements*. Max Planck Institute for Human Cognitive and Brain Sciences, Leipzig.Smith, B. W., Dalen, J., Wiggins, K., Tooley, E., Christopher, P., & Bernard, J. (2008). The brief resilience scale: assessing the ability to bounce back. *International journal of behavioral medicine*, *15*(3), 194-200. |
| 4. Which of the following regulations apply  Application  a) Medical Devices Act  according to § 23b MPG - exception of clin.  Examination  b) Radiation Protection Act and  Radiation Protection Ordinance  c) Gene Diagnostics Act  d) Data protection laws:  - Specific indication of the  responsible body to comply with  Data Protection Act (for Charité = -EU Data Protection Regulation (DSGVO), Berlin Data Protection Act - BlnDSG). - If applicable, additional state data protection laws or BDSG to be observed according to the group of participants. | The following regulations apply in this study:   - Data protection laws:   - EU-DSGVO   - Berlin Data Protection Act - Gene Diagnostics Act |
| 5. If applicable: designation and characterisation of the test products | Not applicable |
| 6. Significant results of the pre-clinical tests or reasons for not carrying them out. | Not applicable |
| 7. Essential content and results of previous studies/applications of the products to be tested in the study | Not applicable |
| 8. Description of the planned measures/examination methods and any deviations from the measures/examinations usual in medical practice (what is "routine", what is done differently in the study?) If validated questionnaires are used for study purposes, please state the name of the questionnaires and where they are published (references). Please attach non-validated questionnaires. | Two mental training programmes (mindfulness-based and socio-emotional dyad training) will be compared with each other and with a re-test control group.  From the N = 3522 participants who fully participated in phase 1 of the CovSocial project, n = 300 test persons (100 test persons per group) will be recruited for phase 2. All participants will receive an email inviting them to participate in phase 2. In addition to general information, this email also contains inclusion and exclusion criteria as well as a link to the online questionnaire for pre-screening, which is carried out before inclusion in the study. This is done via an online questionnaire (located on a secure server of the Max Planck Society). If they are interested in participating and have filled out a consent form for participation in the online screening, the test persons from phase 1 can fill out this questionnaire. They have 14 days to do so after receiving the invitation by email. The questions contained in the questionnaire refer to the inclusion and exclusion criteria. Subjects who meet the inclusion criteria (see below) will be invited by email to an online information evening, which will take place one month after receipt of the invitation email. Subjects who are still interested in participating in the intervention study after the information evening can finally register for the intervention study via a link in a subsequent email. In the week following the information evening, they will have 30-minute telephone conversations with trained mindfulness trainers. Following the telephone calls, the final assignment of the participants to the three groups will take place. The allocation will be done after stratified randomisation based on relevant variables and the subjects will give their consent to participate in the main study of phase 2 of the CovSocial project. The main study consists of 2, optionally 3, study full stops: pre-test, post-test 1 and post-test 2. Group I and II will conduct the intervention between pre-test and post-test 1. For group III, the intervention takes place after post-test 1.  **Figure 2**: *Testing procedure in phase 2 of the CovSocial project*.  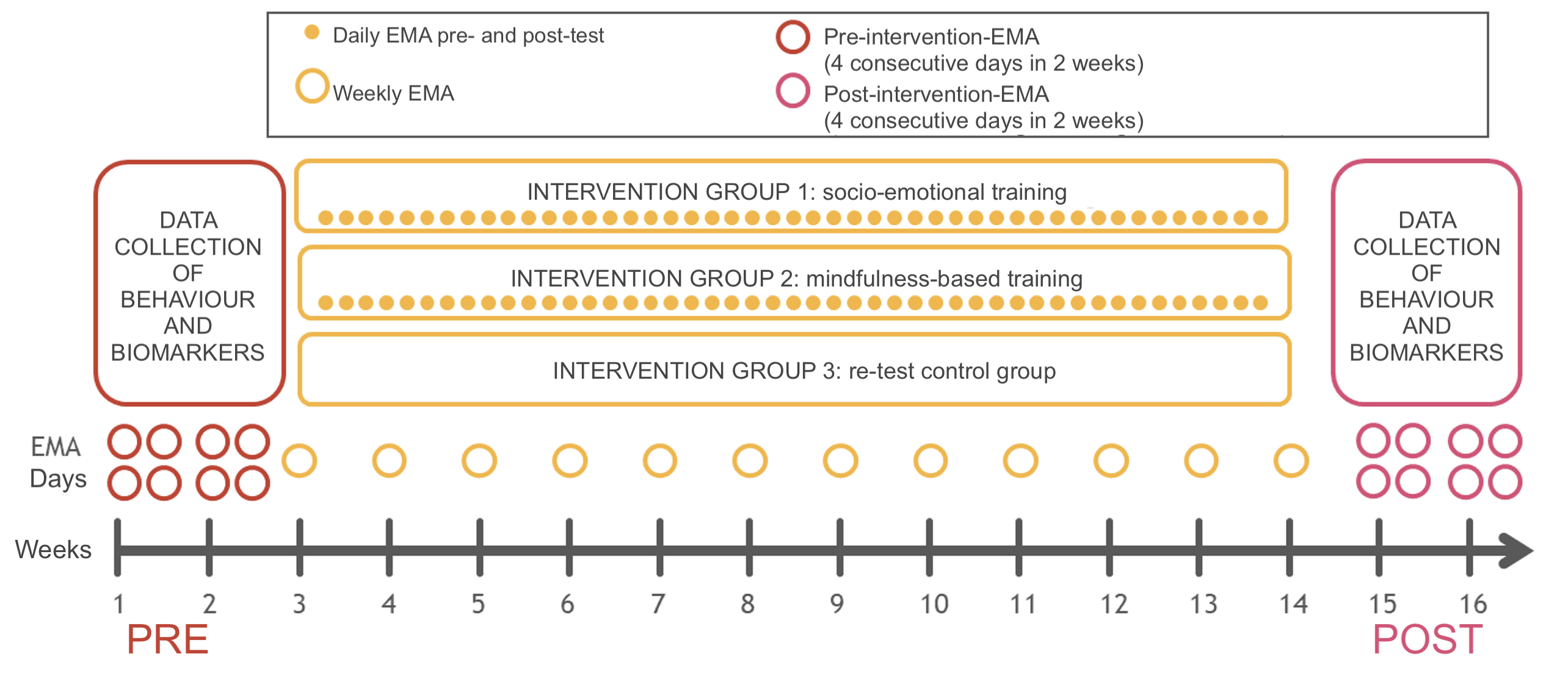  **Examination methods**:  a) **Pre-screening**  The questions related to exclusion and inclusion criteria will be completed by all interested participants from phase 1 of the CovSocial project.  b) **Pre- and post-testing**  Ecological Momentary Assessment, "EMA": Via the CovSocial app, participants will be asked questions about their current state of health on four consecutive days in two consecutive weeks and once for the first saliva survey on stress-relevant measures and COVID-relevant information (such as sleep quality, hormonal cycle, current illness, work shift and work day/weekend, belonging to COVID-19 risk group, positive test result for COVID-19, vaccination status). These four days are distributed over two weekdays and two weekend days in order to cover as broad a spectrum as possible in the participants' experiences and feelings in everyday life and relate to the following areas of life: sleep (only at the first time of the day), stress, affective mood, valence and temporal and social reference of their own thoughts, loneliness.  Answering the questionnaires takes about 2 minutes each. In addition, the participants are asked to collect ~~five~~ four saliva samples ~~per day~~ on two days (Thursday and Friday) (immediately after awakening, +30, +60, ~~+240 and +300~~ minutes after awakening and 1x in the evening). The materials for this will be given to them when they take part in the on-site examination of the behavioural data or if this date is after the scheduled EMA examination, sent to them by post. The participants bring these collected materials back to the research laboratory of the Social Neuroscience Research Group at the end of the pre or post-test or send them by post as well.  ~~Blood sample: For the pre-test, 50 ml of blood is obtained by peripheral venepuncture to determine the following markers:~~  ~~- endocrine markers: e.g. cortisol, DHEAS, adrenaline, noradrenaline~~  ~~- immunological markers: pro and anti-inflammatory cytokines, CRP~~  ~~- metabolic markers: e.g. adipokines, leptin, HbA1c, triglycerides, saturated and unsaturated fatty acids.~~  ~~- Markers of cell ageing: e.g. telomere length, mitogen-stimulated telomerase activity, P16INK4a, DNA repair capacity, p53.~~  ~~From this, an "allostatic load score" is formed, which maps the physiological consequences of chronic stress exposure across different systems (Juster et al., 2010).~~  A survey of genomic markers from saliva DNA: For the pre-test and post-test I, the following epigenetic markers are obtained using saliva samples:  - DNA methylation, genome-wide, measured e.g. with commercial methylation arrays, such as the Illumina EPIC DNA Methylation Array and targeted analyses using "targeted bisulfite sequencing".  A survey of inflammatory markers from a saliva sample: For the pre-test and post-test, the following inflammatory marker is obtained by means of one saliva sample each:  - C-reactive protein (CRP)  Questionnaires: The following questionnaires will be completed online via the CovSocial project web app for pre-test, post-test 1 and post-test 2. Standardised questionnaires are used to assess psychological and behavioural factors:  - Perceived Stress Scale (PSS-10; Cohen et al., 1983; Cohen & Williamson; 1988).  - UCLA Loneliness Scale (Döring & Bortz, 1993; Russell, Peplau & Cutrona, 1980)  - Beck Depression Inventory-II (BDI-II; Beck, Steer & Brown, 1996)  - Connor-Davidson Resilience Scale (CD-RISC; Connor & Davidson, 2003)  - State-Trait Anxiety Inventory (STAI; Spielberger, 2010)  - Cognitive Emotion Regulation Questionnaire (CERQ; Garnefski, Kraaij, Spinhoven, 2001; Loch, Hiller & Witthöft, 2011)  - Difficulties in Emotion Regulation Scale (DERS; Kaufman et al., 2016)  - Brief Resilience Scale (BRS; Chmitorz et al., 2018; Smith et al., 2008)  - Prosocialness Scale for Adults (PSA; Caprara et al., 2005)  - Self-Compassion Scale (SCS; Hupfeld & Ruffieux, 2011; Raes et al., 2011)  - Fear of Compassion Scale (FoC; Gilbert et al., 2012)  - Saarbrücken Personality Questionnaire/Interpersonal Reactivity Index (IRI; Davis, 1980; Paulus, 2009)  - Social Value Orientation (Murphy, Ackermann & Handgraaf, 2011)  - Multidimensional Assessment of Interoceptive Awareness (MAIA; Mehling et al., 2018)  - Sussex-Oxford Compassion Scales (Gu et al., 2019)  *Behavioural data*: Behavioural data is collected using computer-based experiments in the research laboratory. These collect data on the following factors:   - EmpaToM (Böckler et al, 2014). - ReSource Project game-theoretic paradigms on altruistic prosocial behaviour (e.g. Böckler et al., 2018; Böckler, Tusche & Singer, 2016; Leiberg, Klimecki & Singer, 2011; Singer et al., 2016). - Dot probe task (Macleod, Matthews & Tata, 1986) - Scrambled Sentences Task (Everaert et al., 2014) - ~~Affective Flexibility Task (Malooly, Genet & Siemer, 2013)~~ - ~~Implicit Association Test (Greenwald et al., 2003)~~   Data on early childhood stress, subjective feelings of stress and mental and physical health during the pandemic are available from CovSocial Phase 1.  **c) Intervention**: The intervention phase consists of 10 weeks each. Online mental exercises (socio-emotional exercise, the affect dyad, for group I and mindfulness-based exercises such as breathing meditation for group II) are conducted six times a week. Both exercises are conducted via the CovSocial mobile app. The affect dyad consists of a 12-minute exercise in pairs. The breathing meditation consists of a 10-minute exercise done alone. Before and after the exercises, a short daily pre-post exercise EMA takes place via the app. A detailed protocol of both interventions is provided. In addition, once a week, 2-hour online meetings take place in which the intervention is practised with trained meditation teachers, feedback is given, and questions can be asked. During the intervention phase, a questionnaire survey is also conducted once a week via the app. On this day, the participants will receive questions about their current state of mind, especially regarding stress, emotion regulation, worries, psychological flexibility, interoception and compassion, which they can answer via the CovSocial app on their mobile phone or computer. The following questionnaires are used for this purpose:   - Mindfulness (CAMS-R) - CERQ (subscales Acceptance, Rumination, Positive Reappraisal) - Perceived Stress Scale - 4 (PSS-4) - Fear of Compassion Scale (FoC; Gilbert et al., 2012) ~~subscale~~items fear of expressing compassion for others and oneself - State Self-Compassion Scale (SCS; Hupfeld & Ruffieux, 2011; Raes et al., 2011) subscale self-kindness - Sussex-Oxford Compassion Scales (Gu et al., 2019) subscales feeling for the person suffering (self) und tolerating uncomfortable feelings (other) - IRI (Davis, 1980; Paulus, 2009) subscales Personal Distress and Empathic Concern - Cube of Thoughts (Kok & Singer, 2017) - Worry (Penn State Worry Questionnaire, Kertz et al., 2014) - Cognitive Control and Flexibility Questionnaire (Gabrys et al., 2018) - Acceptance and Action Questionnaire (AAQ-II; Bond et al., 2011) - Coping strategies (Brief COPE; Carver, 1997) - 4 stress assessment questions (Smyth et al., 2017) - Multidimensional Assessment of Interoceptive Awareness (MAIA; Mehling et al., 2018) - Three self-generated questions on frequency and quality of social interaction and empathic listening - Social belonging questions as in phase 1 of the CovSocial project: - Draw the circles to best represent your belonging to the following groups. - "Note: The closer you draw the circles to each other, the more you felt you belonged to this group in January 2020." - Me and family - Me and friends - Me and the neighbourhood - Me and the Berlin population - Me and the German population - Me and European population - Me and world population   **Post-testing**: The post-testing is identical to the pre-measurement.  *Literature:*  Beck, A. T., Steer, R. A., & Brown, G. (1996). Beck depression inventory–II. *Psychological Assessment*.  Berg, J., Dickhaut, J. & McCabe, K. (1995). Trust, Reciprocity, and Social History. *Games and Economic Behavior, 10*, 122-142.  Böckler, A., Tusche, A., Schmidt, P., & Singer, T. (2018). Distinct mental trainings differentially affect altruistically motivated, norm motivated, and self-reported prosocial behaviour. *Scientific Reports, 8:*13560*.*  Böckler, A., Kanske, P., Trautwein, F.-M., & Singer, T. (2014). *The EmpaToM: A novel fMRI-task separating affective and cognitive routes to social cognition.* Poster presented at 20th Annual Meeting of the Organization for Human Brain Mapping (OHBM), Hamburg, Germany.  Bond, F. W., Hayes, S. C., Baer, R. A., Carpenter, K. M., Guenole, N., Orcutt, H. K., ... & Zettle, R. D. (2011). Preliminary psychometric properties of the Acceptance and Action Questionnaire–II: A revised measure of psychological inflexibility and experiential avoidance. *Behavior Therapy, 42(4),* 676-688.  Caprara, G. V., Steca, P., Zelli, A., & Capanna, C. (2005). A new scale for measuring adults' prosocialness. *European Journal of psychological assessment*, *21*(2), 77-89.  Carver, C. S. (1997). You want to measure coping but your protocol’too long: Consider the brief cope. *International Journal of Behavioral Medicine, 4(1),* 92-100.  Chmitorz, A., Wenzel, M., Stieglitz, R. D., Kunzler, A., Bagusat, C., Helmreich, I., ... & Lieb, K. (2018). Population-based validation of a German version of the Brief Resilience Scale. *PloS one*, *13*(2), e0192761.  Cohen S, Kamarck T, Mermelstein R, 1983. A global measure of perceived stress. J Health Soc Behav 24, 385–396.  Cohen S, Williamson G, 1988. Perceived stress in a probability sample of the United States., in: Spacapan S, Oskamp S (Eds.), The Social Psychology of Health. Sage, Newbury Park, CA.  Connor, K. M., & Davidson, J. R. (2003). Development of a new resilience scale: The Connor‐Davidson resilience scale (CD‐RISC). *Depression and Anxiety, 18(2),* 76-82.  Davis, M. H. (1980). A multidimensional approach to individual differences in empathy. *JSAS Catalog of Selected Documents in Psychology, 10,* 85*.*  Döring, N., & Bortz, J. (1993). Psychometrische Einsamkeitsforschung: Deutsche Neukonstruktion der UCLA Loneliness Scale. *Diagnostica, 39(3)*, 224-239.  Engert, V., Kok, B. E., Papassotiriou, I., Chrousos, G. P., & Singer, T. (2017). Specific reduction in cortisol stress reactivity after social but not attention-based mental training. *Science Advances,* *3*(10): e1700495.  Everaert, J., Duyck, W., & Koster, E. H. (2014). Attention, interpretation, and memory biases in subclinical depression: A proof-of-principle test of the combined cognitive biases hypothesis. *Emotion, 14(2),* 331.  Gabrys, R. L., Tabri, N., Anisman, H., & Matheson, K. (2018). Cognitive control and flexibility in the context of stress and depressive symptoms: The cognitive control and flexibility questionnaire. *Frontiers in Psychology, 9*, 2219.  Garnefski, N., Kraaij, V., & Spinhoven, P. (2001). Negative life events, cognitive emotion regulation and emotional problems. *Personality and Individual differences*, *30*(8), 1311-1327.  Gilbert, P., McEwan, K., Gibbons, L., Chotai, S., Duarte, J., & Matos, M. (2012). Fears of compassion and happiness in relation to alexithymia, mindfulness and self-criticism. *Psychology and Psychotherapy*, *85*, 374–390. DOI:10.1111/j.2044-8341.2011.02046.x  Greenwald, A. G., Nosek, B. A., & Banaji, M. R. (2003). Understanding and using the implicit association test: I. An improved scoring algorithm. *Journal of personality and social psychology*, *85*(2), 197.  Gu, J., Baer, R., Cavanagh, K., Kuyken, W., & Strauss, C. (2019). [Development and psychometric properties of the Sussex-Oxford compassion scales (SOCS)](http://www-2.omcmultisite.wpengine.com/wp-content/uploads/sites/14/2020/11/SOCS-Paperpdf.pdf). Assessment, 27(1), 3-20.  Hupfeld, J. & Ruffieux, N. (2011). Validierung einer deutschen Version der Self-Compassion Scale (SCS-D). *Zeitschrift für Klinische Psychologie und Psychotherapie, 40 (2),* 115–123.  Juster, R. P., McEwen, B. S., & Lupien, S. J. (2010). Allostatic load biomarkers of chronic stress and impact on health and cognition. *Neuroscience & Biobehavioral Reviews*, *35*(1), 2-16.  Kaufman, E.A., Xia, M., Fosco, G., Yaptangco, M., Skidmore, C.R., & Crowell, S. (2016). The Difficulties in Emotion Regulation Scale Short Form (DERS-SF): validation and replication in adolescent and adult samples. *Journal of Psychopathology and Behavioral Assessment, 38(443*), 443-455.  Kertz, S. J., Lee, J., & Björgvinsson, T. (2014). Psychometric properties of abbreviated and ultra-brief versions of the Penn State Worry Questionnaire. *Psychological assessment, 26(4),* 1146.  Kok, B. E., & Singer, T. (2017). Effects of contemplative dyads on engagement and perceived social connectedness over 9 months of mental training: A randomized clinical trial. *JAMA Psychiatry,* *74*(2), 126−134.  Leiberg, S., Klimecki, O., & Singer, T. (2011). Short-Term Compassion Training Increases Prosocial Behavior in a Newly Developed Prosocial Game. *PLoS ONE 6(3)*: e17798. https://doi.org/10.1371/journal.pone.0017798  Loch, N., Hiller, W., & Witthöft, M. (2011). Der cognitive emotion regulation questionnaire (CERQ). *Zeitschrift für Klinische Psychologie und Psychotherapie*.  Macleod, C., Mathews, A., & Tata, P. (1986). Attentional bias in emotional disorders. *Journal of Abnormal Psychology, 95(1),* 15–20.  Malooly, A. M., Genet, J. J., & Siemer, M. (2013). Individual differences in reappraisal effectiveness: the role of affective flexibility. *Emotion, 13(2),* 302.  Murphy, R. O., Ackermann, K. A., & Handgraaf, M. (2011). Measuring social value orientation. *Judgment and Decision making, 6(8),* 771-781.  Neff, K.D., Tóth-Király, I., Knox, M.C., Kuchar, A., & Davidson, O. (2021) The development and validation of the State Self-Compassion Scale (Long-and Short Form). *Mindfulness, 12(1),* 121-140.  Paulus, C. (2009). *Der Saarbrücker Persönlichkeitsfragebogen SPF (IRI) zur Messung von Empathie: psychometrische Evaluation der deutschen Version des Interpersonal Reactivity Index*. URL: http://psydok. sulb. uni-saarland. de/volltexte/2009/2363.  ~~Raes, F., Pommier, E., Neff,K. D., & Van Gucht, D. (2011). Construction and factorial validation of a short form of the Self-Compassion Scale.~~ *~~Clinical Psychology & Psychotherapy, 18~~*~~, 250-255.~~  Russell, D., Peplau, L. A., & Cutrona, C. E. (1980). The revised UCLA Loneliness Scale: concurrent and discriminant validity evidence. *Journal of personality and social psychology*, *39*(3), 472.  Singer, T., & Engert, V. (2019). It matters what you practice: Differential training effects on subjective experience, behavior, brain and body in the *ReSource Project*. *Current Opinion in Psychology, 28,* 151–158.  Singer, T., Kok, B. E., Bornemann, B., Zurborg, S., Bolz, M., & Bochow, C. (2016). *The ReSource Project: Background, design, samples, and measurements*. Max Planck Institute for Human Cognitive and Brain Sciences, Leipzig.Smith, B. W., Dalen, J., Wiggins, K., Tooley, E., Christopher, P., & Bernard, J. (2008). The brief resilience scale: assessing the ability to bounce back. *International journal of behavioral medicine*, *15*(3), 194-200.  Smyth, J. M., Zawadzki, M. J., Juth, V., & Sciamanna, C. N. (2017). Global life satisfaction predicts ambulatory affect, stress, and cortisol in daily life in working adults. *Journal of Behavioral Medicine, 40(2*), 320-331.  Spielberger, C. D. (2010). State‐Trait anxiety inventory. *The Corsini encyclopedia of psychology*, 1-1. |
| 9. Evaluation and weighing of the foreseeable risks and disadvantages of study participation against the expected benefits for the study participants and persons who will become ill in the future (risk-benefit balance). | We do not expect any potential disadvantages for the study participants. ~~The potential risks are very low (see below). The invasive method of blood sampling will be performed by experienced medical staff. The intervention will be carried out by trained professionals (i.e. trainers of mindfulness-based practices) to minimise the risk to the participants.~~  The benefits for the participants are potentially very high due to the planned intervention to reduce loneliness and stress and to increase psychological well-being. |
| a. medical benefit to be tested for the study participants (individual benefit for the individual patient). | The potential benefit of the study results for the study participants is high, as participation in mental training programmes to reduce stress and loneliness and to increase social cohesion can improve mental well-being and resilience and prevent the development of mental disorders as a result of high-stress levels. |
| b. medical benefit to be tested for persons with the disease in the future (group benefit) | The potential benefit of the study results for the future for people with increased stress levels, social isolation and mental risk factors is high, as the findings of this study can be used to develop early prevention and intervention measures that can counteract the development of mental disorders as a result of high-stress levels and social isolation. |
| c. **Risks** and burdens for the study participants (list all in detail) | ~~Blood collection: Blood is collected via venepuncture, with rare risks of infection or injury to neighbouring tissue. Apart from a brief pain when the needle is inserted, there may occasionally be a slight haemorrhage, which disappears within a few days. Some people react to a blood draw with a circulatory reaction.~~  Questionnaires/interviews: Some of the questionnaires and interviews deal with information that is of a personal and private nature and which, if it were to get outside the research context, could pose a possible social risk. Also, some of the questionnaires may awaken negative memories and trigger possible feelings of shame, helplessness, anger, etc.  Saliva samples: There are no risks associated with taking saliva samples.  In general, participation in genetic testing or the collection of health data carries the risk of invasion of privacy, with possible negative social, psychological and economic consequences. However, the risk in this study is minimal for the following reasons: 1) The examination of the genetic material is not intended to diagnose genetic diseases. 2) All samples and genetic information will also be kept and analysed separately from your personal information at all times and will not be passed on to third parties. Therefore, there will be no personal feedback on the results of the genetic testing. 3) The persons measuring the genetic characteristics do not have access to your personal information and do not have access to the coding list (see below). A connection to the test persons can therefore not be established. 4) DNA samples will be destroyed 10 years after the end of the study and stored in an access-protected biobank repository (MPI Psychiatry) in pseudonymised form until then. **Based on the data, the risk of a direct inference to a person is not excluded after completion of the data collection but nevertheless minimised as best as possible.**  **Intervention**:  In the socio-emotional training with daily affect dyads, the loud narration of difficultly experienced situations can lead to negative emotions arising in both the listener and the speaker due to the narration of negative emotions, which can continue to have an effect even after the mental exercise. Mindfulness-based meditations can also be experienced as unpleasant or difficult for beginners.  However, in order to intercept and discuss possible negative experiences, weekly meetings with the teachers are scheduled. In addition, as described in detail below, a two-step screening process will be carried out to exclude subjects with pre-existing mental health conditions and mental instability prior to the study. |
| 10. Risk management measures | The data collected in the pre-screening, as well as the telephone calls made beforehand, are also intended to minimise the risk of persons with mental illness or mental instability participating in the intervention study.  Since there is a fundamental risk of data mishaps and misuse in the web-based collection and storage of data, technical and organisational measures are applied for data protection and data security in the sense of the EU-DSGVO. |
| 11. Termination criteria | Participation in this study is voluntary. The early withdrawal of a subject from the study is at the personal request of the subject, in case of loss of contact or after any event which, in the opinion of the study management, precludes further participation in the study. |
| 12. The number, age and sex of the persons concerned | The sample is recruited from the group of test persons who participated in phase 1 of the CovSocial project (adult Berliners, 18-65 years). From this sample of N = 3522 subjects, the invitation to participate in the prescreening questionnaire will be sent by e-mail. The aim is to randomly assign 300 subjects to the three groups (socio-emotional and mindfulness-based training, retest control). |
| 13. Biometric design with the indication of statistical methodology, including justification of the number of cases. Indication of the statistician(s).  (If advice is given by the Institute of Biometry of the Charité, a signature must be inserted). | To test for intervention effects, mixed models are used in which measurement time (pre-test and post-test 1) and group (group I: socio-emotional intervention, group II: mindfulness intervention, group III: re-test control group + socio-emotional intervention) and their interaction are defined as fixed effects, and random effects (within-subjects) are defined via participant ID. A priori test strength analyses were based on previous results from a mental training study, the ReSource project (Singer et al., 2016), which included the planned mental training interventions as items. The effect sizes of the effects following the more extensive 3-month training modules were found to be low to medium in the respective cohorts of n=80 tested (e.g. Hildebrandt et al., 2017; Singer & Engert, 2019). It is possible that the intervention effects of the socio-emotional and mindfulness-based online interventions may be lower without the context of other intervention elements. The computer programme G*Power (Faul et al., 2007) was used to calculate a priori test power analyses. Here, reference was made to analyses of variance with repeated measures and interactions of between-group and within-group variables. Based on an alpha level of .05 and with a power (1-ß) of .80, 3 groups and 2 measurement time points, a correlation of variables with measurement repetition of .39, which could be determined as the lower limit of retest reliability for the Cortisol Awakening Response (CAR) (Pruessner et al., 1997), and an assumed small effect size of f = .10, this results in a sample size of n = 297. With a planned total case number of n = 300, small effect sizes of up to f = .07 can be recorded for the psychological questionnaires used, which are characterised by higher retest reliabilities of at least .70. With the addition of the third measurement time point (post-test 2), under the same assumptions (number of cases n=300, power (1-ß) = .80, α= .05), an effect of strength f = .09 can be recorded for measurement methods with a retest reliability of .39 and an effect of strength f = .06 for measurement methods with a retest reliability of .70.  For genetic markers, our hypotheses (H9) concern associations between genetic data from phase 1 of the CovSocial project and the intervention effects formulated under hypotheses 1-6. As these genetic data have already been collected, no biometric design is required for these questions.  Given the relative lack of studies examining the impact of mental training interventions on changes in DNA methylation over time, it is difficult to estimate expected effect sizes. Hypotheses (H9-H10) on pre-post intervention changes in epigenetic markers are therefore exploratively analysed.  *Literature*  Faul, F., Erdfelder, E., Lang, A.-G., & Buchner, A. (2007). G*Power 3: A flexible statistical power analysis program for the social, behavioral, and biomedical sciences. *Behavior Research Methods, 39,* 175-191.  Hildebrandt, L. K., McCall, C., & Singer, T. (2017). Differential effects of attention-, compassion-, and socio-cognitively based mental practices on self-reports of mindfulness and compassion. *Mindfulness, 8(6),* 1488-1512.  Pruessner, J.C., Wolf, O.T., Hellhammer, D.H., BuskeKirschbaum, A., von Auer, K., Jobst, S., Kaspers, F., Kirschbaum, C., 1997. Free cortisol levels after awakening: a reliable biological marker for the assessment of adrenocortical activity. Life Science, 61, 2539–2549.  Singer, T., & Engert, V. (2019). It matters what you practice: Differential training effects on subjective experience, behavior, brain and body in the ReSource Project. Current Opinion in Psychology, 28, 151–158.  Singer, T., Kok, B. E., Bornemann, B., Zurborg, S., Bolz, M., & Bochow, C. (2016). *The ReSource Project: Background, design, samples, and measurements*. Max Planck Institute for Human Cognitive and Brain Sciences, Leipzig. |
| 14.  a. Presentation and (if applicable) explanation of the **inclusion and exclusion criteria**. | Inclusion criteria   - Participation in phase 1 - Ability to give consent - Written informed consent - Age 18-65 years - Berlin general population - German language comprehension required for participation in the language-based intervention and independent completion of the questionnaires   Exclusion criteria   - Lack of technical equipment: no access to the internet or no mobile phone to use an app - Psychology students - Experience with spiritual practice - Regular yoga practice with meditative components within the last two years - Medication with an effect on the physiological markers collected - Acute psychotherapeutic treatment or diagnosis of a mental disorder within the last two years - Diagnosis of a psychotic disorder - Serious illness or chronic pain - Neurological impairment - Alexithymia (TAS-20 > 60) - Elevated scores on scales of depressive (PHQ-9 > 19) and anxiety symptoms (GAD-7 > 15) - Participation in other stress reduction programmes - Suicidality (measured with PHQ-9 item) - Telephone teacher mental health assessment or contraindication after individual screening - Current participation or plans to participate in other programmes with goals comparable to those of this study. |
| b. **Study information** (who gives this verbally and in writing and indication of how much time is left between explanation and consent  (Written information as an attachment) | The participation information for this study is provided in writing at the beginning as part of the online-based data collection. **"Participation information pre-screening" and "Participation information study" provided as attachments to ethics committee.**  There will be two separate participation forms: One for the pre-screening and another for the final sample of participants at the beginning of the intervention phase. |
| c. **Consent form** (written form as attachment) | **"Consent form for pre-screening" and "Consent form for study" provided as attachments to ethics committee.**  There will be two separate consent forms: One for the pre-screening and another written one for the final sample of participants at the beginning of the intervention phase.  The consent forms will be provided online. |
| d. If applicable, **information and consent of the legal representative** (if applicable also description of the procedure for establishing judicial care). | Not applicable |
| 15. Methods to recruit study participants (notice board?, newspaper advertisements? Etc.) | All subjects who gave their consent to participate in further phases of the study at the beginning of phase 1 of the CovSocial study ("Online survey of social cohesion and mental health during the SARS-CoV-2 pandemic") will be contacted by email. |
| 16. If applicable, **the reason for inclusion and demonstration of therapeutic benefit for persons who are minors and/or unable to consent**. | Not applicable |
| 17. Relationship between study participant and study doctor (is the study doctor also the treating doctor?). | Not applicable  This is not a clinical trial population. |
| 18. Statement on the involvement of persons who may be dependent on the sponsor. | Sponsor-dependent subjects will not be included in the study. |
| 19. Measures that allow a determination of whether a study participant is participating in more than one study at the same time or before the end of a period specified in the previous study.  Is participation in more than one study possible? | As part of the pre-screening, the subjects are asked whether they are currently participating in other programmes whose objectives are comparable to those of the study presented here or whether they are planning to do so. |
| 20. If applicable: remuneration or reimbursement of study participants (amount, what should be paid for?). | Participation in the study is compensated according to the time spent by the volunteers. The time for the questionnaires, the surveys in the laboratory (behavioural tests and blood samples), saliva samples at home and a flat-rate travel time of 30 minutes/trip will be compensated.  This is expected to be 5.5 hours for the pretesting. For the intervention phase, the time spent on the daily/weekly questions via the web app will be compensated. This amounts to approx. 20 min per week, corresponding to 3.5 hours for the 10-week intervention period. Post-testing is expected to be approximately 5.5 hours. The amount of compensation is 10€ per hour.  Considering the time spent for the study participation on the part of the test persons, this amount (10€/hour) is an adequate compensation, but at the same time does not motivate participation for financial reasons. The expense allowance is transferred to the bank account of the respective volunteers. The volunteers fill out an application for reimbursement. |
| 21. If applicable: plan for the further treatment and medical care of the persons concerned after the end of the study. | Not applicable |
| 22. If applicable: insurance of the study participants  (Confirmation of insurance and insurance conditions, insurer, scope of insurance, duration of insurance). | Not applicable |
| 23. Documentation procedures:  - Reference to CRF forms, if applicable.  - Indication of data to be recorded  - Sample handling  - Retention / archiving (including time limits)  - Access to data and samples | The collected personal and pseudonymised data are documented in electronic form.  The study data include:  - Questionnaires/interview data in digital form.  - Biological samples  - Behavioural data from computer-based experiments and related questions and instructions on paper sheets.  The data is processed and stored in encrypted form. For this purpose, the study management assigns a code number to the data (pseudonymisation of the data). Only the study management has access to the code key, which allows the study-related data to be linked to the personal data. There is only one coding list, which is kept locked away by the study management.  The study participants have the right to access all their personal data. They also have the right to correct any inaccuracies in their personal data and can object at any time to the further processing of their data collected as part of the study and demand their deletion or destruction. This is possible as long as the coding list exists.  All questionnaires/interviews, data from the electronic questionnaires, the biological samples, and the behavioural data as well as associated paper questionnaires will be kept without personal identifiers only with the code and will only be accessible to members of the study team.  The genetic samples will be sent to a laboratory in Munich to the cooperation partner of the project, Prof. Dr. Elisabeth Binder, for genetic and epigenetic evaluations. The samples are sent exclusively in pseudonymised form.  All other biological samples are stored in freezers located in the laboratory of the Social Neuroscience Research Group of the Max Planck Society. These biological samples are analysed pseudonymously at the Institute for Medical Psychology at the Charité. The rooms where the freezers are located are locked and only employees of the research laboratory have access.  The research results are stored and processed electronically. Further information on data protection, storage and archiving can be found under 26. and 27. |
| 24. If applicable, a description of how the health status of healthy affected persons is to be documented. | The health status of the participants is assessed in the pre-screening by means of questionnaires according to subjective self-reporting. |
| 25. If applicable: methods of identifying, documenting and reporting adverse events (when, by whom and how?). | Not applicable |
| 26. procedure for protecting the confidentiality of the stored data, documents and, if applicable, samples, presentation of the pseudonymisation or anonymisation of the data and samples of study participants (initials and date of birth as coding scheme are not permissible!).  - Description of the separation of medical records, study documentation and allocation of personal data  - Designation of access rights including access to participant identification lists during and after the study conduct  - Detailed specification of the procedures for transmission, encryption, restriction of processing (blocking) and deletion (including specification of the network structure and servers used, if applicable).  -If applicable, access to identifying data for legally authorised examiners (third-parties) for the purpose of inspecting the files required for this purpose. | The legal provisions of data protection according to EU-DSGVO as well as the Berlin State Data Protection Act and the Gene Diagnostics Act that apply to the study are fulfilled for all personal data.  As this is a continuation of the CovSocial research project, personal identifying data already collected exists. This includes email addresses, names and addresses of the participants. In this part of the study, therefore, only the bank details of the test persons are newly requested for the payment of the expense allowance. For this purpose, the volunteers will receive an application form for reimbursement of the expense allowance via the web app and/or by email, which they can fill out with their bank details and print out. They must sign the form and bring it with them to the pre-test or post-test. With their signature, the volunteers confirm the accuracy of the information and allow it to be forwarded to the accounting department responsible for the Social Neuroscience Research Group. The forwarding is done by the administrative staff of the Social Neuroscience Research Group.  In the first study phase of the project, a pseudonymous identification number was generated for each participant, which does not allow any conclusions to be drawn about the identity of the study participants. Subsequently, only this number will be used to identify all data collected. The identification number and personal information are stored on an access-secured server of the MPG (subserver 1). The data collection and storage take place on a separate server of the MPG (subserver 2), on which the web application is also installed. The web application was programmed by the digital agency CosmoCode, which was commissioned by the Max Planck Society. The saliva samples and blood samples are provided with a separate code key (dual coding). The coding list, which allows the link between the genetics ID/~~blood ID~~/cortisol ID and the study ID to be established, is stored on subserver 1 of the MPG and is only accessible to certain persons specified by the study management and the study management. ~~The coding list of the blood ID, as well as the contact data of the subjects, is made available to the Institute of Medical Psychology via an encrypted channel. It will be password-protected and only accessible to the administrative assistant of the Institute for scheduling the laboratory visit for blood collection. The samples will be stored in the laboratories where the analysis takes place (Institute of Psychiatry - MPG and Institute of Medical Psychology - Charité) in access-proof freezers in lockable rooms.~~  Access to and transmission of exclusively pseudonymised study-related data from another MPG server to the CovSocial project staff takes place via an encrypted connection to the scientific network. MPG-external cooperation partners at Charité - Universitätsmedizin Berlin and Humboldt-Universität zu Berlin can use the pseudonymised data for scientific purposes at their respective institutions in accordance with DSGVO-compliant guidelines.  The data is evaluated pseudonymously and stored for 10 years. After this period, the coding list, which allows the study-related data to be linked to the personal and identifying data, is deleted.  Printed, written records are kept in lockable rooms and cabinets to which only study participants have access. Again, personal data will be kept separate from study data. Only staff selected by the study management have access to the personal data, but they do not have access to the study data. Only users with the appropriate authorisation have access to the study data. The allocation of access rights is delegated by the study management at the respective sites to the corresponding study staff. |
| 27. Declaration of compliance with data protection  - Assurance that all data collected and stored about the study participant will be treated confidentially (data secrecy and medical confidentiality).  - Assurance that the identifying data will be accessible only to the study director or to staff appointed by him/her.  - Indication of the measures taken to ensure confidentiality  - Measures for the data protection-compliant transmission of data that do not allow third-parties to establish a personal reference.  - Information on information, revocation, correction and deletion options,  - Measures to ensure the rights of participants.  - If transfers to non-EU countries are planned: Measures to ensure compliance with data protection (e.g. existence of an adequacy decision by the EU Commission or explicit consent of the study participants to such transfers. | The protection of the personal data of all study participants is guaranteed. The processing and publication of data are carried out exclusively in pseudonymised form.  All data will be treated confidentially in accordance with data protection legislation. All data will be physically or logically protected (as described under 26).  Between the Max Planck Society as the owner of the web app or mobile app used in this project and the digital service provider CosmoCode, there is a contract for commissioned data processing (GCP) in accordance with Art. 28 of the GDPR, which regulates the technical and organisational measures of the service provider in terms of Art. 32 of the GDPR.  The confidentiality obligation is contractually regulated between the Max Planck Society and the MBSR trainers hired for the study, who conduct a telephone screening in individual conversations and weekly group monitoring conferences in groups (20-25 persons each) with the participants. In addition, there is a contract for commissioned data processing (AVV) in accordance with Art. 28 DSGVO, which regulates the technical and organisational measures of the service provider in terms of Art. 32 DSGVO.  The study participants are given the opportunity to revoke their consent to participate in the study at any time (even after the survey) without any disadvantages resulting from this. In this case, all personal identifying data will be deleted.  The test persons have the possibility to contact the study staff at any time.  Participation information and the consent form provided as attachments to ethics committee. |
| 28. Names and addresses of the institutions involved in the study as a study centre or study laboratory, as well as of the study directors and the study physicians  - Details of external service providers involved, including details of data access options. | **Study Centers**  Charité – Universitätsmedizin Berlin  Institute for Medical Psychology  Campus Charité Mitte  Luisenstraße 57  10117 Berlin  Research Group Social Neurosciences  Max Planck Society  Campus Nord, Haus 5  Humboldt-Universität zu Berlin  Philippstrasse 13  10099 Berlin  **Study Leaders CovSocial Project**  Prof. Dr. Tania Singer (Gastwissenschaftlerin)  Charité – Universitätsmedizin Berlin  Clinic for Psychiatry and Psychotherapy  Campus Charité Mitte  Charitéplatz 1  10117 Berlin  tania.singer@charite.de  Prof. Dr. Tania Singer (wissenschaftliche Leiterin)  Research Group Social Neurosciences  Max Planck Society  Campus Nord, Haus 5  Humboldt-Universität zu Berlin  Philippstrasse 13  10099 Berlin  E-Mail: singer@social.mpg.de  **Co-management Phase 2 stress-related biological markers**  ~~Prof. Dr. Sonja Entringer~~  ~~Charité – Universitätsmedizin Berlin~~  ~~Institut für Medizinische Psychologie~~  ~~Luisenstraße 57~~  ~~10117 Berlin~~  ~~E-Mail:~~ [~~sonia.entringer@charite.de~~](mailto:sonia.entringer@charite.de)  Prof. Dr. Christine Heim (Teilleitung Phase 2 stressbedingte biologische Marker)  Charité – Universitätsmedizin Berlin  Institute for Medical Psychology  Luisenstraße 57  10117 Berlin  E-Mail: [christine.heim@charite.de](mailto:christine.heim@charite.de)  **Further Cooperation Partners**  Prof. Dr. med. Mazda Adli (Phase 1)  Charité – Universitätsmedizin Berlin  Clinic for Psychiatry and Psychotherapy  Campus Charité Mitte  Charitéplatz 1  10117 Berlin  E-Mail: mazda.adli@charite.de  Prof. Dr. Manuel Voelkle  Humboldt-Universität zu Berlin  Faculty of Life Sciences  Institute for Psychology  Unter den Linden 6  10099 Berlin  E-Mail: manuel.voelkle@hu-berlin.de  Prof. Dr. Elisabeth Binder  Max Planck Institute for Psychiatry  Kraepelinstr. 2 - 10  80804 München  E-Mail: [binder@psych.mpg.de](mailto:binder@psych.mpg.de)  **External Service Groups**  CosmoCode GmbH  Prenzlauer Allee 36G  10405 Berlin  Telefon: +49 30 814 50 40 70  Telefax: +49 30 2809 7093  Mail: [info@cosmocode.de](mailto:info@cosmocode.de) |
| 29. Information on the suitability of the trial site, in particular on the adequacy of the resources and facilities available there and of the personnel available to conduct the clinical trial and on experience in conducting similar trials. | The people and professors involved in the study have many years of expertise in the field of psychobiological research and the implementation of intervention studies, which can be documented by diverse publications, book contributions and lectures by all partners. |
| 30. Agreement on the access of the investigator/principal investigator/lead investigator to the trial, to the data and to the publication policy.  - Publications in a form that does not allow conclusions to be drawn about the person. | The publication of results takes place exclusively in pseudonymised form. |
| 31. Information on the funding of the study: funding source (name and location) and amount of funding in €.  - If applicable, indication of the cost centre for ILV settlement of the fee. | Social Neuroscience Research Group  Max Planck Society  Campus North, House 5  Humboldt University Berlin  Philippstrasse 13  10099 Berlin  Charité - University Medicine Berlin  Institute for Medical Psychology  Luisstrasse 57  10117 Berlin  Max Planck Institute for Psychiatry  Kraepelstr. 2 - 10  80804 Munich |
|  |  |

Prof. Dr. Tania Singer ~~Prof. Dr. Sonja Entringer~~ Prof. Dr. Christine Heim

Study leader ~~Co-leading of stress-related~~ Co-leading of stress-related

~~biomarkers~~ biomarkers
