# Supplementary material for: Investigating differential effects of socio-emotional and mindfulness-based online interventions on mental health, resilience and social capacities during the COVID-19 pandemic: The study protocol
Source: PLoS One. 2021 Nov 4;16(11):e0256323. doi: 10.1371/journal.pone.0256323 (PMC8568275; doi:10.1371/journal.pone.0256323)
Supplement: S1 Appendix — (DOCX) [file pone.0256323.s006.docx]

**S1 Appendix**

*A depiction of the recruited sample and dropouts at every stage of the first phase of the study. Sample 1 indicates the final sample of participants that completed the first three retrospective assessment timepoints (T1-T3). Sample 2 indicates the participants who completed only the Demography and T1 questionnaires. A portion of the Sample 2 participants completed the T4-T7 longitudinal assessments in phase 1. “Sample 1 with Genetics and T1-T7” includes participants of Sample 1 who completed the retrospective T1-T3 assessments, the longitudinal T4-T7 assessment and who provided genetic assessment.*
